# Ribosomal modification protein rimK-like family member A activates betaine-homocysteine S-methyltransferase 1 to ameliorate hepatic steatosis

**DOI:** 10.1038/s41392-024-01914-0

**Published:** 2024-08-08

**Authors:** Han Yan, Wenjun Liu, Rui Xiang, Xin Li, Song Hou, Luzheng Xu, Lin Wang, Dong Zhao, Xingkai Liu, Guoqing Wang, Yujing Chi, Jichun Yang

**Affiliations:** 1 Department of Physiology and Pathophysiology, School of Basic Medical Sciences, State Key Laboratory of Vascular Homeostasis and Remodeling, Center for Non-coding RNA Medicine, Peking University Health Science Center, Beijing, 100191 China; 2 Department of Endocrinology, The Second Affiliated Hospital, School of Medicine, Zhejiang University, Hangzhou, 310058 China; 3 Medical and Health Analysis Center, Peking University, Beijing, 100191 China; 4 Department of Hepatobiliary Surgery, Xi-Jing Hospital, Fourth Military Medical University, Xi’an, 710032 China; 5 Department of Endocrinology, Beijing Luhe Hospital, Capital Medical University, Beijing, 101100 China; 6 Department of Hepatobiliary and Pancreatic Surgery, General Surgery Centre, First Hospital of Jilin University, Changchun, 130061 China; 7 Key Laboratory of Pathobiology Ministry of Education, College of Basic Medical Sciences, Jilin University, Changchun, 130012 China; 8 Department of Central Laboratory and Institute of Clinical Molecular Biology, Department of Gastroenterology, Peking University People’s Hospital, Beijing, 100044 China; 9 Department of Cardiology, Peking University Third Hospital, Beijing, 100191 China

**Keywords:** Endocrine system and metabolic diseases, Pathogenesis

## Abstract

Nonalcoholic fatty liver disease (NAFLD) is a serious threat to public health, but its underlying mechanism remains poorly understood. In screening important genes using Gene Importance Calculator (GIC) we developed previously, ribosomal modification protein rimK-like family member A (RIMKLA) was predicted as one essential gene but its functions remained largely unknown. The current study determined the roles of RIMKLA in regulating glucose and lipid metabolism. RIMKLA expression was reduced in livers of human and mouse with NAFLD. Hepatic RIMKLA overexpression ameliorated steatosis and hyperglycemia in obese mice. Hepatocyte-specific RIMKLA knockout aggravated high-fat diet (HFD)-induced dysregulated glucose/lipid metabolism in mice. Mechanistically, RIMKLA is a new protein kinase that phosphorylates betaine-homocysteine S-methyltransferase 1 (BHMT1) at threonine 45 (Thr45) site. Upon phosphorylation at Thr45 and activation, BHMT1 eliminated homocysteine (Hcy) to inhibit the activity of transcription factor activator protein 1 (AP1) and its induction on fatty acid synthase (FASn) and cluster of differentiation 36 (CD36) gene transcriptions, concurrently repressing lipid synthesis and uptake in hepatocytes. Thr45 to alanine (T45A) mutation inactivated BHMT1 to abolish RIMKLA’s repression on Hcy level, AP1 activity, FASn/CD36 expressions, and lipid deposition. BHMT1 overexpression rescued the dysregulated lipid metabolism in RIMKLA-deficient hepatocytes. In summary, RIMKLA is a novel protein kinase that phosphorylates BHMT1 at Thr45 to repress lipid synthesis and uptake. Under obese condition, inhibition of RIMKLA impairs BHMT1 activity to promote hepatic lipid deposition.

## Introduction

Nonalcoholic fatty liver disease (NAFLD) encompasses a spectrum from simple steatosis, non-alcoholic steatohepatitis (NASH), to cirrhosis and hepatocellular carcinoma (HCC).^1^ At present, about 25% of the global population has been estimated to suffer from NAFLD, ranging from 13% in Africa to 42% in South Asia,^1,2^ and its prevalence may increase up to 56% by 2030 in the main countries.^3^ NAFLD is highly correlated with diabetes, liver diseases and other diseases.^4^ Clearly, NAFLD has become one of the major public issues, and further intensive study on its underlying mechanism(s) is greatly needed.

Homocysteine (Hcy) is a non-essential, non-proteinogenic sulfur-containing amino acid, formed in all tissues and mainly metabolized in liver and kidney.^5^ Hcy clearing is dependent on the remethylation and transsulfuration pathways, which are controlled by three enzymes: methionine synthase (MS; or 5-methytetrahydrofolate-homocysteine S-methyltransferase, MTR), betaine-homocysteine S-methyltransferase (BHMT) and cystathionine β-synthase (CBS).^5,6^ Decreased expressions of these enzymes will elevate serum Hcy level to cause hyperhomocysteinemia (HHcy),^7,8^ which is a critical risk factor for many diseases including metabolic diseases.^9^ In human and animals with NAFLD, HHcy is associated with disease progression.^10,11^ So far, several mechanisms have been proposed to explain Hcy-induced hepatic steatosis, including cluster of differentiation 36 (CD36)-mediated lipid uptake^12^ and increased lipolysis in adipose tissues.^6^ However, given the deleterious impacts of HHcy in the progression of metabolic diseases, intensive study is still needed to clarify the distinct mechanism(s) of HHcy and the in-depth roles of Hcy in triggering dysregulated glucose and lipid metabolism.

MTR is one of the two enzymes responsible for Hcy remethylation, which converts Hcy to methionine (Met) using 5-methyltetrahydrofolate as methyl donor in a methylcobalamin-dependent manner, maintaining the normal levels of S-adenosylmethionine and involve in numerous epigenetic mechanisms.^13,14^ MTR plays a crucial role in maintaining adequate methionine levels and preventing homocysteine accumulation, thus protecting against Hcy-induced cardiovascular disease and other metabolic disorders, including liver-related outcomes.^15,16^ A deficiency in MTR can lead to hyperhomocysteinemia.^17^ Mutations in the MTR gene such as A410P, S437Y, S450H, H595P, and I804T will cause homocystinuria, hyperhomocysteinemia and hypomethioninemia.^15^

Another Hcy-remethylated enzyme BHMT includes two subtypes, designated as BHMT1 and BHMT2, respectively. BHMT2 uses S-methylmethionine (SMM) as methyl donor.^18^ The mouse BHMT2 gene shared 69% identity to the mouse BHMT1, and the human BHMT2 showed 82% amino acid identity to human BHMT1.^18^ So far, it is difficult to detect the stable expression of BHMT2 protein in mammalians, which may be due to its instability and easy degradation.^19^ As the key BHMT subtype, BHMT1 transforms Hcy into Met using betaine as methyl donor. BHMT1 expression was reduced in obese diabetic mouse livers.^11^ Betaine supplementation increased BHMT1 expression, and alleviated hepatic lipid accumulation and alcohol-induced lipolysis in adipose tissues with reduced serum Hcy level.^20,21^ Deletion of BHMT1 increased hepatic/serum Hcy levels, and led to severe liver injury and lipid deposition.^22^ BHMT1 transgenic mice exhibited mitigated hepatic steatosis induced by alcohol and Hcy.^23^ However, although BHMT1 is an important enzyme that controls Hcy metabolism, whether its enzymatic activity is regulated by protein modification(s) remains unknown.

So far, the functions of many protein-coding mRNAs remain unillustrated.^24,25^ One human proteomics database ProtemicsDB covers about 18097 proteins among the 19629 human genes annotated in Swiss-Prot were developed.^26^ However, only 10000 to 12000 proteins had annotated functions, and the biological functions of the other proteins remain unknown.^26^ Moreover, even among the proteins with annotated functions, many of them have very little function reported.^26^ This greatly limits the understanding on the mechanisms of many diseases. Clearly, to screen important new genes among those with little or no function annotated will shed light on the distinct mechanism(s) of important diseases including HHcy, NAFLD and diabetes. To address this issue, we had previously developed a computational method called Gene Importance Calculator (GIC) to predict the essentiality (importance) of both protein-coding mRNAs and lncRNAs based on key nucleotide sequence characters. For predicting importance of protein-coding genes, GIC outperformed well-established computational scores and CRISPR/Cas9 scores.^27^ The higher is the predicted score, the more essential is the target gene.^27^ During the prediction of protein-coding genes with little or no functions annotated in database using GIC, ribosomal modification protein rimK-like family member A (RIMKLA) was predicted as one gene with potential essentiality. RIMKLA, also known as family with sequence similarity 80, member A (FAM80A) or N-acetylaspartyl-glutamate synthetase A/II (NAAGS A/II), shares high conservation across mammalian species. RIMKLA was previously reported to exhibit ligase activity in central nervous system (CNS).^28^ So far, the only annotated function of RIMKLA is the ability of regulating neuron excitability and recognition in CNS.^29^

In the present study, we reported that RIMKLA expression is reduced in livers of human and mouse with NAFLD. Therefore, we aimed to determine the roles and mechanisms of RIMKLA in the pathogenesis and progression of NAFLD. Particularly, whether RIMKLA modulated BHMT1 activity to regulate Hcy and lipid metabolism would be stressed.

## Results

### RIMKLA expression is reduced in the livers of mice and patients with NAFLD

**Fig. 1:**
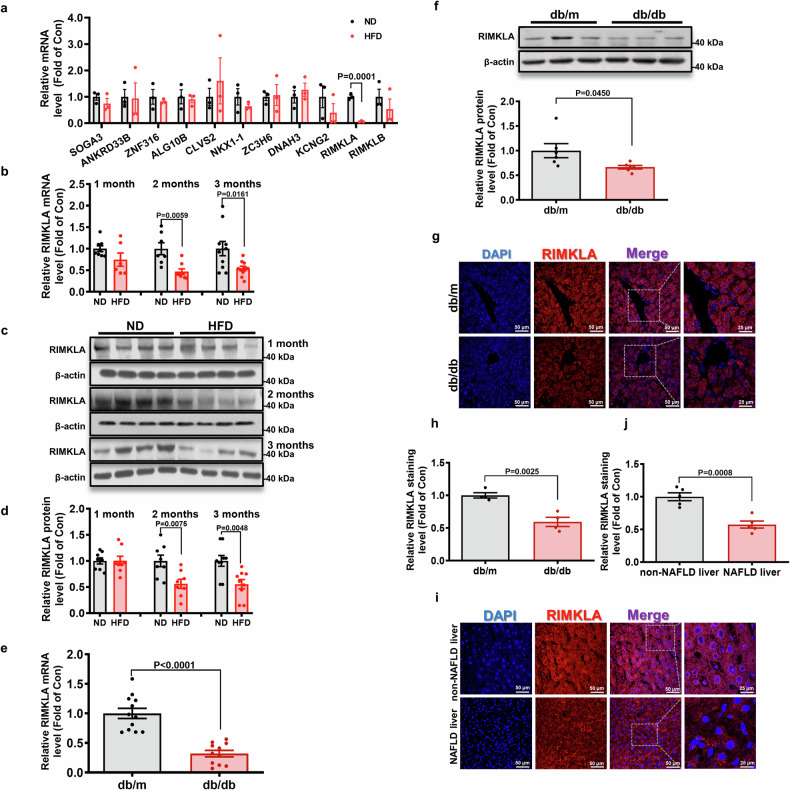
RIMKLA expression is reduced in the livers of obese mice and patients with NAFLD. **a** The mRNA levels of GIC-predicted genes in livers of HFD mouse in comparison with those of ND mice. *n* = 3. **b** RIMKLA mRNA level is reduced in livers of mice fed with HFD for 1, 2, and 3 months. *n* = 6–10. **c**, **d** RIMKLA protein level is reduced in livers of HFD mice. Representative gel images were shown on the panel **c**, and quantitative data shown in the panel **d**. *n* = 8–9. **e** RIMKLA mRNA level is reduced in livers of db/db mice. *n* = 11–12. **f** RIMKLA protein level is reduced in livers of db/db mice. Representative gel images were shown on the upper panel, and quantitative data shown in the lower panel. *n* = 6. **g**, **h** Immunofluorescent staining of RIMKLA in livers of db/db mice. Representative staining images were shown in panel **g**, and quantified data shown in panel **h**. *n* = 4. **i**, **j** Immunofluorescent staining of RIMKLA in livers of patients with NAFLD. Representative staining images were shown in panel **i**, and quantified data shown in panel **j**. *n* = 5. Blue, DAPI; Red, RIMKLA; Purple, Merge. Scale bar: 50 μm or 25 μm. All data were represented as mean ± SEM. Statistical *P* values were marked in each panel. Statistical *P* values for (**a**, **b**, **d**, **e**, **f**, **h**, **j**) were analyzed by student’s t-tests

**Fig. 2:**
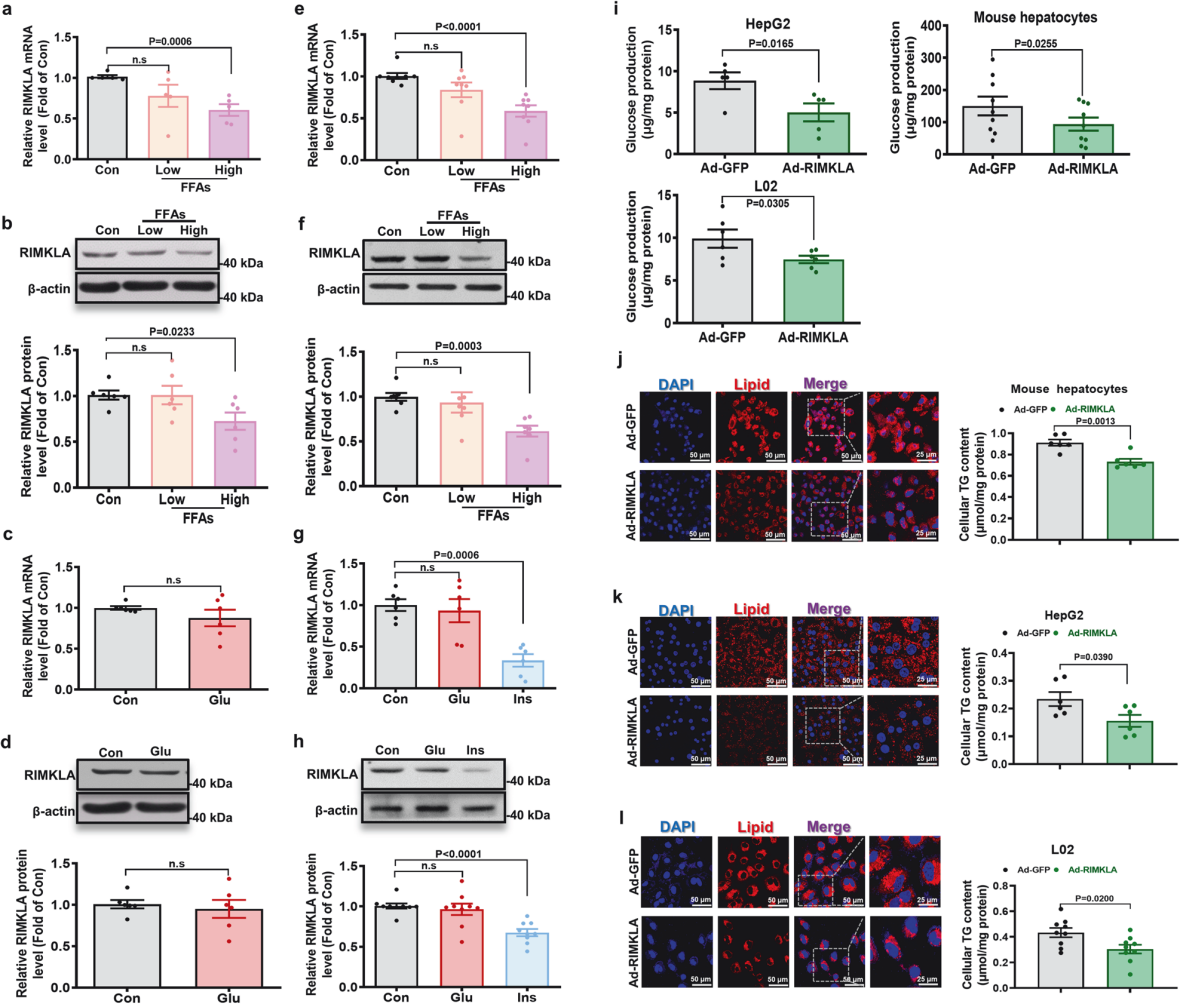
RIMKLA expression is inhibited by FFAs and insulin in cultured hepatocytes. **a**, **b** Treatment with FFAs reduced the mRNA (**a**) and protein (**b**) levels of RIMKLA in HepG2 cells. Cells were treated with low or high concentrations of fatty acids for 24 h before assays. Low: 0.1 mM oleic acid+0.2 mM palmitic acid. High: 0.2 mM oleic acid+0.4 mM palmitic acid. *n* = 5–6. **c**, **d** Glucose treatment for 24 h failed to affect RIMKLA mRNA (**c**) and protein (**d**) levels in HepG2 cells. Glu: Glucose (25 mM). *n* = 6. **e**, **f** Treatment with FFAs inhibited RIMKLA mRNA (**e**) and protein (**f**) levels in mouse primary hepatocytes. Cells were treated with low or high concentrations of fatty acids for 24 h. Low: 0.1 mM oleic acid+0.2 mM palmitic acid. High: 0.2 mM oleic acid+0.4 mM palmitic acid. *n* = 7–8. **g**, **h** Insulin but not glucose reduced the mRNA (**g**) and protein (**h**) levels of RIMKLA in mouse hepatocytes. Cells were treated with Glu (25 mM Glucose) or Ins (100 nM Insulin) for 24 h. *n* = 6–9. **i** RIMKLA overexpression suppressed glucose production in HepG2 cells, mouse hepatocytes, and L02 cells. *n* = 5-9. **j**–**l** RIMKLA overexpression alleviated FFAs-induced cellular lipid deposition in mouse hepatocytes cells (**j**), HepG2 (**k**), and L02 cells (**l**). Cells were treated with Ad-GFP or Ad-RIMKLA in the presence of 0.1 mM oleic acid+0.2 mM palmitic acid for 24 h. Representative fluorescent images were shown in left panel and quantitative data in right panel. *n* = 6–9. For (**j**–**l**), Blue, DAPI; Red, Lipid; Purple, Merge. Scale bar: 50 μm or 25 μm. All data were represented as mean ± SEM. Statistical *P* values were marked in each panel. n.s means no statistical significance. Statistical *P* values were marked in each panel. For statistical analysis, *P* values were calculated by Kruskal–Wallis test with Dunn’s multiple comparisons for (**a**, **b**, **e**–**h**) and student’s t-test for (**c**, **d**, **i**–**l**)

As GIC is a useful computational method developed in our previous study,^27^ we firstly screened genes with both the GIC score ≥ 0.8 and ≤ 5 publications in PubMed up to 2018. The top 10 genes were selected, and their mRNA expression levels were detected in the livers of mice fed on normal diet (ND or high-fat diet (HFD) for 3 months (Supplementary Table 1 and Fig. 1a). As a result, only the mRNA expression level of RIMKLA was decreased in HFD-mouse livers. RIMK family contains two members, designated as RIMKLA and ribosomal modification protein rimK-like family member B (RIMKLB), respectively. RIMKLB shares 65% sequence identity with RIMKLA and is mainly expressed in the CNS, placenta, and testis.^28^ RIMKLB mRNA expression level remained unchanged in HFD mouse livers (Fig. 1a). Furthermore, GIC prediction revealed that RIMKLA was more essential in humans (Top 2% among all human genes) and mice (Top 25% among mouse genes) than RIMKLB (Supplementary Fig. 1a). Then, we detected the expression profile of RIMKLA in mouse tissues, and demonstrated that RIMKLA mRNA is ubiquitously expressed across the main organs in mice (Supplementary Fig. 1b), and its mRNA level was reduced only in livers but not in other main metabolic tissues including pancreas, heart, white adipose, skeletal muscle, and cerebrum in mice fed on HFD for 3 months when compared with control mice (Supplementary Fig. 1c). Then we evaluated on the dynamic change of RIMKLA expression in livers of another set of mice fed on HFD for 1 month, 2 months and 3 months, respectively. The result revealed a stepwise decrease of RIMKLA expressions with the progression of metabolic disorders (Fig. 1b–d). RIMKLA expressions were similarly reduced in db/db mouse livers (Fig. 1e–h). Importantly, RIMKLA expression was also significantly reduced in livers of patients with NAFLD than that in individuals with non-NAFLD associated with HCC (Fig. 1i, j). RIMKLA expression was reduced after treatment with free fatty acids (FFAs) but not glucose in HepG2 cells (Fig. 2a–d) and primary mouse hepatocytes (Fig. 2e–h). Chronic exposure to high insulin level also reduced RIMKLA expressions in mouse hepatocytes (Fig. 2g, h). RIMKLA protein is present both in cytosol and nucleus in human and mouse hepatocytes (Supplementary Fig. 1d–f). Construction and verification of RIMKLA-expressing adenoviruses (Ad) or adeno-associated viruses (AAV) were shown in Supplementary Fig. 1g–i. RIMKLA overexpression suppressed glucose production (Fig. 2i) and alleviated FFA-induced lipid deposition in human and mouse hepatocytes (Fig. 2j–l). Overall, these findings suggested a potential role of RIMKLA in regulating hepatic glucose and lipid metabolism.

### Hepatic RIMKLA overexpression ameliorates hyperglycemia and steatosis in obese mice

**Fig. 3:**
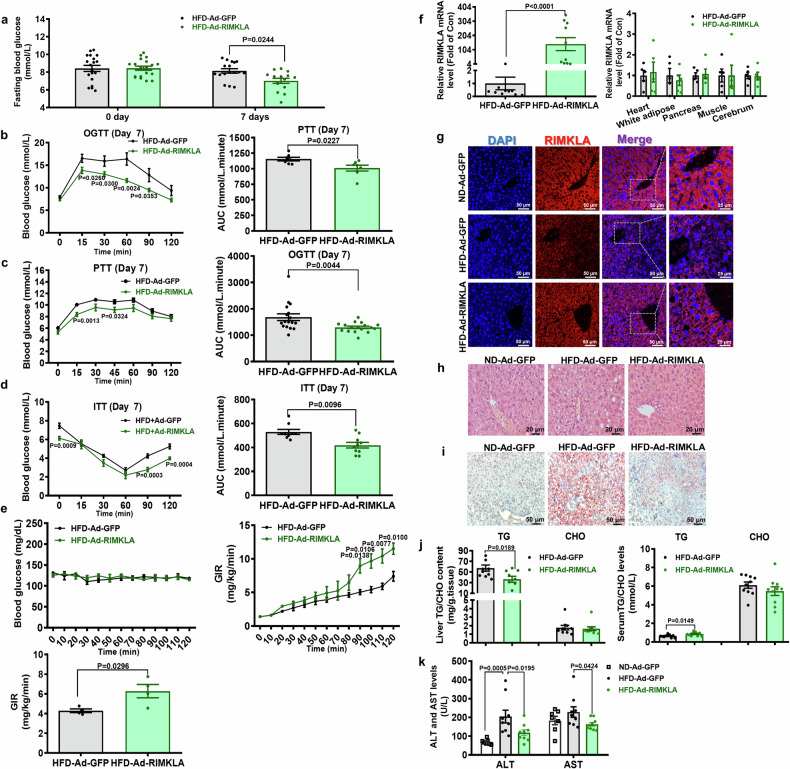
Hepatic RIMKLA overexpression improves dysregulated glucose and lipid metabolism in HFD mice. **a**–**e** Fasting blood glucose, OGTT, PTT, ITT and hyperinsulinemic-euglycemic clamp assays were performed on HFD mice after injection of Ad-GFP or Ad-RIMKLA for 7 days. Fasting blood glucose level was referred to the blood glucose level at 0 min in OGTT. *n* = 7-19 for different tests (**a**–**d**). The analytic data shown in left panels, and areas under curve (AUC) data in right panels for (**b**–**d**). For hyperinsulinemic-euglycemic clamp assay, blood glucose levels were shown in left panel, and glucose infusion rate (GIR) in the right and lower panels (**e**). *n* = 4. **f** Ad-RIMKLA injection on RIMKLA mRNA level in main metabolic tissues of HFD mice. *n* = 10–11 for livers (left) and *n* = 5 for other tissues (right). **g** Ad-RIMKLA injection increased RIMKLA protein level in HFD mouse livers using immunofluorescent staining assays. The shown images were the representatives of 3 independent mouse livers. Blue, DAPI; Red, RIMKLA; Purple, Merge. Scale bar: 50 μm or 25 μm. **h** Representative liver histology images of three groups of mice by Hematoxylin-Eosin staining. Scale bar: 20 µm. **i** Representative Oil Red O staining images of livers in three groups of mice. Scale bar: 50 μm. **j** Hepatic RIMKLA overexpression on hepatic (left panel) and serum (right panel) TG and CHO levels in HFD mice. *n* = 10. **k** Hepatic RIMKLA overexpression reduced serum ALT and AST activities of HFD mice. *n* = 7–9. HFD: mice fed on high-fat diet for 3 months. All data were represented as mean ± SEM. Statistical *P* values were marked in each panel. The data for (**a**, **f**, **j**) and AUC of (**b**–**d**), the average of GIR (**e**, lower panel) were calculated using unpaired t-test, the data for left panels of (**b**–**e**) were analyzed by two-way ANOVA with Tukey’s multiple comparisons tests. Ordinary one-way ANOVA with Bonferroni’s multiple comparisons test was used to analyze the data for (**k**)

To evaluate the roles of hepatic RIMKLA on glucose and lipid metabolism, mice fed on HFD for 3 months were transduced with Ad-green fluorescence protein (GFP) or Ad-RIMKLA, respectively. Glucose tolerance test (OGTT), pyruvate tolerance test (PTT), and insulin tolerance test (ITT) were performed at day 7 post viral injection using different sets of animals. Ad-RIMKLA-treated mice exhibited the significant improvement of fasting glucose level, glucose intolerance, hepatic glucose production, and insulin resistance when compared with control mice (Fig. 3a–d). Hyperinsulinemic-euglycemic clamp assays confirmed the significant increase in insulin sensitivity after Ad-RIMKLA infection (Fig. 3e). mRNA quantitative analyses and immunofluorescent staining indicated that Ad-RIMKLA injection specifically increased RIMKLA expression in livers but not in other main metabolic tissues or cerebrum (Fig. 3f). Both cytoplasmic and nuclear RIMKLA expression were increased in mouse livers after Ad-RIMKLA injection (Fig. 3g and Supplementary Fig. 1j). Hematoxylin-Eosin and Oil Red O staining analyses indicted that RIMKLA overexpression alleviated liver lipid accumulation (Fig. 3h, i). Quantitative assays confirmed that hepatic triglyceride (TG) content was significantly reduced while serum TG level was slightly increased after RIMKLA overexpression (Fig. 3j). In contrast, hepatic and serum cholesterol (CHO) levels remained unchanged after RIMKLA overexpression (Fig. 3j). Moreover, hepatic RIMKLA overexpression decreased serum alanine transaminase (ALT) and aspartate transaminase (AST) activities (Fig. 3k). Mice fed on HFD for 6 months were also used to further evaluate the roles of RIMKLA in NAFLD. There was no difference in OGTT between the two groups before adenovirus injection, and Ad-RIMKLA injection significantly ameliorated hepatic glucose production and glucose intolerance (Supplementary Fig. 2a–c). Another set of mice was used to evaluate the effect of RIMKLA overexpression on insulin sensitivity (Supplementary Fig. 2d). RIMKLA injection improved insulin resistance in these mice (Supplementary Fig. 2e). Moreover, hepatic RIMKLA overexpression reduced hepatic and serum TG levels, and decreased hepatic CHO levels (Supplementary Fig. 2f). Furthermore, Hematoxylin-Eosin and Oil Red O staining analyses indicated that RIMKLA overexpression alleviated hepatic lipid accumulation in these mice (Supplementary Fig. 2g, h).

In consideration of the relative short overexpression time of adenoviral transduction in vivo (generally 1–2 weeks), the long-term effects of hepatic RIMKLA overexpression using AAV subtype 8 (AAV8-GFP versus AAV8-RIMKLA) on glucose/lipid metabolism were further evaluated in db/db mice. No significant improvement on glucose metabolism was observed before 10 weeks post AAV injection (Supplementary Fig. 3a–c). Significant improvements on insulin sensitivity, glucose intolerance, and hepatic glucose production as well as fasting hyperglycemia were observed after 10 weeks post AAV8-RIMKLA injection (Supplementary Fig. 3d–g). AAV8-RIMKLA injection had little effect on body weight over time (Supplementary Fig. 3g, right panel). Magnetic resonance imaging (MRI) analyses revealed that AAV8-RIMKLA injection significantly reduced hepatic fat content and total fat volume in db/db mice (Supplementary Fig. 3h). Hepatic and serum TG content but not CHO level was reduced after RIMKLA overexpression (Supplementary Fig. 3i, j). Overall, acute or chronic hepatic RIMKLA overexpression markedly corrected the dysregulated glucose and lipid metabolism in obese mice.

### RIMKLA inhibits gluconeogenic and lipogenic/lipid-uptake gene expressions in mouse livers and cultured hepatocytes

**Fig. 4:**
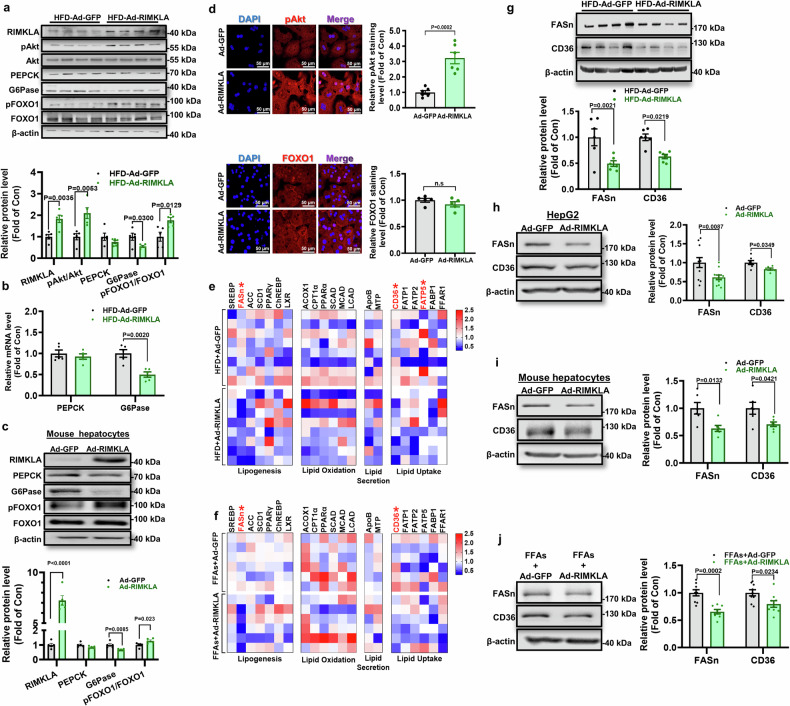
RIMKLA overexpression represses gluconeogenic and lipogenic/lipid-uptake gene expressions in HFD mouse livers and cultured hepatocytes. **a** RIMKLA overexpression on protein levels of glucose and lipid metabolic genes in HFD mouse livers. *n* = 5. **b** RIMKLA overexpression on mRNA levels of gluconeogenic genes in HFD mouse livers. *n* = 5. **c** RIMKLA overexpression on gluconeogenic gene expression in mouse primary hepatocytes. Assays were performed after Ad-GFP or Ad-RIMKLA infection for 24 h. *n* = 5. **d** RIMKLA overexpression increased nuclear distribution of pAkt (upper panel), and induced FOXO1 (lower panel) nuclear exclusion in mouse hepatocyte. The representative immunofluorescent staining of pAkt/FOXO1 was presented in the left panel, and quantitative data of fluorescence intensity was shown in the right panel. *n* = 5–6. Blue, DAPI; Red, pAkt/FOXO1; Purple, Merge. Scale bar, 50 μm. **e**, **f** RIMKLA overexpression on lipid metabolic gene mRNAs in HFD mouse livers (**e**) and cultured mouse hepatocytes (**f**). Hepatocytes were infected with Ad-RIMKLA in the presence of free fatty acids (0.1 mM oleic acid+0.2 mM palmitic acid) for 24 h. *n* = 6–8. **g** RIMKLA overexpression reduced the protein levels of FASn and CD36 in HFD mouse livers. *n* = 6. **h**, **i** RIMKLA overexpression reduced the protein levels of FASn and CD36 in HepG2 cells (**h**) and mouse hepatocytes (**i**). *n* = 6–11. **j** RIMKLA overexpression reduced the protein levels of FASn and CD36 in the presence of FFAs in mouse hepatocytes. FFAs, free fatty acids (0.1 mM oleic acid+0.2 mM palmitic acid). *n* = 8–9. All data were represented as mean ± SEM. Statistical *P* values were marked in each panel. All *P* values were determined using student’s t-test

In diabetic mouse livers, Ad- or AAV8-RIMKLA injection increased RIMKLA protein level by about 2 folds (Fig. 4a and Supplementary Fig. 4a, b). RIMKLA overexpression increased protein kinase B (Akt) phosphorylation with decreased mRNA and protein levels of glucose 6 phosphatase (G6Pase) in mouse livers (Fig. 4a, b and Supplementary Fig. 4a, b). In support, RIMKLA overexpression increased nuclear distribution of phosphorylated Akt (pAkt), and promoted forkhead box protein O1 (FOXO1) nuclear exclusion, and reduced G6Pase expression in mouse hepatocytes (Fig. 4c, d) and HepG2 cells (Supplementary Fig. 5a, b), which supported the observations that RIMKLA suppressed glucose production in hepatocytes (Fig. 2i). To investigate the mechanism of RIMKLA in lipid metabolism, we evaluated the effects of RIMKLA overexpression on the mRNA levels of key lipid metabolic genes involved in various processes including lipogenesis [sterol regulatory element-binding protein 1(SREBP1), fatty acid synthase (FASn), acetyl-coA carboxylase (ACC), stearoyl-coA desaturase-1 (SCD1), peroxisome proliferator-activated receptor γ (PPARγ), carbohydrate response element binding protein (ChREBP), liver X receptor (LXR)], lipid oxidation [acyl-coA oxidase 1 (ACOX1), carnitine palmitoyltransferase 1α (CPT1α), peroxisome proliferator-activated receptor α (PPARα), short-chain acyl-coA dehydrogenase (SCAD), medium-chain acyl-coA dehydrogenase (MCAD), long-chain acyl-coA dehydrogenase (LCAD)], lipid secretion [apolipoprotein B (ApoB), microsomal triglyceride transfer protein (MTP)], and lipid uptake [CD36, fatty acid transport protein 1 (FATP1), FATP2, FATP5, fatty acid-binding protein 1 (FABP1), free fatty acid receptor 1 (FFAR1)], as conferred to many studies.^30,31^ Interestingly, the mRNA levels of FASn, one of the key lipogenic genes, and CD36, one of the key fatty acid transporters, were consistently reduced in both HFD mouse livers and FFA-induced hepatocytes after RIMKLA overexpression (Fig. 4e, f). Although RIMKLA overexpression reduced the mRNA level of FATP5 in HFD-induced mouse livers, no consistent effect was observed in cultured hepatocytes (Fig. 4e, f). RIMKLA overexpression reduced FASn and CD36 protein levels in obese mouse livers, human HepG2 cells and mouse hepatocytes (Fig. 4g–i and Supplementary Fig. 4a, b). Although RIMKLA injection had little effect on OGTT and PTT in normal mice, it significantly decreased the protein expression levels of FASn and CD36 in the livers (Supplementary Fig. 6a–d). Moreover, RIMKLA overexpression also reduced FASn and CD36 protein levels in the presence of FFAs in mouse hepatocytes (Fig. 4j), supporting that RIMKLA overexpression reduced FFA-promoted lipid deposition (Fig. 2j–l). Collectively, RIMKLA overexpression ameliorated hepatic lipid deposition by repressing de novo lipogenesis and lipid uptake, and suppressed gluconeogenesis by inhibiting FOXO1.

### RIMKLA interacts with and activates BHMT1 by phosphorylating Thr45

**Fig. 5:**
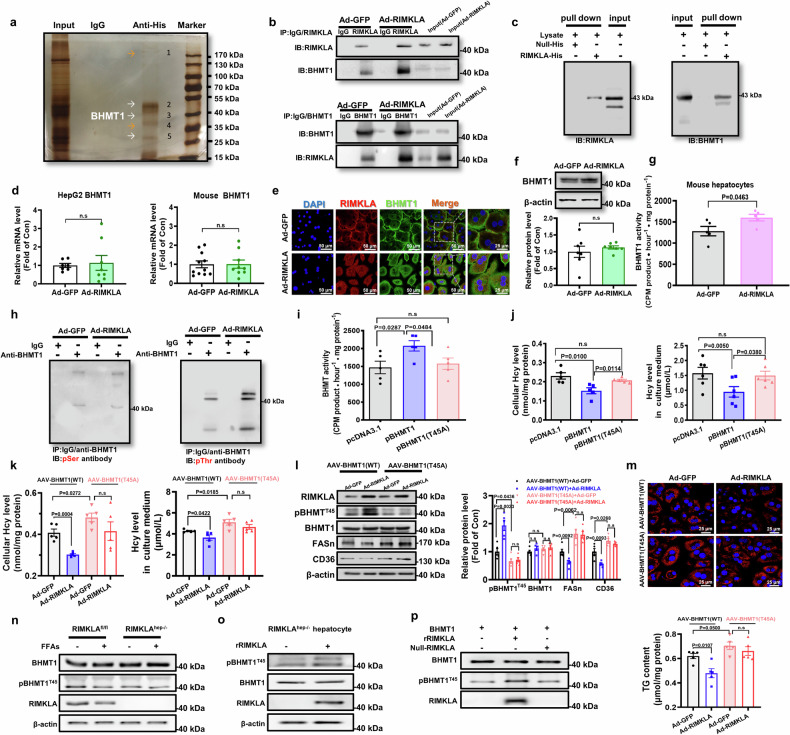
RIMKLA interacts with and activates BHMT1 by phosphorylating Thr45 site in hepatocytes. **a** Co-IP assay and MS analyses identified BHMT1 as one of RIMKLA-interacted molecules. Hepatocytes were infected with Ad-RIMKLA for 24 h, and then the lysate was subjected to Co-IP. The gel was visualized using silver nitrate staining, and the indicated bands were analyzed by MS. IP: IgG or anti-His (Ad-RIMKLA tagged with 6 ×His). **b** Confirmation of RIMKLA-BHMT1 interaction using Co-IP assays in mouse hepatocytes. Hepatocytes were infected with Ad-GFP or Ad-RIMKLA for 24 h before subjecting for Co-IP assays. The images were the representatives of 3 independent experiments. **c** Confirmation of RIMKLA-BHMT1 interaction using purified RIMKLA-His fusion protein and BHMT1 protein in mouse hepatocytes. The images were the representatives of 3 independent experiments. **d** RIMKLA overexpression on BHMT1 mRNA level in HepG2 cells (left panel) and mouse hepatocytes (right panel). *n* = 7–11. **e**, **f** BHMT1 protein level was determined by immunofluorescent staining (**e**) and western blotting assays (**f**) after RIMKLA overexpression. *n* = 7 for western blotting assays. For (**e**), DAPI (Blue), RIMKLA (Red), BHMT1 (Green), Merge (Yellow). Scale bar: 50 µm or 25 µm. **g** RIMKLA overexpression increased cellular BHTM1 activity in mouse hepatocytes. BHMT1 activity was analyzed as described in methodology. *n* = 5. **h** RIMKLA stimulated several PTM types in BHMT1 protein in mouse hepatocytes. Hepatocytes were infected with Ad-RIMKLA for 24 h, and then subjected to IP assays using anti-BHMT1 antibodies. Blotting assays were performed using antibodies against pSer and pThr. The images were the representatives of 3 independent experiments. **i** Mutation of T45A inactivated BHMT1 in mouse hepatocytes. *n* = 5. **j** Mutation of T45A abolished BHMT1’s activity for clearing Hcy in mouse hepatocytes. Intracellular Hcy data shown in left panel, and extracellular Hcy data in right panel. *n* = 5–6. **k** Mutation of T45A abolished RIMKLA’s repression on Hcy levels in mouse hepatocytes. Mouse hepatocytes were infected with wild type or mutant AAV8-BHMT1 for 48 h, followed by Ad-GFP or RIMKLA infection for 24 h. *n* = 5. **l** Mutation of T45A abolished RIMKLA’s repression on FASn and CD36 expressions in mouse hepatocytes. Cells were treated as above. *n* = 5. **m** Mutation of T45A blunted RIMKLA’s repression on FFAs-induced lipid deposition in mouse hepatocytes. Representative images for lipid staining were shown in upper panel, quantified cellular TG content were shown in lower panel. Cells were treated as above in the presence of fatty acids (0.1 mM oleic acid+0.2 mM palmitic acid). *n* = 5. Scale bar: 25 µm. **n** FFAs treatment reduced BHMT1 phosphorylation in mouse hepatocytes. Wild type and RIMKLA-deficient hepatocytes were treated with FFAs for 24 h. The images were the representatives of 3 independent experiments. **o** Addition of recombinant RIMKLA (rRIMKLA) induced BHMT1 phosphorylation in RIMKLA-deficient mouse hepatocytes. The images were the representatives of 3 independent experiments. **p** rRIMKLA tagged with 6×His was purified as described in Materials and Methods. BHMT1 protein was immunoprecipitated by anti-BHMT1 antibodies using cultured hepatocytes. Then, immunoprecipitated BHMT1 protein was incubated in the absence or presence of rRIMKLA (50 ng/mL) at 37 °C for 2 h. After incubation, RIMKLA, phospho-BHMT1^T45^ and BHMT1 protein levels were detected by western blotting. The images were the representatives of 3 independent experiments. All data were represented as mean ± SEM. Statistical *P* values were marked in each panel. *P* values for the left panel of (**d**) were determined by Mann–Whitney test, for the right panel of (**d**) and (**f**, **g**) were analyzed using t-test, for (**i**–**m**) using one-way ANOVA followed by Bonferroni’s post hoc test

**Fig. 6:**
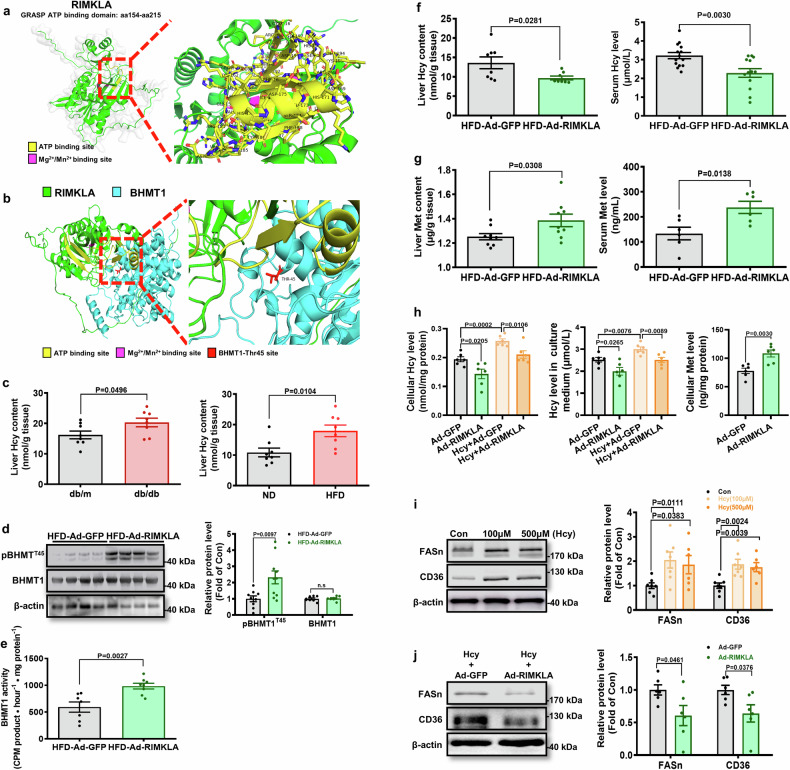
RIMKLA overexpression enhances Hcy clearing in mice and cultured hepatocytes. **a** Prediction of kinase domain in RIMKLA. **b** Molecular docking between RIMKLA and BHMT1. The interaction affinities between the RIMKLA and BHMT1 was analyzed by ZDOCK (https://zdock.wenglab.org/). The PDB formats of the RIMKLA and BHMT1 structural domains were downloaded from the Protein Data Bank PDB database (http://www.rcsb.org/). **c** Hepatic Hcy content was increased in diabetic mice. *n* = 8. **d** RIMKLA overexpression elevated phosphorylated BHMT1 level in HFD mouse livers. *n* = 8–10. **e** RIMKLA overexpression increased hepatic BHMT1 activity in HFD mice. *n* = 7–8. **f** RIMKLA overexpression decreased hepatic (left panel) and serum Hcy (right panel) content in HFD mice. *n* = 8–14. **g** RIMKLA overexpression increased hepatic (left panel) and serum (right panel) Met content in HFD mice. *n* = 6–9. **h** RIMKLA overexpression reduced Hcy levels in the absence or presence of Hcy treatment (left and middle panels). RIMKLA overexpression increased cellular Met level in the absence of Hcy (right panel). *n* = 6. **i** Hcy upregulated FASn and CD36 expressions in mouse hepatocytes. Mouse hepatocytes were treated with different concentrations of Hcy for 24 h before detection. *n* = 6–7. **j** RIMKLA overexpression reduced FASn and CD36 expression in the presence of 100 μM Hcy. *n* = 6. All data were represented as mean ± SEM. Statistical *P* values were marked in each panel. *P* values for (**c**–**g**, **j**) and right panel of (**h**) were analyzed by student’s t-test, for left panels of (**h**) and (**i**) were determined by one-way ANOVA with Bonferroni’s post hoc test

To further pinpoint the mechanism(s) of RIMKLA’s repression on FASn and CD36 expressions, RIMKLA containing 6×His-tag was overexpressed in mouse hepatocytes, and then Co-Immunoprecipitation (Co-IP) was performed using IgG or anti-His antibodies. The Co-IP products were separated by PAGE, visualized by silver staining, and then several indicated protein bands were analyzed by mass spectrometry (MS) (Fig. 5a). Unique peptides for BHMT1 were detected in three of five indicated bands with the strongest signal in band 3, which matched the molecular weight of BHMT1 (Fig. 5a and Supplementary Table 2). Because it had been previously reported that BHMT1 played important roles in regulating lipid metabolism,^20–23^ whether RIMKLA interacted with it to regulate lipid metabolism was further probed. Cross-Co-IP and western blot analyses revealed that RIMKLA interacted with BHMT1, and their interaction was augmented after RIMKLA overexpression in mouse hepatocytes (Fig. 5b). His-tag pulldown experiments further confirmed the direct interaction between RIMKLA and BHMT1 (Fig. 5c). RIMKLA overexpression had little effect on BHMT1 mRNA and protein levels in mouse and human hepatocytes (Fig. 5d–f and Supplementary Fig. 7a). Notably, RIMKLA overexpression increased the Hcy-Met converting activity of BHMT1 in mouse and human hepatocytes (Fig. 5g and Supplementary Fig. 7b). Because RIMKLA overexpression increased BHMT1 activity without affecting its cellular protein level, we speculated that RIMKLA may activate it by inducing protein modification(s). To address this issue, the potential modification site(s) in BHMT1 protein was predicted using softwares PhosphoSitePlus v6.6.0.2 (www.phosphosite.org) and dbPTM (www.dbPTM.org). As a result, threonine (Thr) 45 and serine (Ser) 79 sites were the common modification sites predicted to be present in BHMT1 protein using different methods (Supplementary Fig. 8a). Then, whether RIMKLA induced BHMT1 protein phosphorylation was determined. Ad-RIMKLA- and Ad-GFP-treated hepatocytes were immunoprecipitated with anti-BHMT1 antibodies, and then immunoblotted using phosphorylated serine (pSer) antibodies and phosphorylated threonine (pThr) antibodies, respectively. As a result, RIMKLA overexpression increased total Thr phosphorylation without affecting Ser phosphorylation on BHMT1 protein in hepatocytes (Fig. 5h). To further explore the distinct modification site(s) on BHMT1 induced by RIMKLA overexpression, Co-IP-MS assays were performed. Potential BHMT1 modification sites identified by MS were shown in Supplementary Table 3. Notably, a peptide containing phosphorylated Thr45 was detected in RIMKLA-overexpressed cells but not in control cells (Supplementary Fig. 8b, c and Supplementary Table 3). In contrast, no other phosphorylation site(s) or acetylation site(s) were identified by MS (Supplementary Fig. 8b, c and Supplementary Table 3). Thr45 is one of the predicted potential modification sites, and RIMKLA overexpression increased total Thr phosphorylation of BHMT1 (Supplementary Fig. 8a and Fig. 5h). Thr45 site is highly conserved across species, and predicted to be important for BHMT1’s activity using software Uniprot (www.uniprot.org) (Supplementary Fig. 9a/b). Mutation of Thr45 to alanine (T45A) inactivated BHMT1’s enzymatic activity (Fig. 5i, j). In addition, the inhibitory effects of BHMT1 on lipid deposition, and FASn and CD36 expressions were also abolished after T45A mutation (Supplementary Fig. 9c, d). Furthermore, RIMKLB overexpression had little effect on Thr45 phosphorylation of BHMT1 in HepG2 cells (Supplementary Fig. 9e). Construction and verification of AAVs that express wild type and mutant BHMT1 genes were shown in Supplementary Fig. 9f. Western blotting analyses confirmed that RIMKLA promoted BHMT1 phosphorylation at Thr45 in cells that overexpressed wild type BHMT1. In cells infected with mutant AAV-BHMT1, phosphorylated BHMT1 level was lower than those infected with wild-type AAV-BHMT1, and RIMKLA failed to increase it. Importantly, T45A mutation blunted RIMKLA’s repression on intracellular and extracellular Hcy levels, FASn and CD36 expressions, and lipid deposition in cultured hepatocytes (Fig. 5k–m). Treatment with FFAs reduced RIMKLA expression and BHMT1 phosphorylation without affecting BHMT1 expression in wild-type mouse hepatocytes. In RIMKLA-deficient hepatocytes, BHMT1 phosphorylation was reduced when compared with wild type hepatocytes, but FFAs failed to further inhibit it (Fig. 5n). To further validate whether RIMKLA directly phosphorylated BHMT1, immunoprecipitated BHMT1 protein from hepatocytes was incubated with or without purified recombinant RIMKLA (rRIMKLA), the purity of which was about 95% (Supplementary Fig. 10a). Addition of rRIMKLA induced BHMT1 phosphorylation in lysate of RIMKLA-deficient hepatocytes (Fig. 5o). The results clearly indicated that RIMKLA directly phosphorylated BHMT1 at Thr45 (Fig. 5p). In addition, the phosphokinase kinetic parameters of RIMKLA on BHMT1 were detected by incubating rRIMKLA with recombinant BHMT1 (rBHMT1) in vitro, and the maximum reaction rate (V_max_) and Michaelis-Menten constant (K_m_) were found to be 0.6832 μM/min and 1.622 μM, respectively (Supplementary Fig. 10b). Furthermore, domain prediction reveals that there is one GRASP ATP-binding domain covering aa154 to aa215 in RIMKLA (Fig. 6a). Notably, molecular docking prediction using ZDOCK reveals that RIMKLA indeed interacts with BHMT1, and Thr45 of BHMT1 is located at the RIMKLA-BHMT1 interaction interface, very close to the predicted GRASP-ATP domain of RIMKLA (Fig. 6b).

BHMT1 is colocalized with RIMKLA in human and mouse livers (Supplementary Fig. 11a, b). In addition, the levels of phosphorylated BHMT1 (T45) and ratio of pBHMT1/BHMT1 were both reduced in livers of db/db mice (Supplementary Fig. 12a) and HFD mice (data not shown), accompanied with increased hepatic Hcy content (Fig. 6c). Serum Hcy level was increased in patients with diabetes but remained unchanged in diabetic mice (Supplementary Fig. 12b). RIMKLA overexpression increased BHMT1 phosphorylation at Thr45 with little effect on total BHMT1 protein level in HFD mouse livers (Fig. 6d). RIMKLA overexpression increased hepatic BHMT1 activity in HFD mice (Fig. 6e). RIMKLA overexpression reduced Hcy level and increased Met level in both liver and serum of obese mice (Fig. 6f, g and Supplementary Fig. 12c, d). In cultured hepatocytes, RIMKLA overexpression reduced intracellular and extracellular Hcy levels in the absence or presence of Hcy (Fig. 6h). RIMKLA overexpression also increased cellular Met level in hepatocytes (Fig. 6h). In mouse hepatocytes infected with AAV8-BHMT1, RIMKLA overexpression increased the Hcy clearance rate when compared to Ad-GFP. In contrast, in mouse hepatocytes infected with mutant AAV8-BHMT1 T45A, RIMKLA overexpression failed to facilitate the Hcy clearance, leading to a decreased average reaction velocity (Supplementary Fig. 13a, b). RIMKLA overexpression had little effect on the protein levels of MTR and CBS, the other two enzymes for Hcy clearing, in both mouse livers and cultured hepatocytes (Supplementary Fig. 14a–c). Hcy treatment upregulated FASn and CD36 expressions in hepatocytes, but was inhibited by RIMKLA overexpression (Fig. 6i, j). It had been previously reported that Hcy could activate aryl hydrocarbon receptor (AHR) expression to induce CD36 transcription in hepatocytes.^12^ However, RIMKLA overexpression had little effect on AHR mRNA level (Supplementary Fig. 15a, b) in livers and isolated hepatocytes, suggesting that RIMKLA unlikely promoted the transcriptions of FASn and CD36 via AHR.

**Fig. 7:**
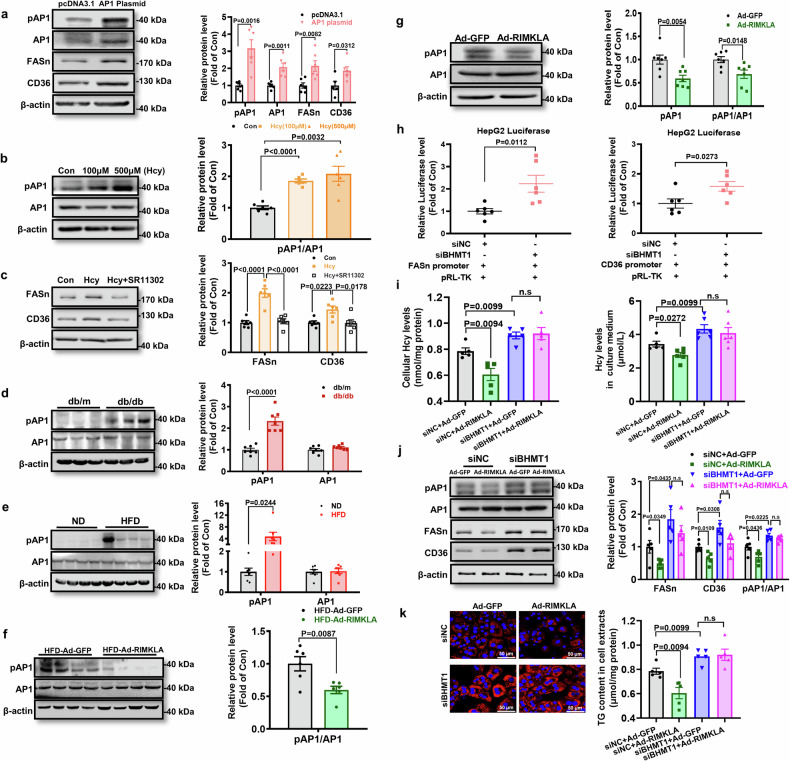
RIMKLA inhibits FASn and CD36 expression by blocking Hcy-induced AP1 activation. **a** AP1 overexpression upregulated FASn and CD36 protein levels in mouse hepatocytes. Cells were transfected with plasmid for 24 h before assays. *n* = 5–6. **b** Hcy activated AP1 in mouse hepatocytes. Cells were treated with various Hcy concentrations (100 µM, 500 µM) for 24 h before assays. *n* = 6. **c** Hcy-induced FASn and CD36 expressions were blocked by AP1 inhibitor in mouse hepatocytes. Cells were treated with 100 μM Hcy in the absence or presence of 20 μM AP1 inhibitor (SR11302) for 24 h before assays. *n* = 6. **d**–**e** AP1 was activated in the livers of obese mice. *n* = 7. **f** RIMKLA overexpression repressed AP1 phosphorylation in mouse livers fed on HFD for 3 months. *n* = 6. **g** RIMKLA overexpression repressed AP1 phosphorylation in cultured mouse hepatocytes. *n* = 7. **h** siRNA silencing of BHMT1 increased the transcriptional activities of both FASn and CD36 promoters. *n* = 6. **i** Silencing of BHMT1 blunted RIMKLA-induced inhibition on Hcy content in mouse hepatocytes. *n* = 5–6. **j** Silencing of BHMT1 inhibited RIMKLA’s repression on AP1 phosphorylation, and FASn and CD36 expressions in mouse hepatocytes. *n* = 5. **k** Silencing of BHMT1 abolished RIMKLA-inhibition on lipid deposition in mouse hepatocytes in the presence of fatty acids (0.1 mM oleic acid+0.2 mM palmitic acid). *n* = 5. Scale bar: 50 µm. All data were represented as mean ± SEM. Statistical *P* values were marked in each panel. *P* values for (**a**, **d**–**g**, **h**) were calculated by student’s t-test, for (**b**, **c**, **i**–**k**) were analyzed using one-way ANOVA followed by Bonferroni’s post hoc analysis

Because RIMKLA consistently promoted the transcriptions of FASn and CD36 genes in mouse and human hepatocytes, we speculated that this was likely achieved through some universal transcription factor(s). So, the potential transcriptional binding sites in FASn and CD36 gene promoter regions of human and mouse were bioinformatically predicted under high sensitivity. As a result, the prediction indicated that activator protein 1 (AP1) was the most frequently occurring and the only universal transcription factor potentially involved in the transcription of the FASn and CD36 genes in both human and mouse (Supplementary Fig. 16a, b). AP1 is a heterodimer, composed of c-Fos and c-Jun subunits.^32^ It had been previously reported that hepatic AP1 activity was increased in diabetic mice, and lentiviral-mediated knockdown of hepatic AP1 alleviated insulin resistance and fatty liver.^33,34^ Then, whether RIMKLA repressed FASn and CD36 expression via the inhibition of AP1 was determined. First, the reporters of mouse FASn and CD36 gene promoters were constructed. We firstly confirmed that RIMKLA overexpression inhibited the transcriptional activities of FASn and CD36 promoters, respectively (Supplementary Fig. 17a). The promoter activities of FASn and CD36 genes were activated by AP1, and further augmented by Hcy. Mutations of the potential AP1 binding sites completely abolished the activation effects of AP1 and Hcy on FASn and CD36 promoter activities (Supplementary Fig. 17b–d). Plasmid-mediated AP1 overexpression upregulated the mRNA and protein levels of FASn and CD36 in hepatocytes (Fig. 7a and Supplementary Fig. 18a). Hcy treatment induced AP1 phosphorylation and FASn/CD36 expressions, and treatment with AP1 inhibitor reduced FASn and CD36 expressions in the absence or presence of Hcy in mouse hepatocytes (Fig. 6i, Fig. 7b, c and Supplementary Fig. 18b). In diabetic mouse livers, AP1 phosphorylation was increased (Fig. 7d, e). These findings revealed that both FASn and CD36 are the target genes of AP1. In mouse livers and cultured hepatocytes, RIMKLA overexpression inhibited AP1 activity (Fig. 7f, g). siRNA inhibition of BHMT1 increased the transcriptional activities of FASn and CD36 promoters in HepG2 cells, respectively (Fig. 7h). In support, siRNA inhibition of BHMT1 increased intracellular and extracellular Hcy levels, elevated AP1 phosphorylation, upregulated FASn and CD36 expressions and promoted lipid deposition in hepatocytes (Fig. 7i–k and Supplementary Fig. 18c). Silencing of BHMT1 also blunted RIMKLA-promoted repression on Hcy level, AP1 phosphorylation, FASn and CD36 expressions, and lipid deposition (Fig. 7i–k). Taken together, RIMKLA phosphorylates BHMT1 at Thr45 to clear Hcy, and inhibit Hcy-induced AP1 activation and upregulation of FASn and CD36, reducing lipid deposition.

### Hepatocyte-specific deletion of RIMKLA aggravates HHcy, hyperglycemia, and steatosis in HFD mice

**Fig. 8:**
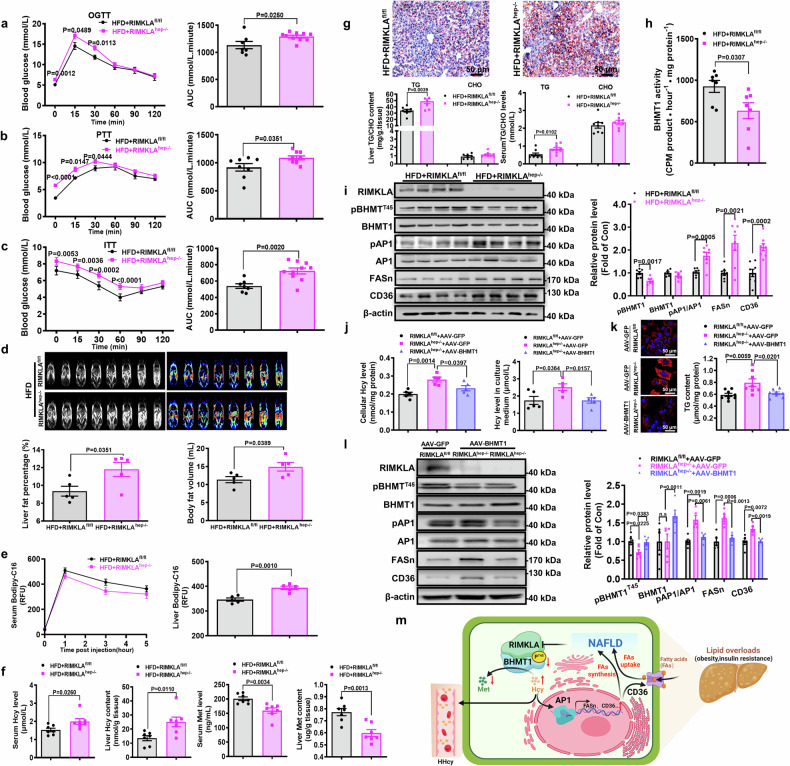
Liver specific knockout of RIMKLA leads to aggravated metabolic disorders. **a**–**c** OGTT, PTT, and ITT were performed on RIMKLA^hep-/-^ and RIMKLA^fl/fl^ mice after feeding on high-fat diet for 8 weeks, 10 weeks, and 12 weeks, respectively. AUC were shown on the right panels. RIMKLA^hep-/-^, Hepatocyte-specific RIMKLA-knockout mice; RIMKLA^fl/fl^, RIMKLA flox/flox mice. *n* = 7–10. **d** MRI analyses of hepatic and body fat distribution when RIMKLA^fl/fl^ and RIMKLA^hep-/-^ mice were fed on HFD for 10 weeks. Representative images were shown on the upper panels, and quantitative data shown on the lower panels. *n* = 5. **e** Fatty acid uptake test was performed using lipid dye BODIPY-C16 (Invitrogen) methodology. Serum fluorescence was measured at 0, 1, 3, 5 h. Liver fluorescence was analyzed at 5 h after Bodipy-C16 injection (i.p.). *n* = 5. **f** Hcy and Met levels in serum and liver after feeding on HFD for 12 weeks. *n* = 7. **g** RIMKLA^hep-/-^ mice exhibited increased lipid deposition as determined by Oil Red O staining livers (upper panel; Scale bar, 50 µm) and lipid determination (lower left panel). Serum TG but not CHO levels were increased in RIMKLA^hep-/-^ mice than in control mice (lower right panel). *n* = 8–9. **h** RIMKLA deficiency impaired BHMT1 enzymatic activity in mouse livers. *n* = 7. **i** Changes of BHMT1 phosphorylation and metabolic protein levels in RIMKLA^hep-/-^ mouse livers. *n* = 7–8. **j** RIMKLA deficiency increased intracellular (left panel) and extracellular (right panel) Hcy levels, but was rescued by BHMT1 overexpression in cultured hepatocytes. *n* = 5. **k** BHMT1 overexpression reversed increased lipid deposition induced by RIMKLA deficiency in the presence of FFAs in cultured hepatocytes. Cells were infected with indicated viruses in the presence of 0.1 mM oleic acid+0.2 mM palmitic acid for 48 h. Representative lipid staining images were shown in left panel (DAPI, blue; Lipid, red; Scale bar, 50 µm), and quantitative data shown in right panel. *n* = 8. **l** Decreased expression of phospho-BHMT1 (T45) and increased expressions of phosphorylated AP1, CD36 and FASn caused by RIMKLA deletion were reversed by BHMT1 overexpression in hepatocytes. *n* = 5–6. All data were represented as mean ± SEM. Statistical *P* values were marked in each panel. Statistical *P* values were analyzed using two-way ANOVA followed by Tukey’s tests for left panels of (**a**–**c**, **e**), student’s t-test for the right panels of (**a**–**c**, **e**) and (**d**, **f**, **g**–**i**), one-way ANOVA with Bonferroni’s post hoc tests for (**j**–**l**). **m** Proposed model of RIMKLA in regulating Hcy and lipid metabolism (created with BioRender.com). RIMKLA activates BHMT1 activity by phosphorylating at Thr45 site, and clears Hcy by converting it to Met. Under obese condition, hyperlipidemia and hyperinsulinemia repress RIMKLA to impair BHMT1 activity and elevate Hcy level, which activates AP1 to induce the transcriptions of FASn and CD36, increasing de novo lipid synthesis and uptake to promote hepatic lipid deposition. Under obese or insulin-resistant conditions, the changes in protein expression or activity, molecules and biological processes were marked in red arrows. AP1 activator protein 1, BHMT1 betaine-homocysteine S-methyltransferase 1, CD36 cluster of differentiation 36, FAs fatty acids; Hcy homocysteine, HHcy hyperhomocysteinemia, Met methionine, NAFLD non-alcoholic fatty liver disease, pT45 phosphorylation at Thr45 site, RIMKLA ribosomal modification protein rimK-like family member A

To further confirm the roles of RIMKLA in regulating Hcy and glucose/lipid metabolism, mice with hepatocyte-specific knockout of RIMKLA (RIMKLA^hep-/-^) were generated. RIMKLA^fl/fl^ mice served as the controls (Supplementary Fig. 19a). The characterizations of RIMKLA^hep-/-^ mice were shown in Supplementary Fig. 19b, c. RIMKLA^hep-/-^ mice fed on normal diet showed similar metabolic phenotype except for the slightly higher blood glucose levels at the time point of 15 min in OGTT (8 weeks and 12 weeks) and mildly increased body fat volume as RIMKLA^fl/fl^ mice (Supplementary Fig. 20a–f). So, another set of RIMKLA^fl/fl^ and RIMKLA^hep-/-^ mice were subjected to HFD feeding at the age of 8 weeks. After HFD feeding for 8-12 weeks, RIMKLA^hep-/-^ mice exhibited more severe glucose intolerance, hepatic glucose production, and insulin resistance than control mice (Fig. 8a–c). MRI analyses revealed that RIMKLA^hep-/-^ mice had more hepatic fat content and total fat volume when compared with control mice (Fig. 8d). Moreover, fatty acid uptake experiments using BODIPY-FL-C_16_ indicated that RIMKLA^hep-/-^ mice had higher lipid uptake rate than control mice (Fig. 8e). Deletion of RIMKLA in hepatocyte resulted in increased Hcy levels, and decreased Met levels in both serum and liver (Fig. 8f). Oil Red O staining and TG/CHO quantification assays revealed that more severe hepatic lipid deposition happened in RIMKLA^hep-/-^ mice while liver and serum CHO levels remained unchanged (Fig. 8g). BHMT1 phosphorylation and enzymatic activity were reduced while its protein level remained unchanged in RIMKLA^hep-/-^ mouse livers (Fig. 8h, i). Phosphorylated AP1, and FASn and CD36 expressions were increased in RIMKLA-knockout mouse livers than in control mouse livers (Fig. 8i). In cultured RIMKLA^hep-/-^ mouse hepatocytes, Hcy levels, AP1 phosphorylation, FASn and CD36 expressions, and lipid deposition were increased than in control mouse hepatocytes, but were rescued by BHMT1 overexpression (Fig. 8j–l). In RIMKLA-deficient hepatocytes, phosphorylated BHMT1 was reduced while total BHMT1 protein remained unchanged (Fig. 8l).

To further validate the role of BHMT1-T45 in Hcy and lipid metabolism in vivo, wild type (WT) and mutant BHMT1 were overexpressed in normal mice followed by HFD feeding. Overexpression of WT BHMT1 significantly reduced fat volume, lipid deposition, and hepatic TG and Hcy content (Supplementary Fig. 21a–h). Notably, overexpression of BHMT1-T45A failed to reduce Hcy and TG content in mouse livers (Supplementary Fig. 21g, e). In support, overexpression of WT BHMT1 but not mutant BHMT1 reduced AP1 phosphorylation, and FASn and CD36 expressions in mouse livers (Supplementary Fig. 21i). Moreover, RIMKLA^hep-/-^ mice were also subjected to Hcy feeding. As a result, RIMKLA^hep-/-^ mice exhibited more severe hepatic lipid deposition, elevated Hcy levels in liver and serum, and increased AP1 phosphorylation and FASn/CD36 expressions in the livers after Hcy feeding (1.8 g/L DL-Hcy in drinking water) for 8 weeks (Supplementary Fig. 22a–d).

## Discussion

Hcy is an intermediate of Met metabolism. Dietary-derived amino acid Met is catalyzed to Hcy via processes controlled by several enzymes including by S-adenosine methionine synthetase, methyltransferase (phosphatidylethanolamine N-methyltransferase or guanidine-acetate N-methyltransferase) and S-adenosine homocysteine hydrolase (SAHH).^5^ HHcy results from the imbalance between increased production and decreased clearing of Hcy. Elevated serum Hcy levels is highly correlated with the progression of NAFLD^10,35^ and type 2 diabetes mellitus.^36,37^ Patients with diabetes with HHcy exhibited higher levels of Hemoglobin A1c (HbA1c), TC, TG and insulin resistance than those with diabetes alone.^36^ HHcy combined with metabolic disorders increases overall and cardiovascular mortality rates.^38,39^ So far, although it had been widely accepted that HHcy is highly associated with many disorders, its underlying mechanism(s) is still poorly understood. Moreover, the deleterious effect(s) and mechanism(s) of Hcy on glucose and lipid metabolism also needed further exploration. RIMK family contains two members, designated as RIMKLA and RIMKLB, respectively. The current study identified RIMKLA as a novel important regulator of Hcy and glucose/lipid metabolism. Furthermore, we provided strong evidence that RIMKLA is a new protein kinase. RIMKLA interacts with and phosphorylates BHMT1 at Thr45 site to activate it, clearing Hcy and repressing Hcy-induced upregulation of FASn and CD36 via the inhibition of AP1 to inhibit hepatic de novo lipid synthesis and uptake. Clearly, RIMKLA-BHMT1 axis represents a new regulation mode for the homeostasis of Hcy and lipid. Inhibition of RIMKLA to impair BHMT1 activity is a novel pathogenic mechanism for HHcy. Moreover, these findings further verified the accuracy of GIC in predicting and screening important genes based on database search or large scale sequencing.^27^ RIMKLB shares 65% sequence identity with RIMKLA and is mainly expressed in the CNS, placenta and testis.^28^ RIMKLB is responsible to catalyze the ATP-dependent synthesis of N-Acetyl-Aspartyl-Glutamate (NAAG) and β-citrylglutamate (BCG), the latter of which is a structural analog of NAAG.^28^ Except for regulating neuroexcitability, RIMKLB was also reported to be involved in spermatogenesis.^40^ Although the role of RIMKLB in glucose and lipid metabolism remains unknown, it has a lower GIC prediction score than RIMKLA. In addition, the mRNA expression of RIMKLB remained unchanged in the livers of HFD mice. Furthermore, RIMKLB overexpression had little effect on the protein expression levels of BHMT1 and the phosphorylation of BHMT1 at Thr45.

BHMT1 is highly expressed in the liver and kidney.^41^ Previous study had revealed that BHMT1 is responsible for clearing more than 25% of hepatic Hcy.^42,43^ Although previous studies had revealed the important roles of BHMT1 in maintaining Hcy homeostasis, they were focused on its expression, and whether its enzymatic activity could be modulated by protein modification(s) remained unknown.^20–23^ In the current study, we observed that RIMKLA overexpression increased hepatic BHMT1 activity in HFD mice and both mouse and human hepatocytes. In addition, the phosphokinase kinetic parameters of RIMKLA on BHMT1 were calculated in vitro. Meanwhile, the enzyme activity of BHMT1 significantly decreased in RIMKLA^hep-/-^ mice livers fed on HFD. Furthermore, we provided the experimental evidence that RIMKLA can induce Thr phosphorylation on BHMT1 protein in hepatocytes. Particularly, we provided the convincing evidence that RIMKLA directly phosphorylates BHMT1 at Thr45 site. Once the T45A mutant of BHMT1 was introduced, RIMKLA overexpression failed to facilitate the clearance of Hcy. This indicated that Thr45 site of BHMT1 is vital for its Hcy-Met converting activity in hepatocytes. To our knowledge, this is the first report revealing the enzymatic activity of BHMT1 can be regulated by phosphorylation, which greatly expanded the understanding on the regulation of its enzymatic activity and in-depth roles in regulating Hcy metabolism.

Teng et al. reported that BHMT1 heterozygotes exhibited the decrease of BHMT1 activity, but no change of plasma Hcy, and hepatic TG concentration, and BHMT^-/-^ mice displayed increased hepatic/serum Hcy levels, and severe liver injury and lipid deposition without HFD challenge.^22^ Although BHMT1 expression was decreased in obese mouse livers, hepatic RIMKLA overexpression phosphorylated its T45 site to ameliorate HHcy and steatosis without affecting the protein level, suggesting that modulation of BHMT1 activity was sufficient to attenuate HHcy and hepatic lipid deposition. Under obese condition, hyperlipidemia and hyperinsulinemia repress RIMKLA expression to inhibit BHMT1 activity, finally causing HHcy. At present, activation of MTR, which also converts Hcy to Met using folate as methyl donor, by supplementation of folate is the only strategy for clinically treating HHcy. In the present study, RIMKLA overexpression had little effect on the protein expression level of MTR in the livers of HFD mice and mouse hepatocytes, suggesting that the conversion of Hcy to Met induced by RIMKLA was primarily mediated by BHMT1. Given that HHcy is an important risk factor for many diseases, additional strategies for treating it should be developed. Our findings suggested that activation of hepatic RIMKLA-BHMT1 axis alone or combination with folate may represent a potential and effective strategy for treating HHcy.

Increased de novo FFA synthesis and uptake play crucial roles in the pathogenesis of hepatic lipid deposition and NAFLD.^31^ Increased expressions of FASn and CD36, the key enzymes that control FFA synthesis and uptake, respectively, played crucial roles in the development and progression of NAFLD in humans and animals,^44,45^ and hepatic CD36 disruption improved HFD-induced steatosis.^46^ In the present study, we observed that hepatic RIMKLA overexpression significantly decreased the protein expression of FASn and CD36 in ND, HFD, and db/db mice livers. In contrast, RIMKLA deficiency increased their expressions. Furthermore, RIMKLA overexpression or siRNA inhibition of BHMT1 decreased or increased the promoter activities for FASn and CD36 genes, respectively. Hcy has been previously shown to activate AHR, inducing CD36 expression to increase hepatic lipid deposition.^12^ Moreover, Hcy also stimulated lipolysis in adipose tissues, resulting in ectopic lipid deposition.^6^ Recently, it had been reported that increased hepatic AP1 activity can cause inflammation to induce lipid deposition.^33,34^ However, the distinct mechanism(s) of AP1 activation and its induction of hepatic lipid deposition was poorly understood. Here we demonstrated that FASn and CD36 are the direct target genes of AP1, and Hcy activates it to induce their expressions, concurrently stimulating de novo FFA synthesis and uptake to promote hepatic lipid deposition and steatosis. Activation of AP1-FASn/CD36 axis by Hcy is an important mechanism of NAFLD. In the present study, we provided strong evidence that RIMKLA protected against Hcy-induced expressions of FASn and CD36. Hepatic RIMKLA-deficient mice exhibited more severe hepatic lipid deposition, elevated Hcy levels in liver and serum, and increased hepatic AP1 phosphorylation and FASn/CD36 expressions after Hcy feeding. These findings expanded the understanding on the regulation network of hepatic lipid synthesis and uptake. To inhibit AP1 by its specific antagonist such as SR11302 may be potential strategy for treating HHcy-associated NAFLD. Moreover, increased serum Hcy level also induces insulin resistance in white adipose to cause lipolysis, elevating serum FFA levels and enhancing hepatic FFA uptake. Accumulation of lipid in the liver then further inhibits RIMKLA-BHMT1 functional axis to exaggerate the dysregulation of lipid metabolism.

Regarding the roles of RIMKLA in hepatic glucose and lipid metabolism, several issues should be noted. Although RIMKLA regulates hepatic lipid metabolism by phosphorylating Thr45 of BHMT1, which is also likely regulated by other kinase(s) because RIMKLA deficiency only reduced but not completely abolished its phosphorylation in mouse livers. Because the current study was mainly focused on illuminating the mechanism of RIMKLA on repressing lipid synthesis and uptake, its mechanism on suppressing hepatic glucose production remained largely unrevealed. It may directly repress the expression of gluconeogenic/glycogenolytic genes, or affect their expression by regulating lipid metabolism, or via both pathways. Previous studies have shown that in hepatocytes, Akt is activated by its activating kinases (PI3K) in the cytoplasm. The phosphorylated Akt then translocates into the nucleus and phosphorylates FOXO1, leading to the nuclear exclusion of FOXO1 and inhibition of its transcriptional activity. This, in turn, negatively regulates the transcription of key genes involved in gluconeogenesis, such as phosphoenolpyruvate carboxykinase (PEPCK) and G6Pase.^47–49^ In other metabolic tissues, such as the pancreas, activation of the PI3K-Akt-FOXO1 pathway promotes glycogen synthesis and reduces gluconeogenesis, thereby alleviating the progression of type 2 diabetes.^50^ In adipocytes, the PI3K-Akt-FOXO1 signal is involved in the regulation of uncoupling protein 1 (UCP1) transcription, modulating brown adipose function and ameliorating obesity.^51^ In the present study, we observed that RIMKLA overexpression increased the protein expression and nuclear distribution of pAkt, and promoted nuclear exclusion of FOXO1, with decreased mRNA and protein levels of G6Pase in mouse livers and hepatocytes. This suggests that the inhibitory effect of RIMKLA on gluconeogenesis may be mediated through the activation of the Akt-FOXO1 pathway. However, further studies are needed to elucidate the specific mechanisms by which RIMKLA activates Akt.

In summary, RIMKLA is a new protein kinase that phosphorylates BHMT1 at Thr45 site to activate it. Under obese condition, hyperlipidemia and hyperinsulinemia repress RIMKLA to impair BHMT1 activity and elevate Hcy level, which activates AP1 to induce the transcriptions of FASn and CD36, stimulating de novo lipid synthesis and uptake. Impaired RIMKLA-BHMT1 axis promotes the vicious cycle among dysregulated lipid and Hcy metabolism to trigger the development and progression of NAFLD and diabetes. Activation of hepatic RIMKLA-BHMT1 functional complex represents a novel strategy for treating HHcy and metabolic disorders (Fig. 8m).

## Materials and methods

### Ethics approval statements

The physiological and biochemical characteristics of patients with NAFLD and individuals with non-NAFLD associated with HCC were detailed previously, with the ethics approval granted by Xijing Hospital, Fourth Military Medical University (Ethics Number: KY20172013-1).^52,53^ The serum samples from healthy subjects or patients with diabetes were provided by Beijing Luhe Hospital, Capital Medical University. The application for patient information was approved by the Research Ethics Committee Beijing Luhe Hospital, and informed consents were obtained from each participant (Ethics Number: 2016YFC0901200). All the animal experiments procedures conformed to the Animal Management Rules of the Ministry of Health of China, and the study protocols and use of animals were approved by Ethics Committee for Laboratory Animal Care and Use of Peking University Health Science Center (LA2019087).

### Materials

DL-homocysteine were purchased from Sigma-Aldrich (#H4628, Shanghai), and reagents for RT-PCR were purchased from Transgen Biotech (Beijing). RIMKLA antibodies (#PA5-101852) and monoclonal phosphor-Thr antibodies (#13-9200) from Invitrogen were used for western blotting at a dilution of 1:1000. Antibody against phosphor-Ser were purchased from Immunoway (USA). Mouse monoclonal BHMT1 antibody (#NBP2-75418) were from Novus Biologicals (USA). Antibody against phosphorylation of BHMT1 (Thr45) was generated by Biosynthesis Biotechnology Inc. (Beijing, China). Anti-pAkt (phosphorylated at Ser473 site) and anti-Akt antibodies, anti-pFOXO1 and anti-FOXO1 antibodies were from CST (#4060S, #9272S, #9461S, #2880S). G6Pase and PEPCK antibodies were from Santa Cruz Biotechnology (#sc-25840) and Bioworld (#BS6870). Rabbit polyclonal antibodies against CD36 molecule (#YT5585, Immunoway, USA) and fatty acid synthase (#ab22759, Abcam, UK) were used for western blotting. pAP1 (phosphorylated at Ser63 site of c-JUN subunit) antibody and AP1 antibody (anti-c-JUN) were from Abcam (#ab32385) and Abclonal (#A11378). β-actin antibodies (#TA-08, Origene, China) served as control.

### Real-time PCR assays

Total RNA of hepatocytes or livers were extracted under the manufacturer’s instructions (BioTeke Corporation Co., Ltd, China). The quality and concentration of RNA was assessed by Nanodrop One machine (Thermo, USA). cDNA synthesis was performed using TransScript Uni All-in One First-Strand Kit and followed by PCR amplification using SuperMix for qPCR provided by TransGen Biotechnology Inc. Housekeeping gene β-actin served as an endogenous control. Specific primers for were listed in Supplementary Table 4.

### Plasmid and siRNA transfection

Cells were placed into 6-well plates and replaced with fresh medium before transfection, then were transfected with 2 μg BHMT1 plasmid or its mutation BHMT1 Thr45 to Ala (T45A, His-tag), or AP1 plasmid for 24 h using transfection reagent (Quickshuttle, Biodragon Immunotechnologies Co., Ltd, China), the same concentration of pcDNA3.1 plasmid served as negative control. For BHMT1 knockdown, specific siRNAs targeting BHMT1 were synthesized by Guangzhou RiboBio Co., Ltd. The sequences were shown in the Supplementary Table 5. Cells were treated with 100 nM siBHMT1 or control sequence. Plasmids expressing homo RIMKLB were purchased from Youbio (G100212, pDONR223 vector, China). HepG2 cells were transfected with 2 μg RIMKLB plasmid for 24 h, and the same concentration of GFP plasmid (pGFP) served as negative control.

### Animals

C57BL/6 mice at the age of 7–8 weeks were fed on a ND or HFD containing 45% fat (Medicience, #MD17121, #MD12032), or HFD containing 60% fat (Research Diets #D12492) for the indicated times. db/m and db/db mice on the BKS background were used in this study. Mice were housed in standard animal laboratories with regular 12-h light-dark cycle and free access to water and food. 7–8 weeks male mice were fed with 1.8 g/L DL-Hcy (Sigma-Aldrich, #H4628, Shanghai) in drinking water for 8 weeks to induced HHcy mouse model. All the experiments procedures conformed to the Animal Management Rules of the Ministry of Health of China, and the study protocols and use of animals were approved by Ethics Committee for Laboratory Animal Care and Use of Peking University Health Science Center (LA2019087).

### Adenovirus- and adeno-associated virus-mediated overexpression of RIMKLA in mouse livers

C57BL/6 mice were injected with 1.0 × 10^9^ plaque forming unit (pfu) Ad-GFP or Ad-RIMKLA via tail veins. C57BL/6 mice fed on HFD containing 45% fat or ND for 3 months, or C57BL/6 mice fed on HFD containing 60% fat for 6 months (purchase from Gempharmatech Co., Ltd) were also injected with Ad-GFP or Ad-RIMKLA via tail veins, respectively. These mice were performed on different tests to monitor metabolic phenotypes and sacrificed at the 8–14th day post injection. The AAV8-GFP and AAV8-RIMKLA were constructed by WZ Biosciences Inc. 5 × 10^11^ virus particles (vg) AAV8-GFP or AAV8-RIMKLA were injected into the tail veins of db/db or db/m mice. After a series of tests were successively conducted at the indicated times, the serum and tissues were collected for biochemical analyses.

### Generation of hepatocyte-specific RIMKLA knockout mice

Two loxp sites were inserted into both ends of mouse RIMKLA exon1 using Cas9/gRNA method (Beijing Viewsolid Biotechnology Co., Ltd, China). Conditional hepatic RIMKLA deletion happened when RIMKLA-flox mice were crossed with mice specifically expressing Cre gene promoted by Albumin (Alb-Cre). 8–10-week-old male mice were used for further phenotype analysis.

### Human liver and serum samples

The biochemical characteristics and ethical issue of patients with NAFLD and individuals with non-NAFLD associated with HCC were provided in Supplementary Table 6. The embedding liver slices were used to detect the expressions of RIMKLA and BHMT1. The physiological and biochemical characteristics of patients with NAFLD and non-NAFLD associated with HCC were detailed previously, with the ethics approval granted by Xijing Hospital, Fourth Military Medical University (Ethics Number: KY20172013-1).^52,53^ The serum samples from healthy subjects or patients with diabetes were provided by Beijing Luhe Hospital, Capital Medical University. The demographic characteristics were shown in Supplementary Table 7. The application for patient information was approved by the Research Ethics Committee Beijing Luhe Hospital, and informed consents were obtained from each participant (Ethics Number: 2016YFC0901200).

### OGTT, PTT, ITT, hyperinsulinemic-euglycemic clamp assays

OGTT, PTT, and ITT were performed at indicated times in different animal models. The detailed protocols were introduced previously.^54^ The hyperinsulinemic-euglycemic clamp assays were also performed followed our published method.^54^ Simply, HFD mice injected with Ad-GFP or RIMKLA were infused with insulin through the external jugular vein at a rate of 4 mU/kg/min after 12-h fasting. The glucose infusion pump started to work until the blood glucose was stabilized around 100–150 mg/dL for half an hour. The blood glucose was tested every 10-min intervals, lasting for 2 h. The glucose infusion rate (GIR) was recorded and averaged to compare the differences of indicated groups.

### In vivo fatty acid uptake test

Fatty acid uptake was performed based on previous research^46,55^ BODIPY^TM^FL C_16_ was purchased from Invitrogen (#D3821) and animals were injected by intraperitoneal injection at the dose of 0.5 μg/g body weight. Serum samples at 0, 1, 3 and 5 h post injection were collected via the tail vein. At the time point of 5 h, the livers were harvested and homogenized in RIPA lysis buffer. Finally, serum (20 µL) and hepatic (100 µL liver lysates) RFU levels (relative fluorescence unit) were detected using a fluorescent plate reader (EX 485 nm, EM 515 nm). Fluorescence data were analyzed by subtracting the fluorescence signal from saline-injected animals. Hepatic RFU levels were normalized to the weight of extracted tissue.

### Magnetic resonance imaging

Mice were anesthetized, and then placed in a sealed compartment with adequate gas supply and positioned horizontally. All lipid imaging data including liver lipid and body fat distribution were collected and quantified using a Siemens Trio-Tim scanner, Siemens Syngo software version MR B17 with reference to previous publications.^56,57^

### Prediction of kinase domain and protein-docking prediction using ZDOCK

Domain analysis and model building - The InterPro database (https://www.ebi.ac.uk/interpro/) and Uniprot database (https://www.uniprot.org) were used to predict the presence of kinase domains and binding sites. The PDB formats of the RIMKLA (AF_AFQ6PFX8F1) and BHMT1 (AF_AFO35490F1) structural domains downloaded from the Protein Data Bank PDB database (http://www.rcsb.org/) were used as initial models for protein docking.^58^ Structural figures were prepared using PyMOL (https://pymol.org/2/).^59^ To predict the interaction affinities between RIMKLA and BHMT1, a protein docking program named ZDOCK (https://zdock.wenglab.org/) was used to identify the docking sites and calculate the ZDOCK scores, as detailed in a previous study.^60^

### Isolation of primary hepatocytes

As previously described,^61^ mouse primary hepatocytes were isolated using collagenase I and purified by centrifugation. The fresh hepatocytes were resuspended and seeded in 6-well attachment plates and the medium were replaced with fresh RPMI 1640 containing 10% FBS after 6 h incubation and adherence. Then the cells were administered with different treatments.

### Western blotting

Cells or tissues were lysed in Roth lysis buffer (50 mM HEPES, 150 mM NaCl, 1% of Triton X-100, 5 mM EDTA, 5 mM EGTA, 20 mM NaF, pH 7.4) containing inhibitors of proteinase and protein phosphatase. After protein extraction by centrifugation, 20–40 µg proteins were separated by SDS-PAGE. The nitrocellulose membranes were incubated with primary antibodies overnight at 4 °C, followed by washing with 1×TBST for three times and incubated with horseradish peroxidase-conjugated secondary antibody for 2 h. Proteins bands were visualized with enhanced ECL Chemiluminescence Kit (Biodragon Immunotechnologies Co., Ltd, China).

### Immunofluorescent staining

Cells seeded on coverslips were washed with 1×PBS for 3 times and then fixed in 4% paraformaldehyde for 20 min. After permeabilized with 0.05%Triton X-100 for 10 min and blocked in 1% BSA for 10 min at room temperature, the primary antibody was incubated at a 1:50 dilution overnight (4 °C). On the second day, the second fluorescent antibodies (1:100) were incubated for 1 h followed by DAPI incubation (1:5000) for 10 min. While Neutral Lipids were stained using LipidTOX^TM^ (H34476, Invitrogen, USA) at a dilution of 1:200 for 20 min. The images were scanned using a confocal microscopy (Leica).

### Co-Immunoprecipitation

Co-Immunoprecipitation was performed based on the protocol of Pierce Crosslink Immunoprecipitation Kit (#26147, Thermo, USA) and our previous reports.^62^ In brief, 50 μL protein A/G Sepharose beads were loaded and crosslinked with 10 µg RIMKLA or BHMT1 antibodies (rabbit or mouse IgG used as negative control) for 1 h. 1 mg total proteins extracted from mouse primary hepatocytes were precleaned with control agarose resin for another 1 h. Then the total proteins were incubated with the protein A/G Sepharose beads containing primary antibodies overnight at 4 °C. The purified proteins were eluted using elution buffer and subjected to immunoblotting assays.

### Biochemical measurements

Biochemical measurements were performed to quantify the levels of hepatic and serum TG, CHO, Hcy, and Met. Hepatic TG and CHO contents were examined using kits (Applygen Technologies, Inc, China). Serum TG and CHO levels were measured using GPO-PAP or CHOD-PAP methods according to the instructions (Biosino Biotechnology and Science, Inc). Mouse/human Hcy ELISA kits were from Dogesce (Beijing, China), and mouse Met ELISA kit was from Ruixin Biotechnology (Quanzhou, China).

### Extraction of cytosol and nuclear fractions

Nuclear and cytoplasmic fractions were extracted from HepG2 cells or primary mouse hepatocytes using an extraction kit according to the manufacturer’s instructions (#P1200, Applygen Technologies Inc, China). Quantification of nuclear or cytoplasmic protein concentrations were performed using Pierce BCA kit (ThermoFisher Scientific, USA). The extraction fractions were analyzed by immunoblotting. β-actin and LaminB1 served as the loading control of cytosolic or nuclear proteins, respectively.

### Glucose production assay

Cells were cultured in 10% FBS RPMI 1640 (mouse hepatocytes, L02 cells) or DMEM (HepG2 cells). After infected with Ad-GFP or Ad-RIMKLA for 24 h, cells were washed three times using sterile PBS buffer and the cultured medium was exchanged with glucose- and phenol red-free RPMI 1640 or DMEM, with a supplementation of 20 mM sodium lactate and 2 mM sodium pyruvate for 13 h, followed by the addition of insulin (10 nM) for 3 h. Glucose content in the medium was analyzed using Glucose Assay Kit (Sigma-Aldrich, GAGO20, USA) and normalized by cellular protein content.

### Measurement of BHMT1 enzyme activity

The protocol was performed detailedly in previous research.^63^ Briefly, hepatocytes were suspended in the extraction buffer and subjected to three freezing-thaw cycles. Total proteins were extracted by centrifugation at 12000 rpm for 10 min. Then DL-Hcy (100 mM stock, 1 μL/sample), [Me-^3^H] betaine (0.05 μCi/sample), KH_2_PO_4_ (100 mM stock, 48 μL/sample), 2-ME (5 mM stock, 1 μL/sample) were prepared in reacting mix. Each assay consisted of 50 μL protein extracts and 50 μL reacting mix. After incubation at 37 °C for 3 h, the sample tubes were transferred onto ice, followed with the addition of 500 μL cold water and 500 μL 50% Dowex slurry to bind ^3^H-Met. After washed for 4 times using ice cold water, the products were eluted by 1.5 M HCl and were counted in the scintillation fluid. The BHMT1 enzyme activity was presented as CPM (cycles per minute, radioactive unit of the liquid scintillation counter) of product (Met) per hour per mg of input protein after subtraction the negative control CPM. All samples were assayed in triplicate.

### Purification of RIMKLA and pull-down assays

6×His fusion proteins (RIMKLA-His) were expressed in *E. coli* BL21 (WZ Biosciences Inc, China), induced with 0.2 mM isopropyl-1-thio-β-D-galactopyranoside (IPTG) for 4 h at 37 °C and purified according to the protocols of Beyotime Biotechnology (#P2226, China). Equal amounts of bacteria lysates expressed empty vector (PET28a) or RIMKLA-His fusion proteins were incubated with BeyoGold™ His-tag Purification Resin for 1 h at 4 °C before washed with lysis buffer for 3 times. Then, total cell extracts were added and incubated with the resins overnight. After centrifugation and elution, the resin-bound proteins were eluted and dissolved into 5×loading buffer, followed with the detection by western blotting using antibodies against RIMKLA or BHMT1.

### Luciferase reporter assay

The promoter regions of mouse FASn gene (−2000 bp to +250 bp) and CD36 gene (−2000 bp to +250 bp) were separately cloned into the pGL3-Basic vector containing firefly luciferase gene. FASn or CD36 promoters were cotransfected with AP1 expression plasmid or pcDNA3.1 control plasmid using QuickShuttle transfection reagent (Beijing Biodragon Immunotechnologies Co., Ltd, China), together with renilla luciferase overexpression in HepG2 cells, which was used as a positive transfection control. After overexpression of RIMKLA by adenovirus or transfection of siBHMT, HepG2 cells were transfected with FASn or CD36 promoters, together with renilla luciferase overexpression, which was used as a positive transfection control. After 24 h treatment, the luciferase activities of firefly and renilla were determined using Dual-Luciferase Reporter Assay (Promega, USA).

### RIMKLA directly induces phosphorylation of BHMT1

For recombinant protein RIMKLA purification, mouse *Rimkla* (NM_177572.4) gene was cloned to pET-28a vector for constructing pET-28a-Rimkla-His recombinant plasmid, which was then expressed in *E. coli* BL21 (DE3). The protein extract supernatant containing RIMKLA-His protein was purified using Ni-NTA agarose resin, and eluted into desalting buffer (20 mM Tris, 100 mM KCl, 1 mM DTT, 10 mM MgCl_2_, 1% Tween 20, 5% GLY, pH=8.0) by desalting column. The in vitro phosphorylation assay was detailed previously.^64^ Briefly, the BHMT1 protein from mouse hepatocytes was immunoprecipitated using protein A/G Sepharose beads crosslinked with BHMT1 antibody, the immunoprecipitated BHMT1 protein (20 µg) was incubated with or without recombinant RIMKLA-His (50 ng/mL) in kinase buffer (25 mM HEPES, 1 mM DTT, 50 mM NaCl, 2 mM EGTA, 5 mM MgSO_4_) with 50 µM ATP at 37 °C for 2 h, and then subjected to immunoblotting assays. Antibodies against phosphor-BHMT1^T45^ were purified from serum of New Zealand Rabbit guests, which were immunized for several times with immunogen (T45 modified peptides) (#bs-39011R, Biosynthesis Biotechnology Inc).

### Phosphokinase kinetics assay

Recombinant BHMT1 protein with different concentrations (0–20 μM) was added as a substrate to react with 50 ng/mL recombinant RIMKLA protein at room temperature for 15 min to 1 h. The amount of ADP produced by the RIMKLA-BHMT1 kinase reaction was detected using the AmpliteTM Universal Fluorescent Kinase Assay Kit (#31001, AAT Bioquest, USA). The fluorescence intensity was monitored by a Varioskan multimode reader (Thermo Scientific, USA) with Ex/Em = 540/590 nm (cutoff = 570 nm). The phosphokinase kinetic parameters of RIMKLA on BHMT1 were calculated based on the reaction time, the concentration of substrate BHMT1, and the produced ADP. The obtained data were fitted to the Michaelis-Menten equation using GraphPad Prism software to derive the apparent V_max_ and K_m_, according to the previous studies.^65,66^

### Enzyme kinetics assay of phosphorylated and dephosphorylated BHMT1

Mouse hepatocytes were infected with wild-type or mutant AAV8-BHMT1 for 48 h, and then infected with either Ad-GFP or Ad-RIMKLA for additional 24 h, ensuring similar cell numbers in each group. DL-Hcy (500 μM) was added to each group at 2 h, 1 h, 30 min, or 0 min before cell collection. After treatments, hepatocytes were harvested simultaneously, and intracellular Hcy level was detected using a mouse ELISA kit (#DG30759M, Dogesce, Beijing, China), normalized by protein content. The kinetics of Hcy metabolism by phosphorylated and dephosphorylated BHMT1 were calculated, according to the previous studies.^65,67^ Curve fitting was performed using GraphPad Prism software, and the average reaction velocity was compared among different groups.

### Statistical analysis

All data were analyzed using GraphPad Prism 8.0 (GraphPad Prism Software, Inc, San Diego, CA) and presented as mean ± SEM. The normal distributed data were determined by Shapiro–Wilk test. Student’s t-test or Mann–Whitney U test was used to compare two groups, one-way/two-way ANOVA followed by Bonferroni post hoc analysis/Tukey’s tests were used to compare normal distributed data for multiple groups. Non-normal distributed dada among multiple groups were analyzed by Kruskal–Wallis test followed by Dunn’s post hoc analysis. *P* values were two-tailed, and values <0.05 were considered as statistically significance.

## Supplementary information

Supplementary Figures and Tables

Original WB images

## Data Availability

All the datasets presented in the paper are available from the corresponding author upon reasonable request.
