# Correction: Ribosomal modification protein rimK-like family member A activates betaine-homocysteine S-methyltransferase 1 to ameliorate hepatic steatosis

**DOI:** 10.1038/s41392-024-02054-1

**Published:** 2024-12-11

**Authors:** Han Yan, Wenjun Liu, Rui Xiang, Xin Li, Song Hou, Luzheng Xu, Lin Wang, Dong Zhao, Xingkai Liu, Guoqing Wang, Yujing Chi, Jichun Yang

**Affiliations:** 1 Department of Physiology and Pathophysiology, School of Basic Medical Sciences, State Key Laboratory of Vascular Homeostasis and Remodeling, Center for Non-coding RNA Medicine, Peking University Health Science Center, Beijing, China; 2 Department of Endocrinology, The Second Affiliated Hospital, School of Medicine, Zhejiang University, Hangzhou, China; 3 Medical and Health Analysis Center, Peking University, Beijing, China; 4 Department of Hepatobiliary Surgery, Xi-Jing Hospital, Fourth Military Medical University, Xi’an, China; 5 Department of Endocrinology, Beijing Luhe Hospital, Capital Medical University, Beijing, China; 6 Department of Hepatobiliary and Pancreatic Surgery, General Surgery Centre, First Hospital of Jilin University, Changchun, China; 7 Key Laboratory of Pathobiology Ministry of Education, College of Basic Medical Sciences, Jilin University, Changchun, China; 8 Department of Central Laboratory and Institute of Clinical Molecular Biology, Department of Gastroenterology, Peking University People’s Hospital, Beijing, China; 9 Department of Cardiology, Peking University Third Hospital, Beijing, China

**Keywords:** Endocrine system and metabolic diseases, Pathogenesis

Correction to: *Signal Transduction and Targeted Therapy* 10.1038/s41392-024-01914-0, published online 08 August 2024

In the process of collating the raw data, the authors noticed one inadvertent mistake in supplementary figure 9f after online publication of the article^1^. The β-actin in supplementary figure 9d was repeatedly inserted as β-actin (upper) in supplementary figure 9f. The correct data and corresponding original image are provided as follows. This correction does not affect the key findings (the existing published results or the discussion). The original article has been corrected.
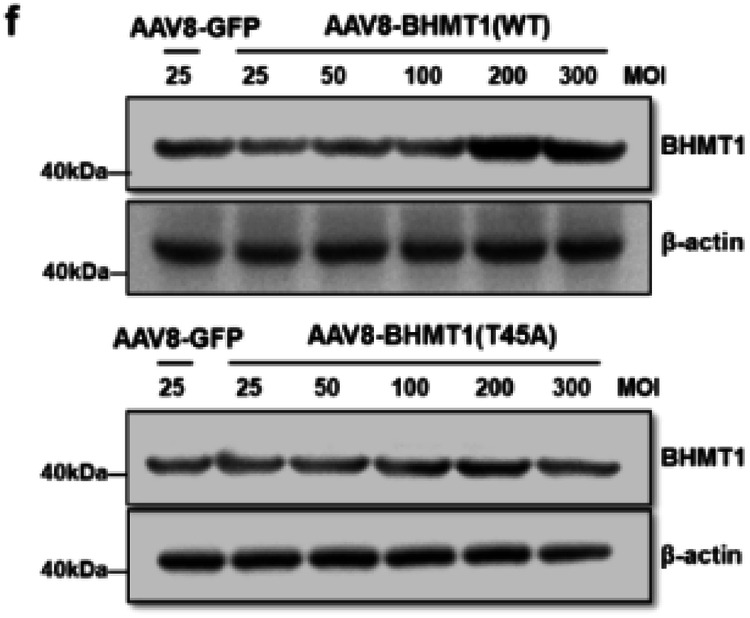

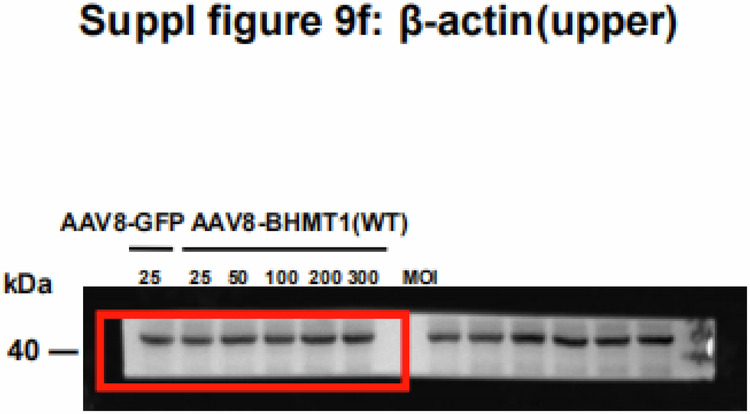


Supplementary Fig. 9f. BHMT1 protein levels in mouse hepatocytes administrated by different doses of AAV-BHMT1(WT) or AAV-BHMT1(T45A) for 48 h.

In the self-check after publication, several mismatches between the blots presented in the figures and the original uncropped blots were also identified.
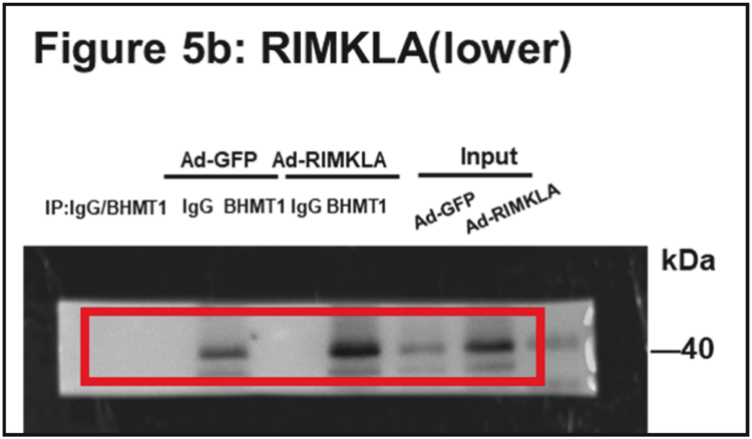


In Fig. 5b, the original WB image was chosen from another experiment performed in the same condition, causing mismatch. The above is the matched original WB image. The previously mismatched and newly matched images represented the different experiments performed in the same condition.
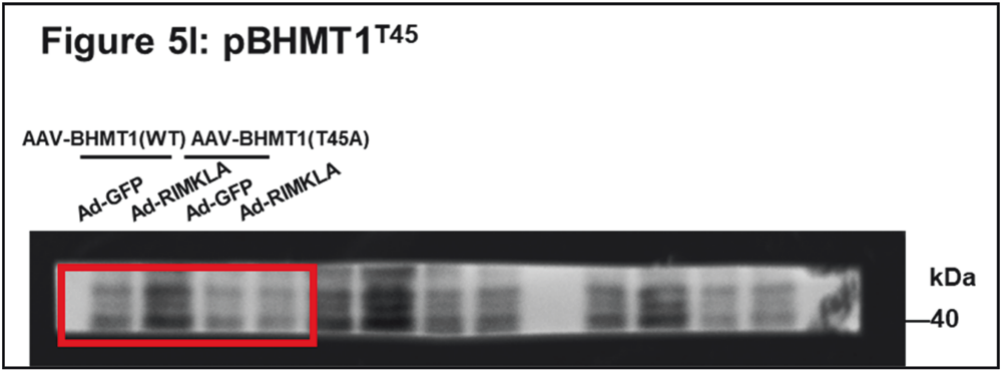


In Fig. 5l, the original WB image was chosen as another image obtained from the same membrane but with different exposure, causing mismatch. The above is the matched original WB image. The previously mismatched and newly matched images represented the same experiment.
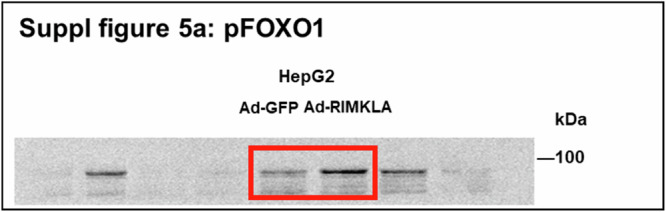


In Supplementary Fig. 5a, the previous original WB image was chosen from another experiment performed in the same condition, causing mismatch. The previously mismatched and newly matched images represented the different experiments performed in the same condition.
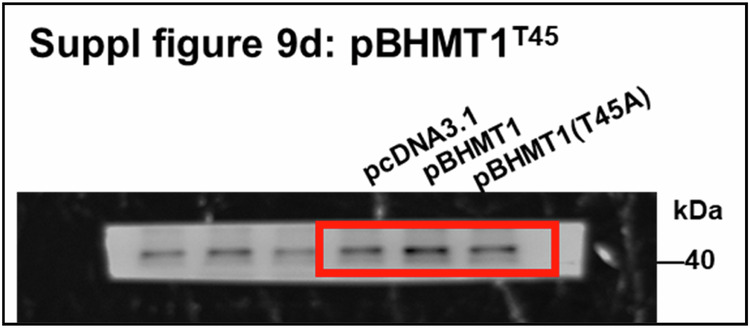


In Supplementary Fig. 9d, the original WB image was chosen from another experiment performed in the same condition, causing mismatch. The above is the matched original WB image. The previously mismatched and newly matched images represented the different experiments performed in the same condition.
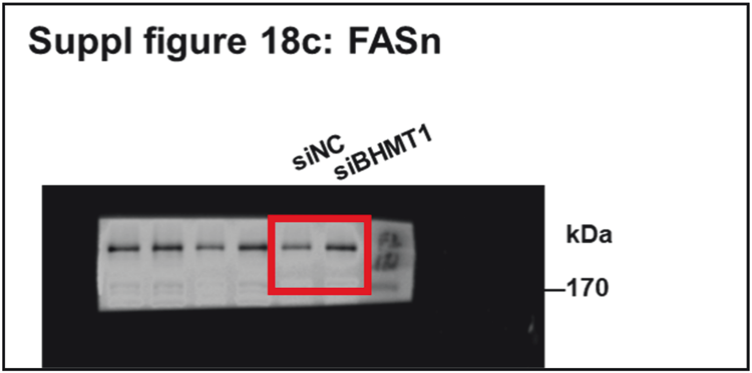


In Supplementary Fig. 18c, the original WB image was chosen from another experiment performed in the same condition, causing mismatch. The above is the matched original WB image. The previously mismatched and newly matched images represented the different experiments performed in the same condition.

These corrections have no effect on the description and meaning of each figure and overall conclusion.

The original article has been corrected.

## Supplementary information

Supplementary Materials
