# Supplementary material for: Ribosomal modification protein rimK-like family member A activates betaine-homocysteine S-methyltransferase 1 to ameliorate hepatic steatosis
Source: Signal Transduct Target Ther. 2024 Aug 8;9:214. doi: 10.1038/s41392-024-01914-0 (PMC11310345; doi:10.1038/s41392-024-01914-0)
Supplement: Supplementary file 1 — Supplementary Figures and Tables [file 41392_2024_1914_MOESM1_ESM.docx]

Supplementary Materials for

Ribosomal modification protein rimK-like family member A activates betaine-homocysteine S-methyltransferase 1 to ameliorate hepatic steatosis

Han Yan, Wenjun Liu, Rui Xiang, Xin Li, Song Hou, Luzheng Xu, Lin Wang, Dong Zhao, Xingkai Liu, Guoqing Wang, Yujing Chi, Jichun Yang

Correspondence to: yangj@bjmu.edu.cn; chiyujing@bjmu.edu.cn; qing@jlu.edu.cn; xingkailiu@foxmail.com

**This PDF file includes:**

Figures. S1 to S22

Tables S1 to S7


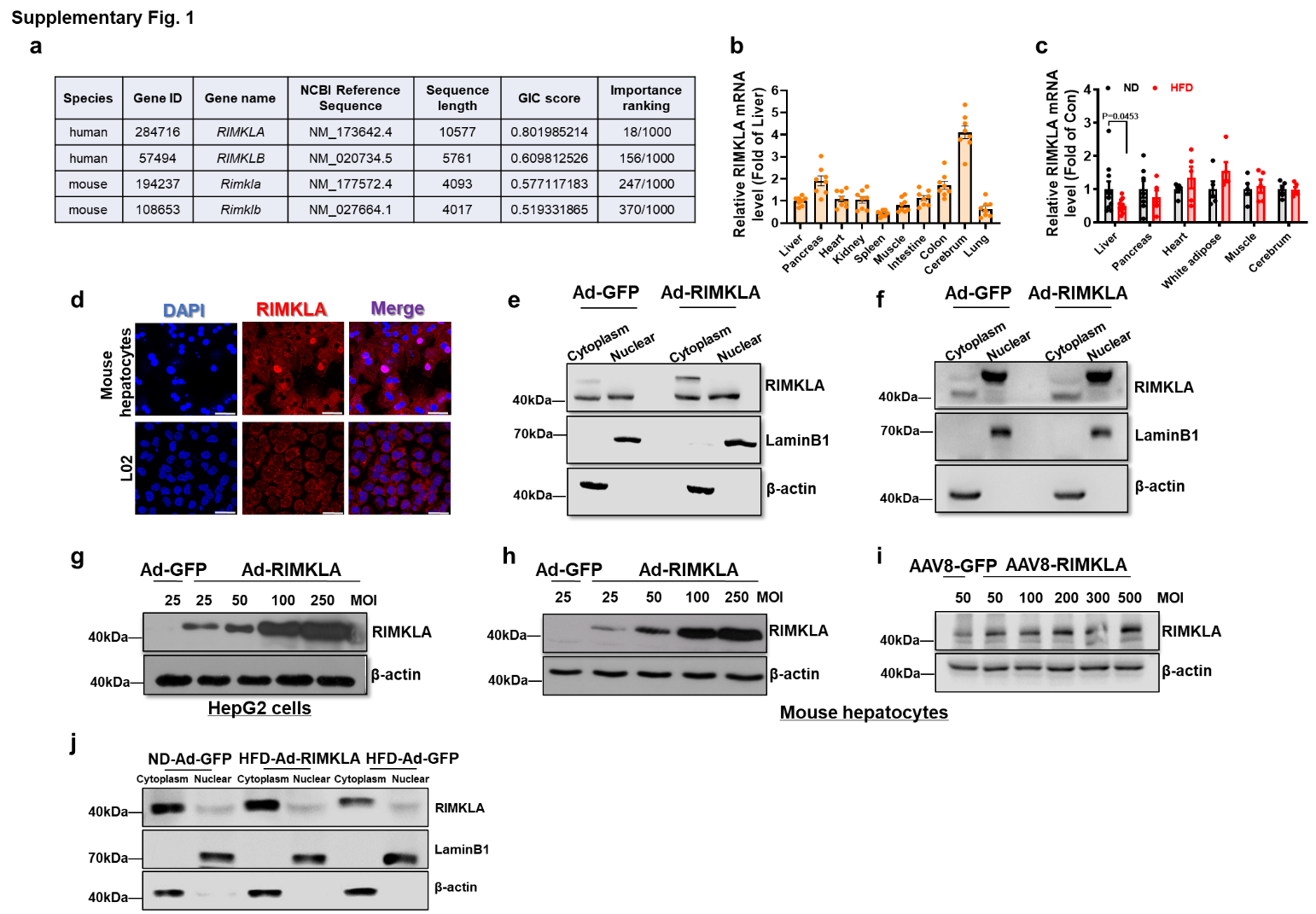


Figure. S1.

**Gene essentiality prediction of RIMKLA and RIMKLB.**

**a** Predicted essential score of human and mouse *RIMKLA/Rimkla* genes as well as *RIMKLB*/*Rimklb*, which were predicted by GIC method (http://www.cuilab.cn/gic). **b** RIMKLA mRNA expression profile among mouse tissues. *n*=8. **c** The RIMKLA mRNA expression in main metabolic tissues of high fat diet (HFD) mice in comparison with those of normal diet (ND) mice. *n=*5-11. **d** Determination of RIMKLA protein distribution by immunofluorescent staining in mouse hepatocytes and L02 cells. **e-f** Determination of RIMKLA protein distribution in cytoplasm and nuclear of HepG2 cells (**e**) and mouse primary hepatocytes (**f**) treated with Ad-GFP or Ad-RIMKLA for 24 hours. *n*=3. **g-h** Verification of Ad-RIMKLA in HepG2 cells (**g**) and mouse hepatocytes (**h**). Cells were infected by different doses of adenovirus for 24 hours before assays. **i** Verification of AAV8-RIMKLA in mouse hepatocytes. **j** Determination of RIMKLA protein distribution in cytoplasm and nuclear of livers in Ad-GFP- or Ad-RIMKLA-injected mice fed on HFD for 3 months, as well as in Ad-GFP-injected ND mice. *n*=3. Cells were infected with different doses of AAV8-RIMKLA (50, 100, 200, 300, 500 MOI) for 48 hours. For (**d**), Blue, DAPI; Red, RIMKLA; Purple, Merge. Scale bar: 50 $\mu m.$


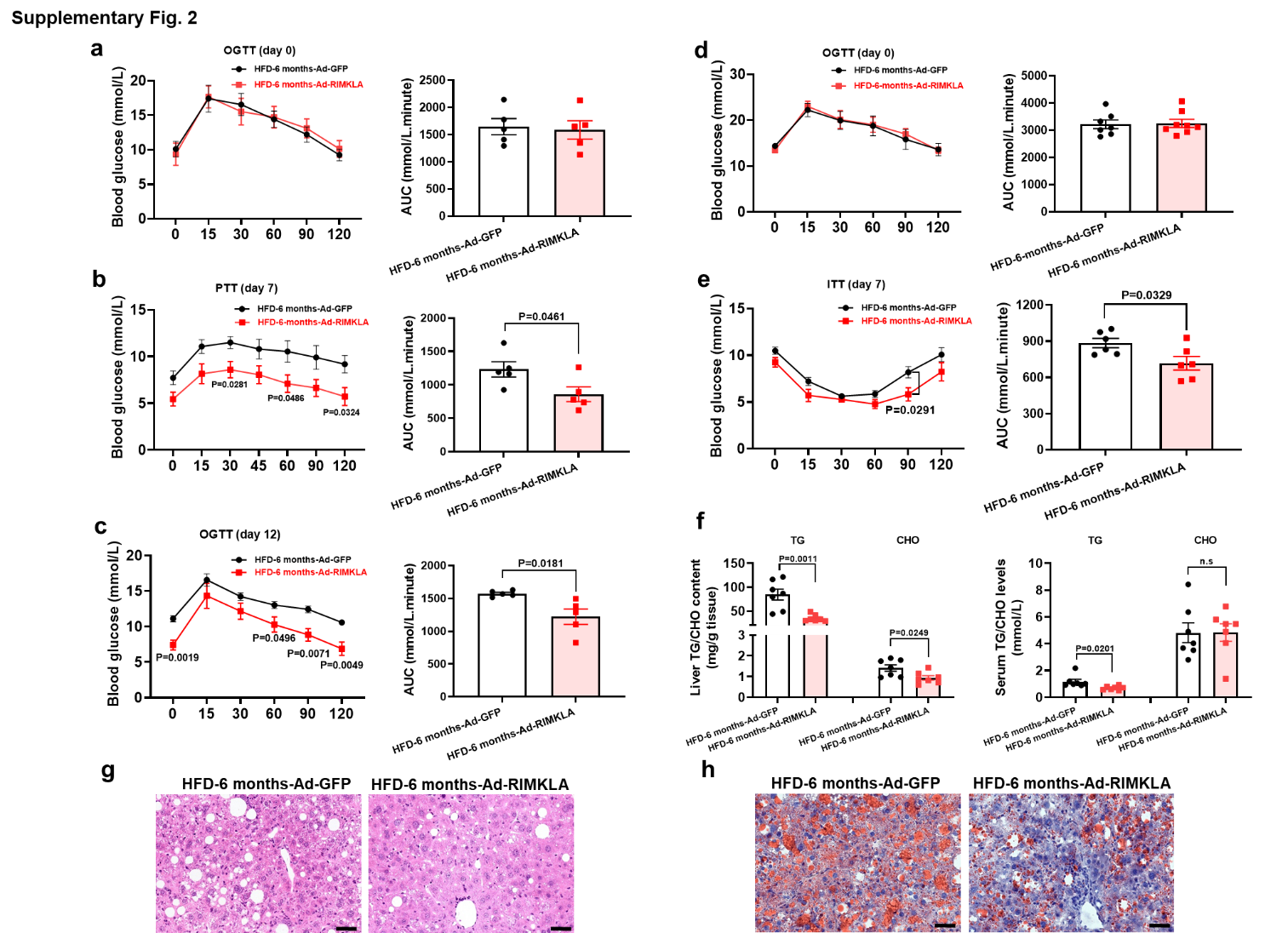


Figure. S2.

**Hepatic RIMKLA overexpression improves dysregulated glucose and lipid metabolism in mice fed on HFD for 6 months.**

**a** Mice fed on HFD for 6 months (purchase from Gempharmatech Co., Ltd) were classified into two groups using OGTT before adenoviral injection. *n*=5. **b-c** OGTT and PTT were performed at day 7 and 12, respectively post virus injection. *n*=5. **d** Another set of mice fed on HFD for 6 months were classified into two groups using OGTT before adenoviral injection. *n*=7-8. **e** ITT was performed at day 7 post virus injection. *n*=6. **f** Hepatic RIMKLA overexpression on hepatic and serum TG (triglyceride) and CHO (cholesterol) levels in fed on HFD for 6 months. *n*=7. **g** Representative liver histology images of two groups of mice by Hematoxylin-Eosin staining. Scale bar: 50 µm. **h** Representative Oil Red O staining images of livers in two groups of mice. Scale bar: 50 µm. Statistical P values were marked in each panel. The data and AUC were calculated using student’s t-test.


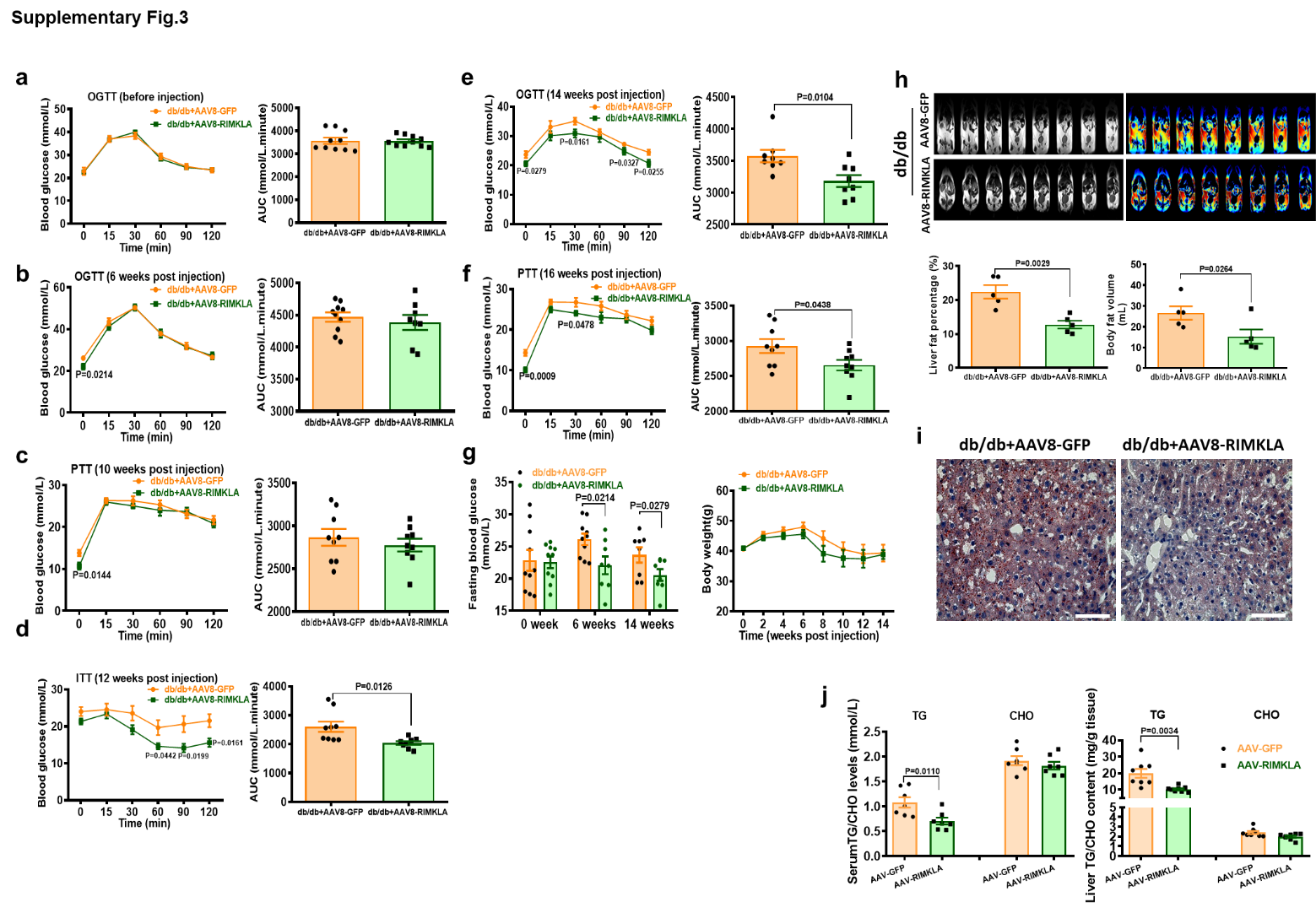


Figure. S3.

**Hepatic RIMKLA overexpression using AAV8 infection on glucose and lipid metabolism in db/db mice.**

**a** 8-week-old db/db mice were classified into two groups using OGTT before AAV8 injection. *n*=10 for each group. **b-c** OGTT and PTT were performed at 6 and 10 weeks, respectively post virus injection (5 × 10^11^ vg per animal). *n*=8-10. **d-f** ITT, OGTT and PTT were performed at 12, 14, 16 weeks post virus injection, respectively. *n*=8-9. **g** Fasting blood glucose levels (left panel) at 0, 6, 14 weeks post virus injection and body weight (right panel) monitoring every two weeks. *n*=8-10. **h** MRI analysis was performed at 10 weeks post virus injection. Representative scanning images were shown on the upper panel, and quantitative data of liver fat and body fat were shown on the lower panel. *n*=5. **i** Representative Oil Red O staining images for the livers of db/db mice injected with AAV8-GFP or AAV8-RIMKLA. Scale bar: 20 µm. **j** Serum and hepatic TG/CHO levels in the indicated groups. *n*=7-8. P values for left panels of (**a-f**) and right panels of (**g**) were analyzed by two-way ANOVA with Tukey’s tests, for AUC data of (**a-f**), left panels of (**g**), and (**f, j**) were analyzed by student’s t-tests.


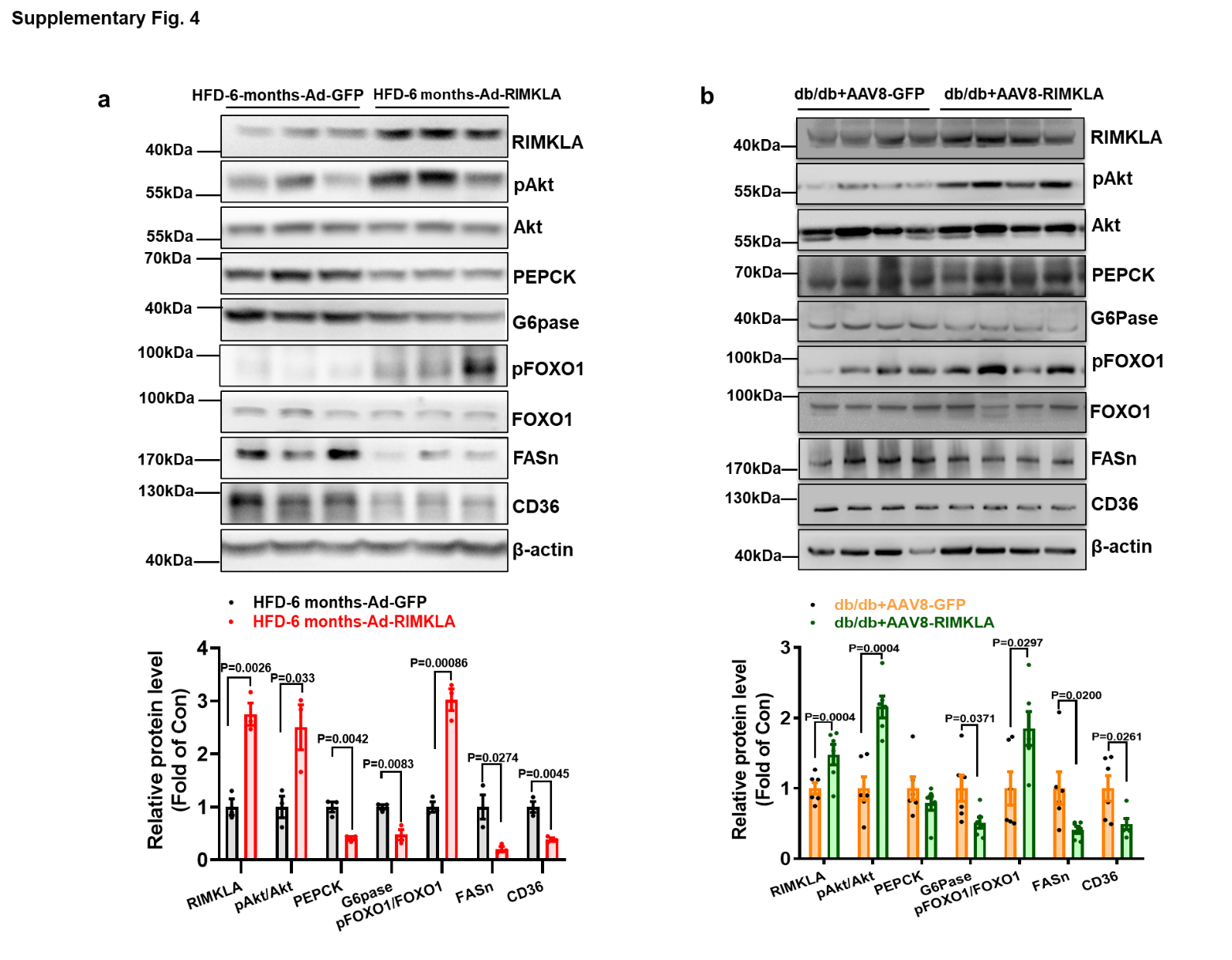


Figure. S4.

**RIMKLA injection inhibites the lipogenic genes protein expression in obese mice.**

**a** Expressions of related metabolic genes (RIMKLA, pAkt, Akt, PEPCK, G6Pase, pFOXO1, FOXO1, FASn, CD36) in Ad-GFP- and Ad-RIMKLA-injected mouse livers fed on HFD for 6 months. *n*=3*.* **b** Expressions of related metabolic genes (RIMKLA, pAkt, Akt, PEPCK, G6Pase, pFOXO1, FOXO1, FASn, CD36) in AAV8-GFP- and AAV8-RIMKLA-injected db/db mouse livers. *n*=6. Statistical P values were marked in each panel. The data were calculated using student’s t-test.


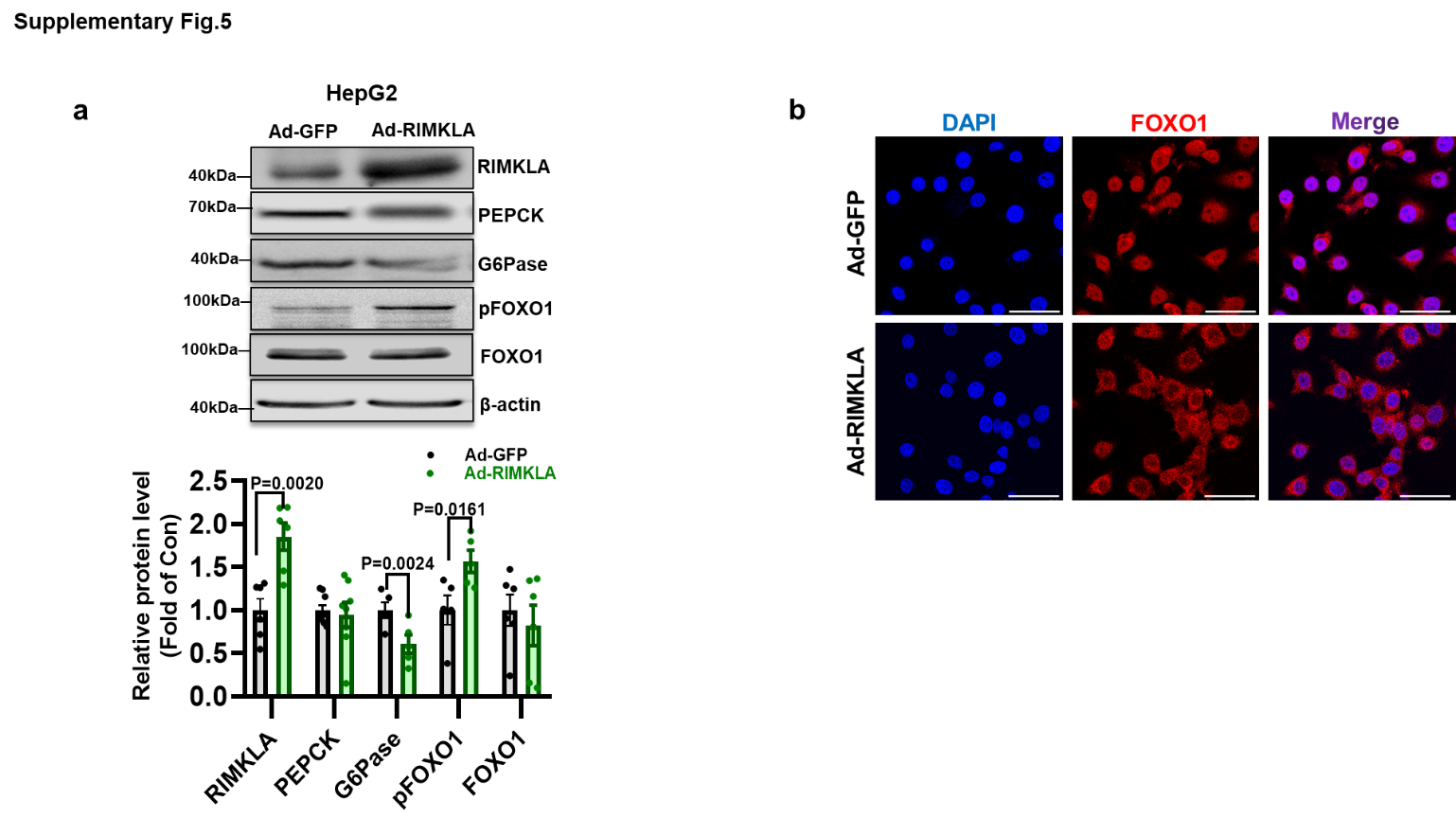


Figure. S5.

**Gluconeogenic gene expressions are inhibited by RIMKLA overexpression in HepG2 cells. a** Expressions of gluconeogenic genes and Akt phosphorylation after RIMKLA overexpression in HepG2 cells. Cells were infected with Ad-GFP/Ad-RIMKLA for 24 hours before assays. *n*=5-9. **b** Immunofluorescent image of FOXO1 in HepG2 cells after 24-hour Ad-GFP/Ad-RIMKLA treatment. Blue, DAPI; Red, FOXO1; Purple, Merge. Scale bar, 50 µm. Statistical P values were marked in each panel. P values for (**a**) between two groups were calculated using student’s t-test.


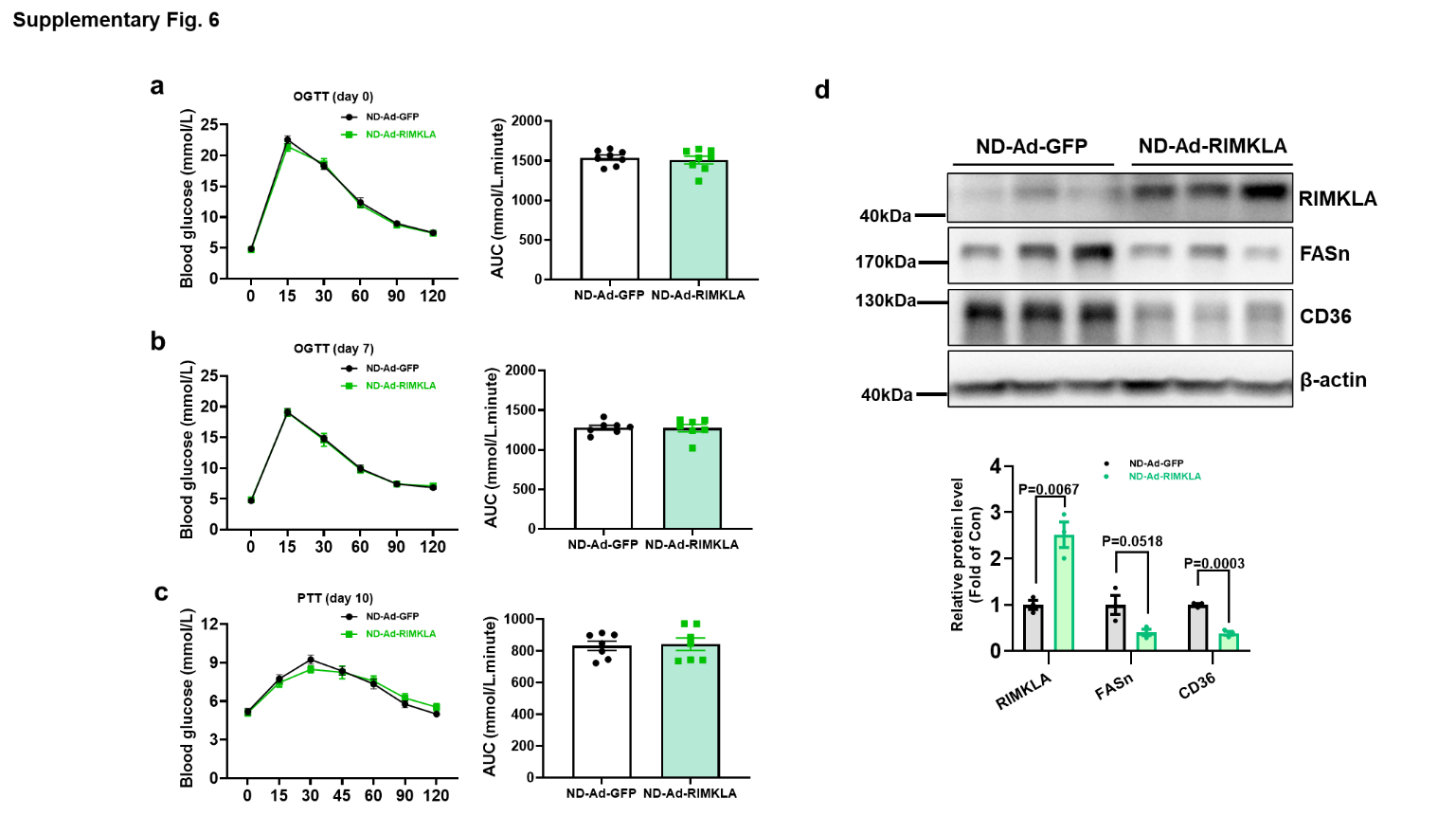


Figure. S6.

**Hepatic RIMKLA overexpression inhibits lipogenic gene expressions in mice fed on ND.**

**a** ND Mice were classified into two groups using OGTT before adenoviral injection. *n*=8. **b-c** OGTT and PTT were performed at day 7 and 10, respectively post virus injection. *n*=7. **d** Hepatic RIMKLA overexpression inhibited lipogenic genes expression in mice fed on ND. *n*=3. Statistical P values were marked in each panel. The data were calculated using student’s t-test.


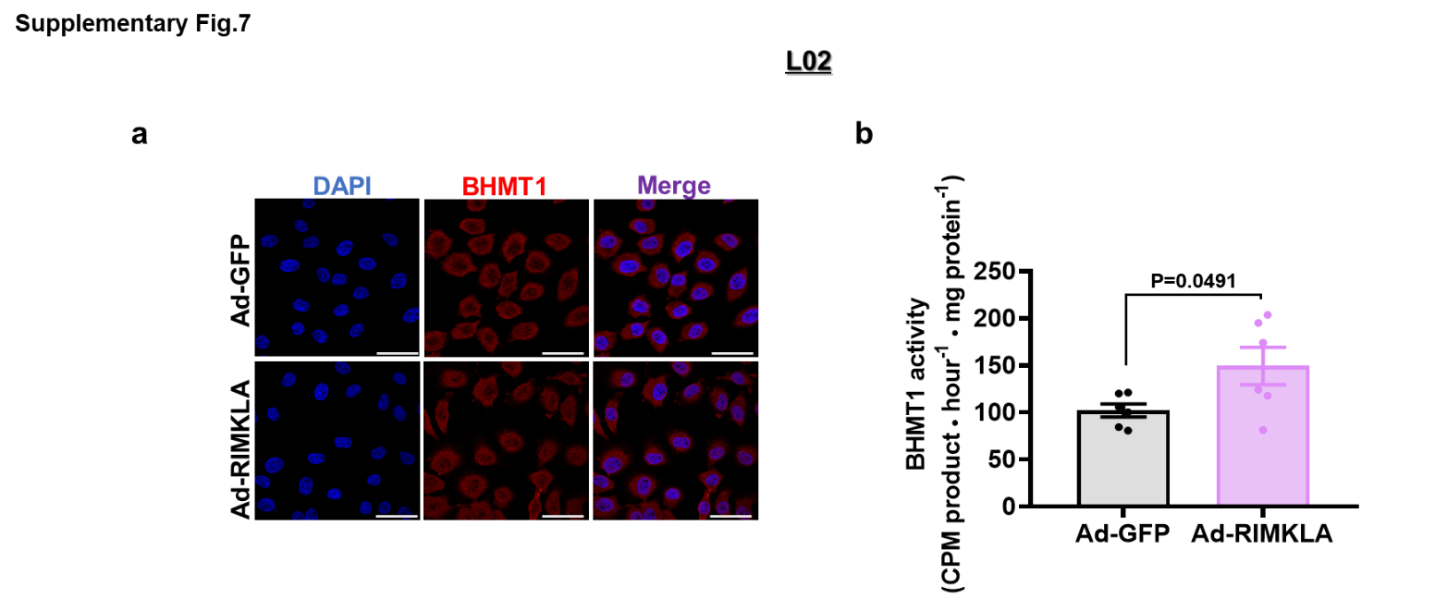


Figure. S7.

**RIMKLA overexpression increases BHMT1 activity in human hepatocytes.**

**a** Confocal image of BHMT1 protein in L02 cells after treatment with Ad-GFP/Ad-RIMKLA for 24 hours. The images were the representatives of three independent tests. Blue, DAPI; Red, BHMT1; Purple, Merge. Scale bar, 50 µm. **b** Measurement of cellular BHMT1 activity in L02 cells after infection with Ad-GFP/Ad-RIMKLA for 24 hours. *n*=6. Statistical P values were marked in each panel. P value was calculated using student’s t-test.


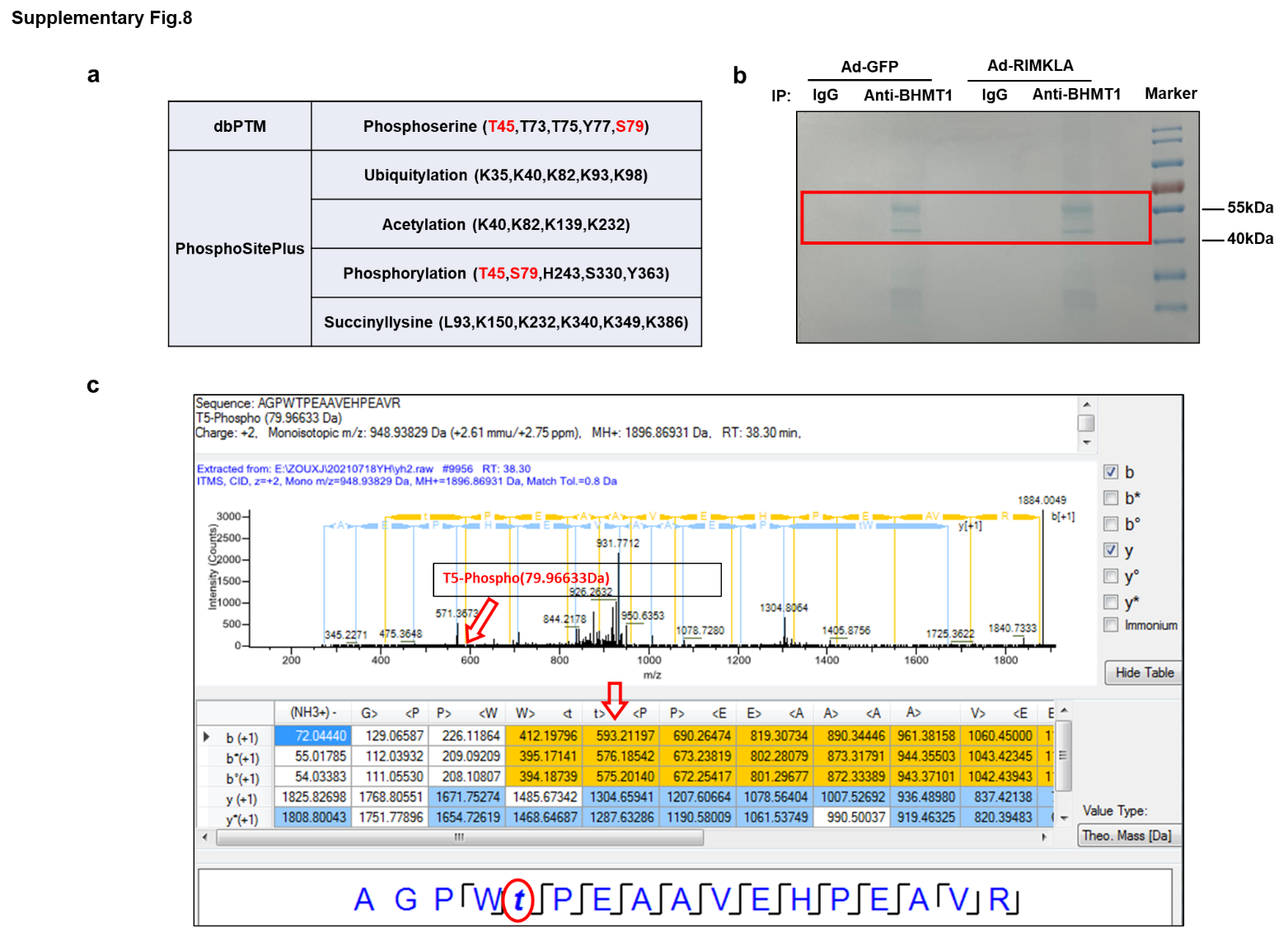


Figure. S8.

**Potential post-translated modifications on BHMT1 protein.**

**a** Prediction of potential post- translational modification (PTM) sites in BHMT1 protein using PhosphoSitePlus v6.6.0.2 (www.phosphosite.org) and dbpIM (www.dbPTM.org). Thr45 and Ser79 sites were accordantly shown in both databases. **b-c** Identification of specific modification site of BHMT1 using Co-IP-MS. Coomassie blue staining of the gel enriched BHMT1 was shown in (**b**). The bands covered the location of BHMT1 (in red circle) were cut out and subjected to MS analysis. Identification for Thr45 phosphorylation of BHMT1 using MS detector in Ad-RIMKLA group when compared with control. The spectrum of Thr45-containing peptide was shown on (**c**), Thr45 site was indicated by the red arrow.


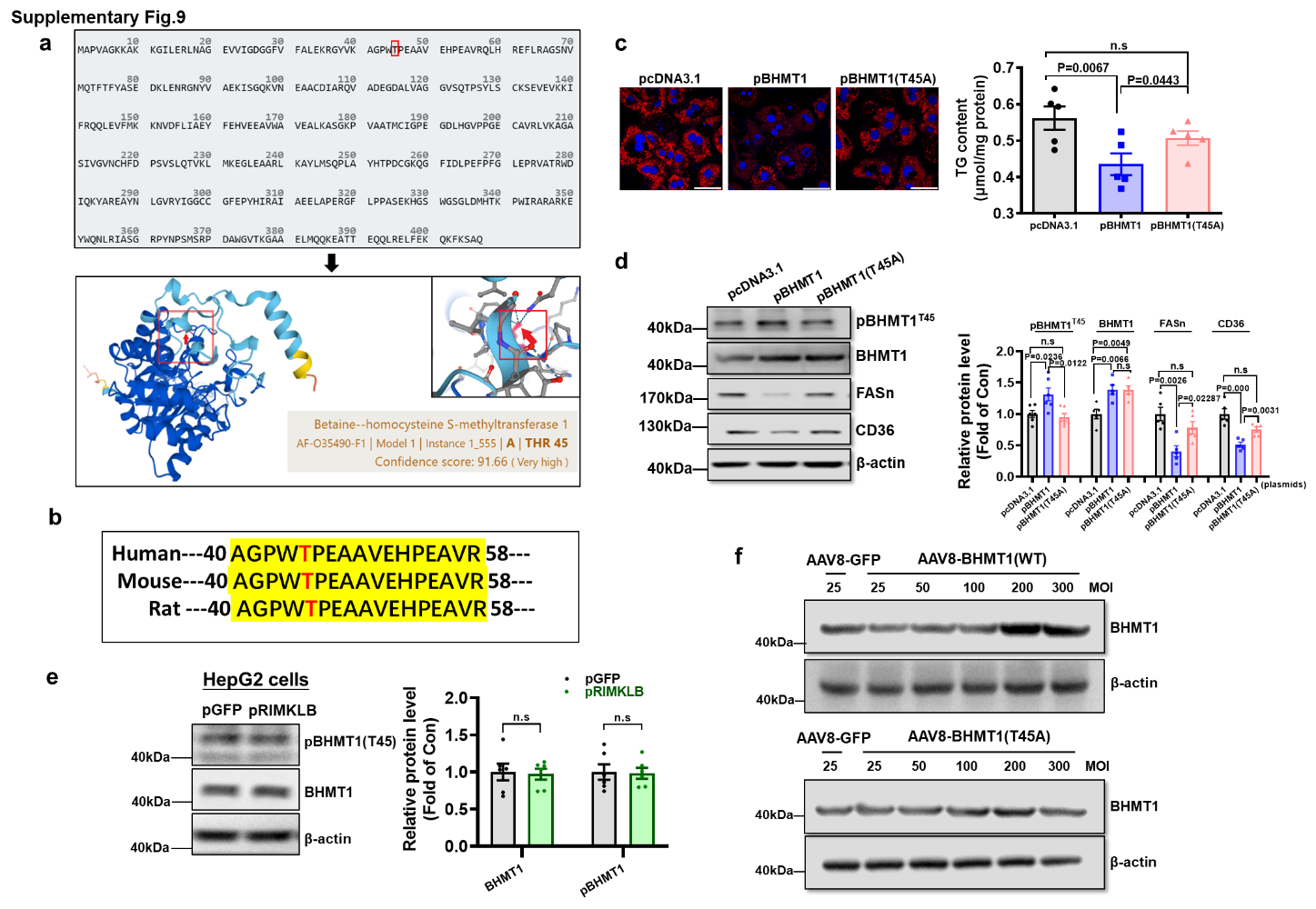


Figure. S9.

**Prediction of Thr45 is important for BHMT1 activity.**

**a** Identification of Thr45 motif on a topology of mouse BHMT1 using Uniprot (www.uniprot.org). **b** Thr45 is highly conserved across human, mouse and rat. **c** Mutation of Thr45 to Ala45 (T45A) abolished BHMT1’s ability to reduce FFAs-induced (0.1 mM oleic acid+0.2 mM palmitic acid) lipid deposition in mouse hepatocytes. Staining images were shown in left panel, and qualitative data of TG in right panel. DAPI (blue), Lipid (red). Scale bar: 50 µm. *n*=5. **d** Mutation of T45A reduced phosphor-BHMT1 (T45) protein levels and abolished BHMT1’s ability to inhibit the expressions of FASn and CD36 in mouse hepatocytes. *n*=5-6. **e** RIMKLB plasmid transfection had little effect on the phosphorylation of BHMT1 at Thr45 site in HepG2 cells. *n*=6. **f** BHMT1 protein levels in mouse hepatocytes administrated by different doses of AAV-BHMT1(WT) or AAV-BHMT1(T45A) for 48 hours. Statistical P values were marked in each panel. P values for (**c-d**) were determined using one-way ANOVA followed by Bonferroni’s post hoc test. P value for **e** was calculated using student’s t-test.


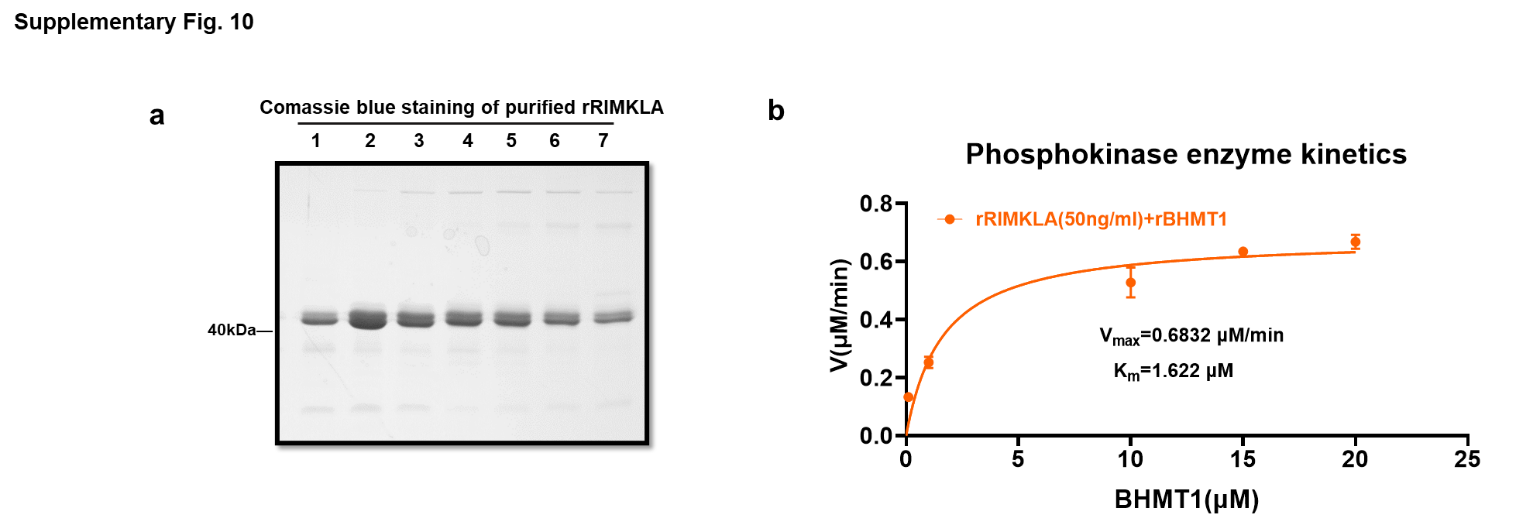


Figure. S10.

**Enzyme kinetics assay of RIMKLA phosphorylates on BHMT1.**

**a** Mouse *Rimkla* gene (NM_177572.4) was synthesized and cloned to pET-28a vector to construct pET-28a-Rimkla-His recombinant plasmid, which was then expressed in *E. coli* BL21(DE3) to obtain the fusion protein (wzbio Biosciences Inc., China). His-tag fusion protein in the protein extract supernatant was captured by Ni-NTA agarose resin. The ion exchange chromatography was used for intermediate purification. Then the purified recombinant RIMKLA (rRIMKLA) was replaced into desalting buffer (20 mM Tris, 100 mM KCl, 1 mM DTT, 10 mM MgCl_2_, 1% Tween 20, 5% GLY, pH=8.0) by desalting column (Sephadex^TM^ G-25 Fine, cross-linked dextran, Cytiva). Line 1-7: Comassie blue staining of rRIMKLA after desalination replacement. **b** BHMT1 recombinant protein (0-20 μM) was added as substrate and reacted with 50 ng/mL rRIMKLA protein at room temperature for 15 minutes to 1 hour, and the amount of ADP produced by RIMKLA-BHMT1 kinase reaction was detected. The kinetic parameters of RIMKLA on BHMT1 were calculated and fitted to the Michaelis-Menten equation by GraphPad Prism software to derive the apparent V_max_ and K_m_. The calculated K_m_ was 1.622 μM, and V_max_ was 0.6832 μM/min. *n*=3.


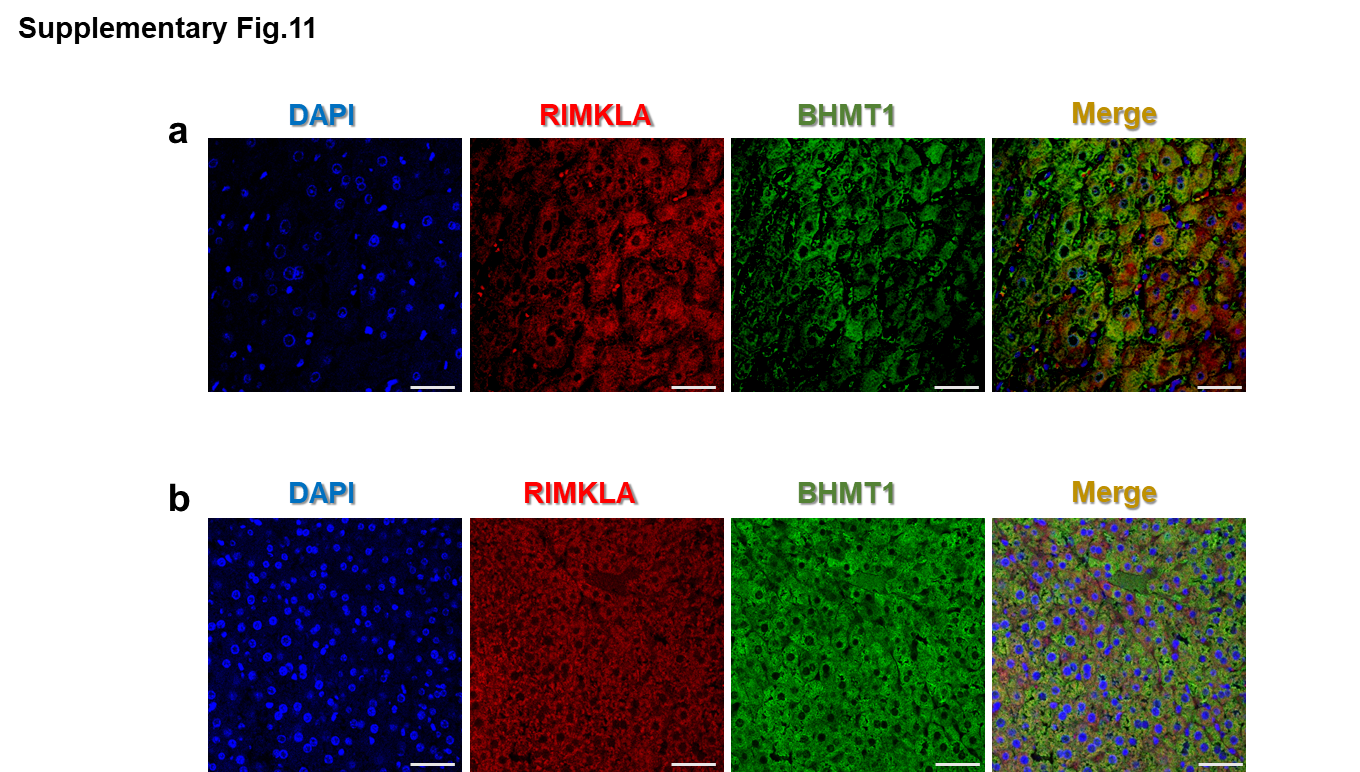


Figure. S11.

**RIMKLA is colocalized with BHMT1 in human and mouse liver.**

**a-b** RIMKLA and BHMT1 are colocalized in human (**a**) and mouse livers (**b**). Representative images were chosen from three independent liver samples. DAPI (blue), RIMKLA (red), BHMT1 (green), Merge (yellow). Scale bar: 50 µm.


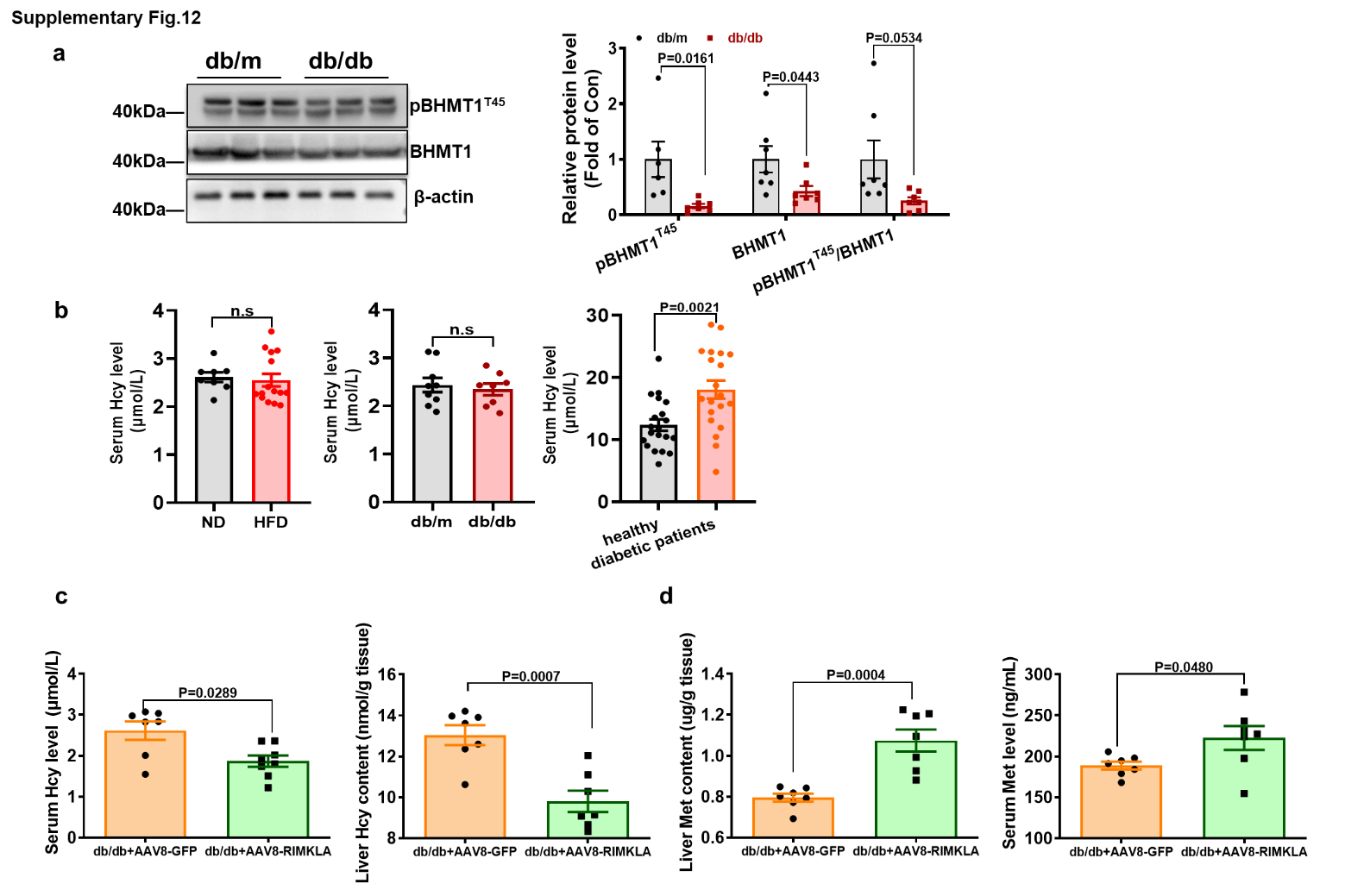


Figure. S12.

**RIMKLA overexpression on hepatic and serum Hcy and Met levels in db/db mice.**

**a** The levels of pBHMT^T45^, BHMT1 and pBHMT1/BHMT1 ratio were reduced in livers of diabetic mice. *n*=6-7. **b** Serum Hcy levels in mice and human compared with indicated controls. *n*=8-9 for the left panel, *n*=8-15 for the middle panel. Hcy content was increased in serum of patients with diabetes versus healthy groups (right panel). *n*=20. **c-d** Hcy and Met levels in serum or livers of db/db mice after AAV8-GFP or AAV8-RIMKLA infection for 16 weeks. *n*=7-8. Statistical P values were marked in each panel. P values for all figures were determined by student’s t-test.


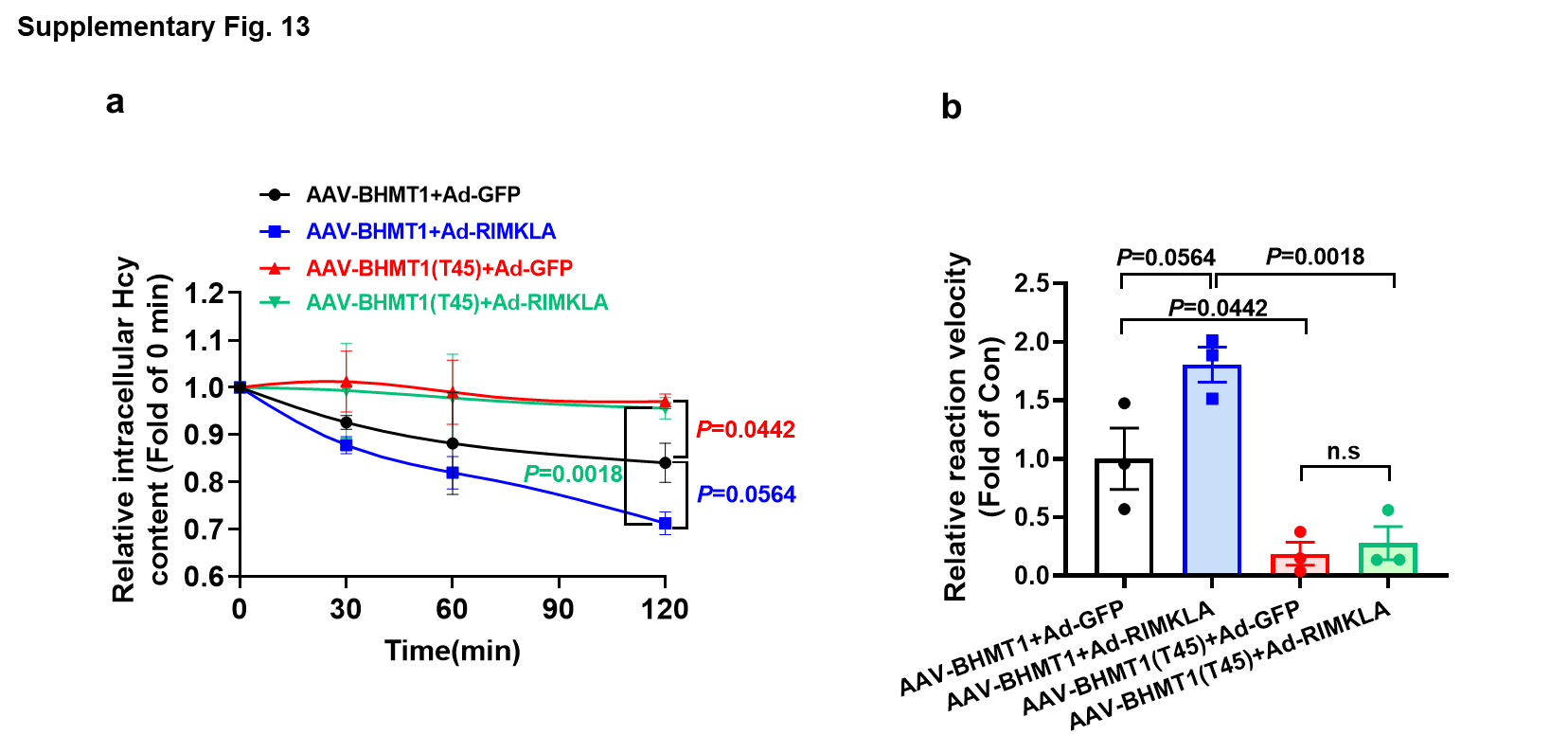
Figure. S13.

**Enzyme kinetics assay of phosphorylated and dephosphorylated BHMT1.**

Mouse hepatocytes were infected with wild-type or mutant AAV8-BHMT1 for 48 hours, and then infected with Ad-GFP or Ad-RIMKLA for 24 hours. After treatment, DL-Hcy (500 μM) was added to each group at different times (0-120 min) before cell collection. **a** The relative intracellular Hcy content was detected to represent the kinetics of phosphorylated and dephosphorylated BHMT1 on Hcy metabolism. *n*=3. **b** The average reaction velocity was compared with different groups. *n*=3. Statistical P values were marked in each panel. All P values were analyzed by two-way ANOVA with Tukey’s tests.


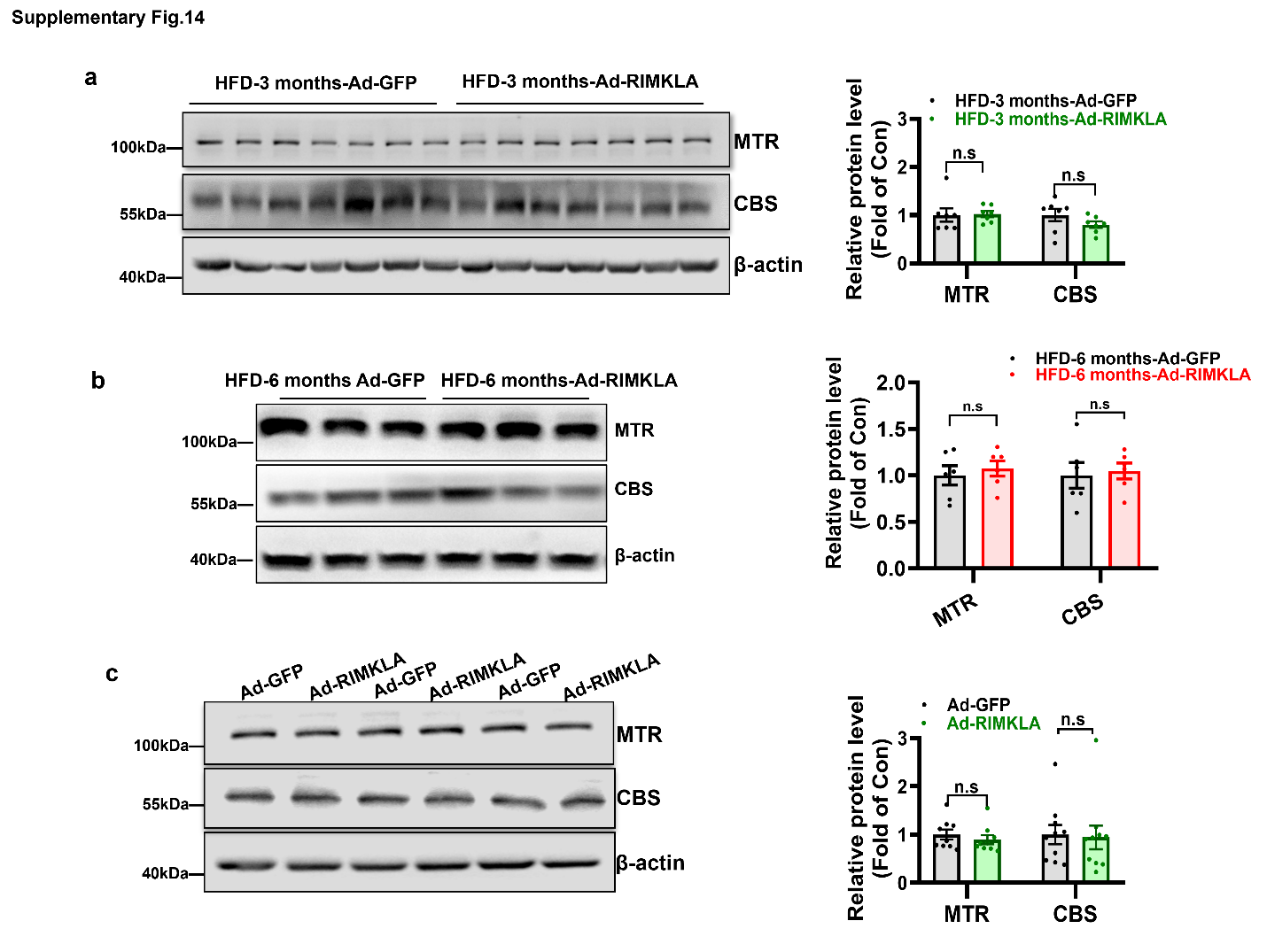


Figure. S14.

**RIMKLA overexpression has little effect on MTR and CBS protein levels in HFD mice and mouse hepatocytes.**

**a-c** RIMKLA overexpression on proteins levels of MTR and CBS in livers of mice fed on HFD for 3 months (**a**) or for 6 months (**b**), and cultured mouse hepatocytes (**c**). *n*=6-10. MTR: 5-methyltetrahydrofolate-homocysteine methyltransferase (also called methionine synthase). CBS: cystathionine β-synthase. Representative gel images were shown on left panel, and quantitative data shown on right panel. P values were analyzed by student’s t-test, n.s means statistically insignificance.


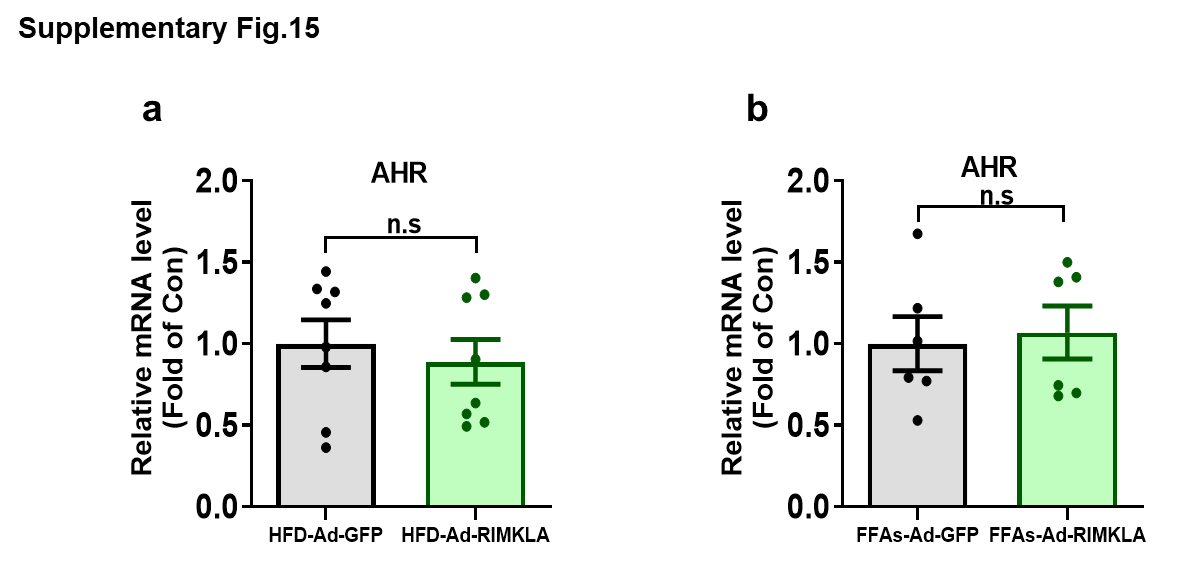


Figure. S15.

**RIMKLA overexpression has little effect on AHR mRNA levels in livers of HFD mice or cultured mouse hepatocytes.**

**a** mRNA level of AHR in livers of HFD mice injected with Ad-GFP or Ad-RIMKLA via tail veins. *n*=8. **b** mRNA level of AHR in mouse hepatocytes treated with Ad-GFP or Ad-RIMKLA for 24 hours in the presence of FFAs. *n*=6. FFAs, free fatty acids (0.1 mM oleic acid+0.2 mM palmitic acid). AHR, aryl hydrocarbon receptor. Student’s t-test was used to compare the difference between groups for (**a, b**), n.s, no significant difference.


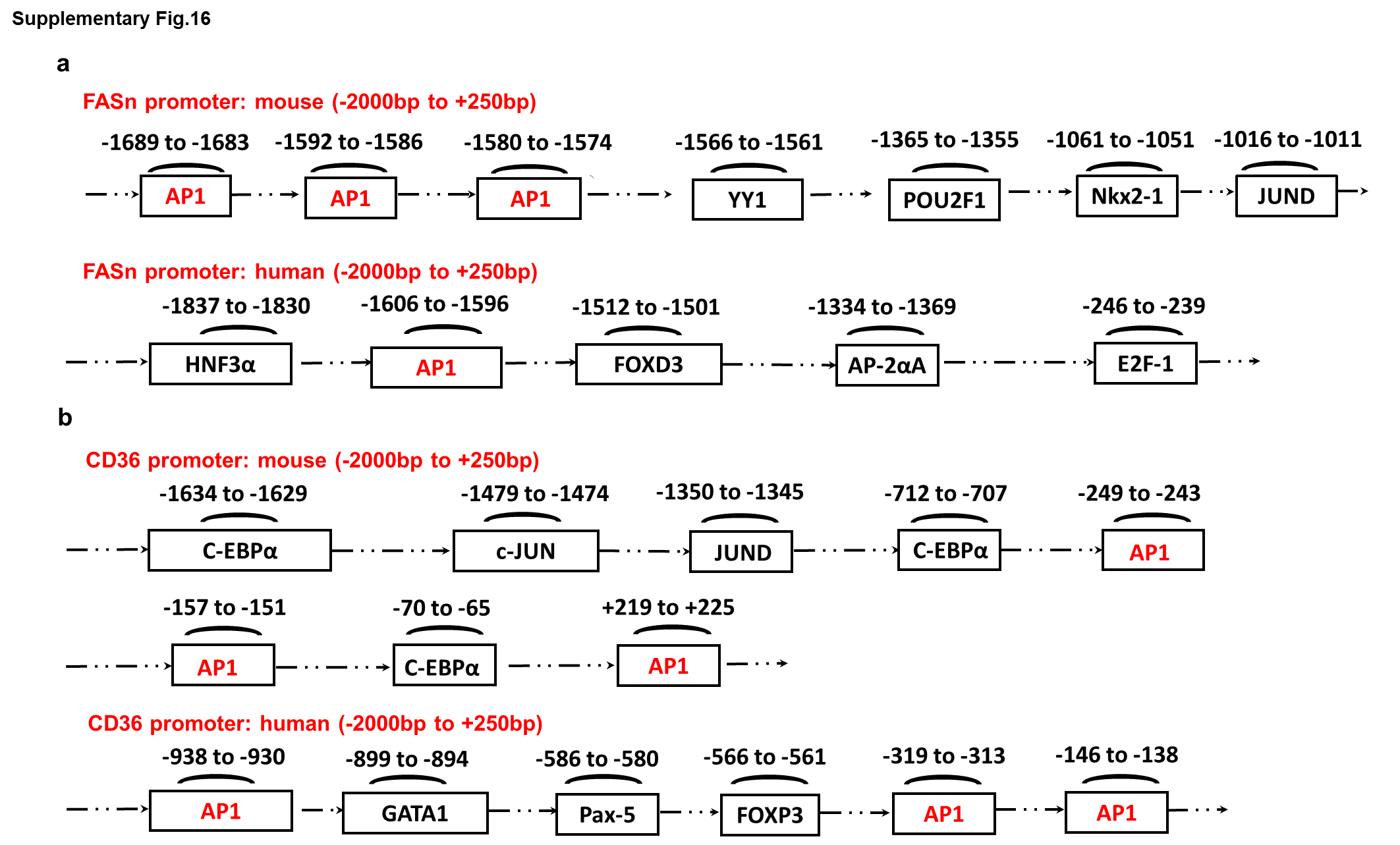


Figure. S16.

**Prediction of potential binding sites for the transcriptional factors in the promoter regions of mouse/human FASn and CD36 genes.**

**a-b** Potential transcriptional factor binding sites were predicted in the promoter regions (upstream -2000bp to +250bp) of mouse/human FASn (**a**) or CD36 (**b**) genes. The prediction was analyzed using the database and softwares in the website: http://www.genome.ucsc.edu/, http://www.gene-regulation.com/pub/databases.html/ and https://alggen.lsi.upc.es/.


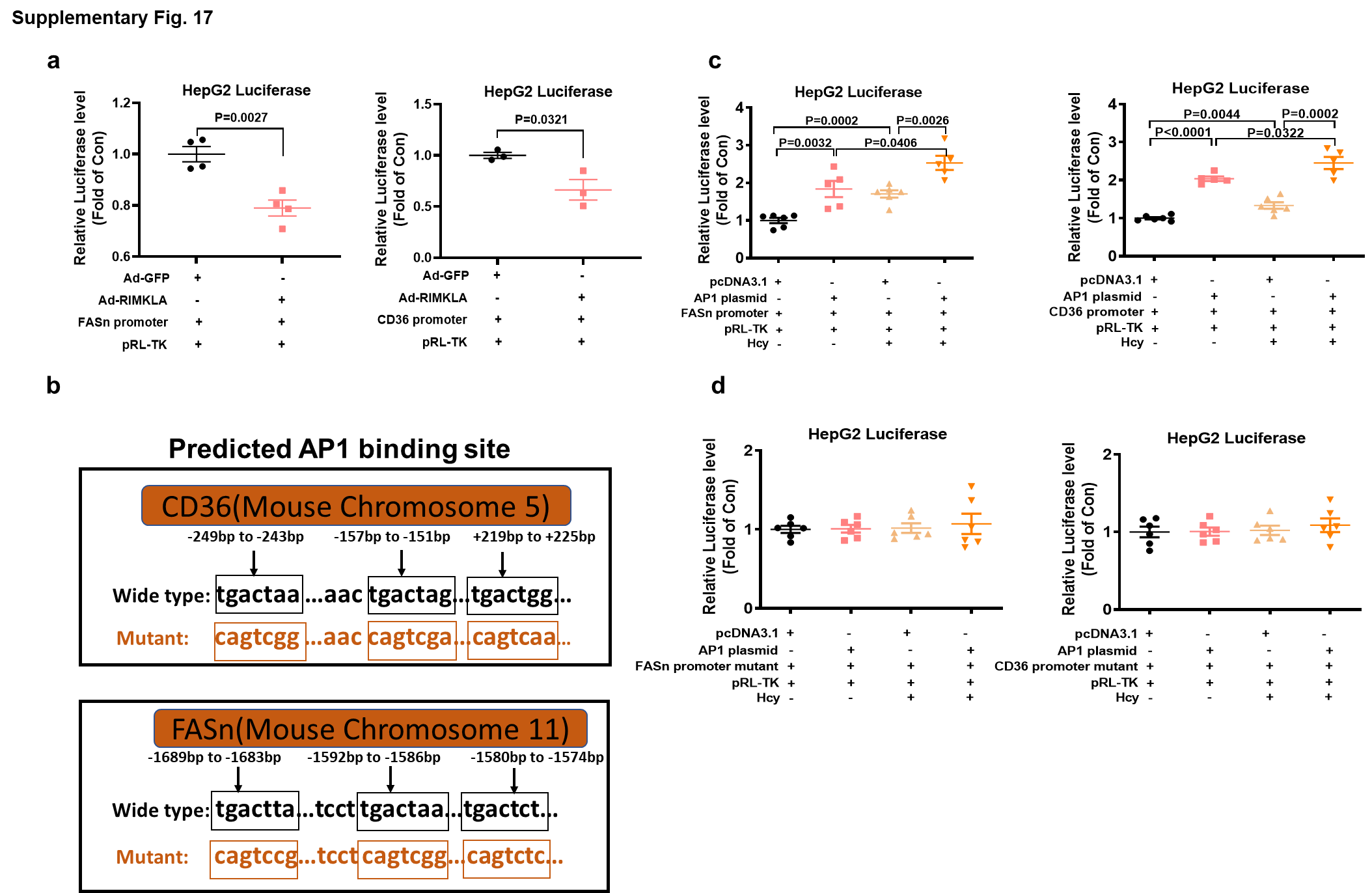


Figure. S17.

**The effects of RIMKLA overexpression and Hcy on the transcriptional activities of FASn and CD36 promoters.**

**a** RIMKLA overexpression inhibited the promoter activities of FASn (left panel) and CD36 (right panel) genes. Dual-luciferase reporter assays were performed in HepG2 cells. *n*=3-4. **b** Prediction of potential AP1 binding sites on the promoter regions of mouse CD36 (upper panel) and FASn (lower panel) genes. Potential binding sites for transcription were analyzed using the softwares in websites: http://www.genome.ucsc.edu/, http://gene-regulation.com/pub/programs.html and https://alggen.lsi.upc.es/. Based on the prediction, the wild type mouse FASn promoter region (-2000bp to +250bp) and mouse CD36 region (-2000bp to +250bp) were cloned into pGL3 vectors (Generay Biotechnology). At the same time, the mutant promoter regions with the mutations of all potential AP1 binding sites in FASn and CD36 promoters were also constructed. **c** AP1 overexpression and Hcy treatment activated the promoter activities of FASn (left panel) and CD36 (right panel) genes. Dual-luciferase reporter assays were performed in HepG2 cells. AP1 and Hcy activated the promoter activities, and Hcy further augmented AP1’s activation on the promoter activities. *n*=5-6. **d** AP1 and Hcy failed to activate the activities of mutant promoters of FASn (left panel) and CD36 (right panel) genes. *n*=6. Statistical P values were marked in each panel. P values for (**a**) was calculated by student’s t-test, for (**c-d**) were analyzed using one-way ANOVA followed by Bonferroni’s post hoc tests.


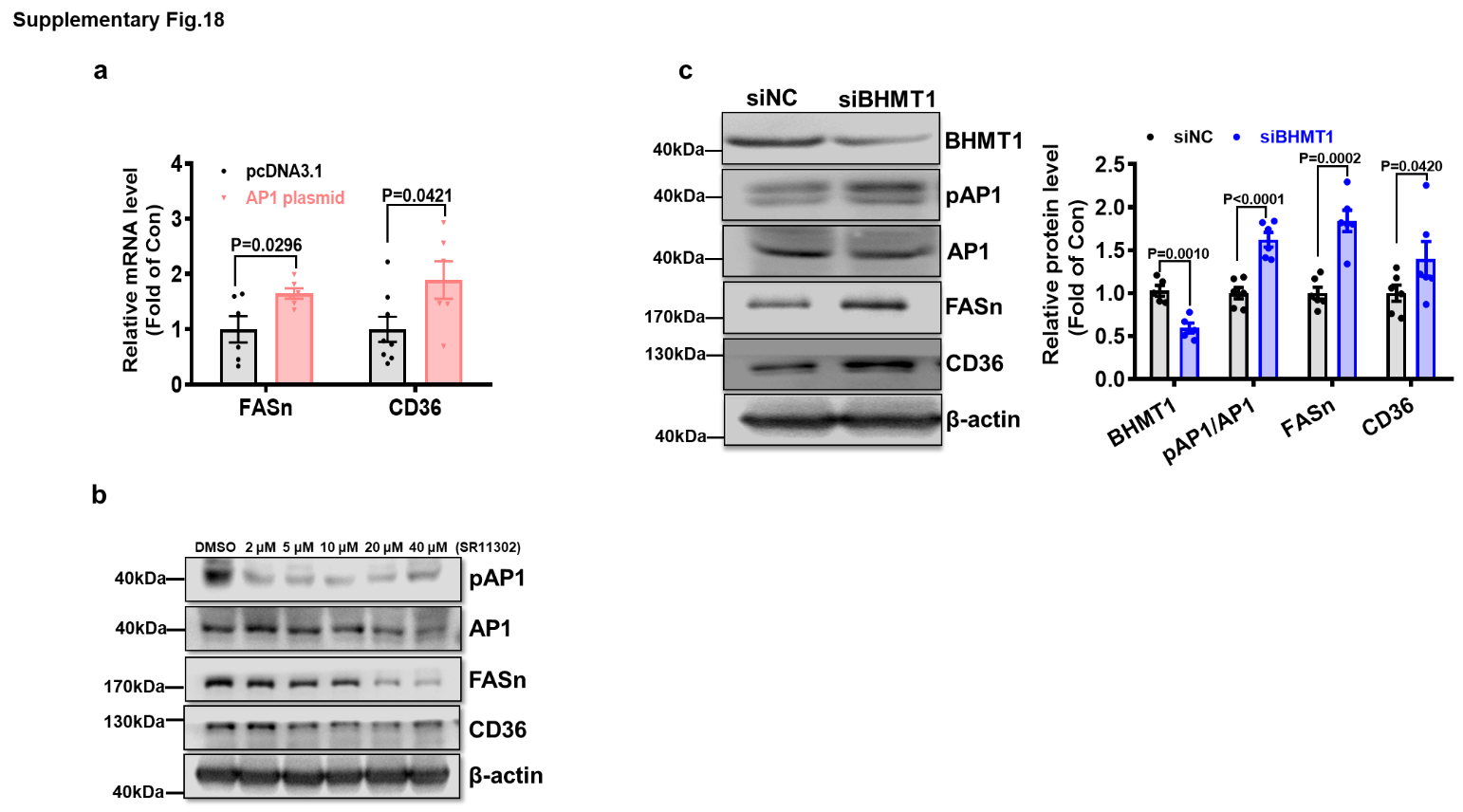


Figure. S18.

**CD36 and FASn expressions are suppressed by AP1 inhibitor and enhanced after BHMT1 silencing.**

**a** FASn and CD36 mRNA levels in mouse hepatocytes after transfected with pcDNA3.1 or AP1 plasmid for 24 hours. *n*=6-8. **b** Phosphorylated AP1, AP1, FASn and CD36 protein levels after treatment with different concentrations of AP1 inhibitor (2, 5, 10, 20, 40 µM) for 24 hours in mouse hepatocytes. DMSO served as control. **c** BHMT1 silencing increased the protein expressions of phosphorylated AP1, CD36 and FASn in mouse hepatocytes. Cells were transfected with siBHMT1 (100 nM) or control siRNA for 48 hours. *n*=5-6. Statistical P values were marked in each panel. Student’s t-test was used to analyze the statistical difference of (**a, c**).


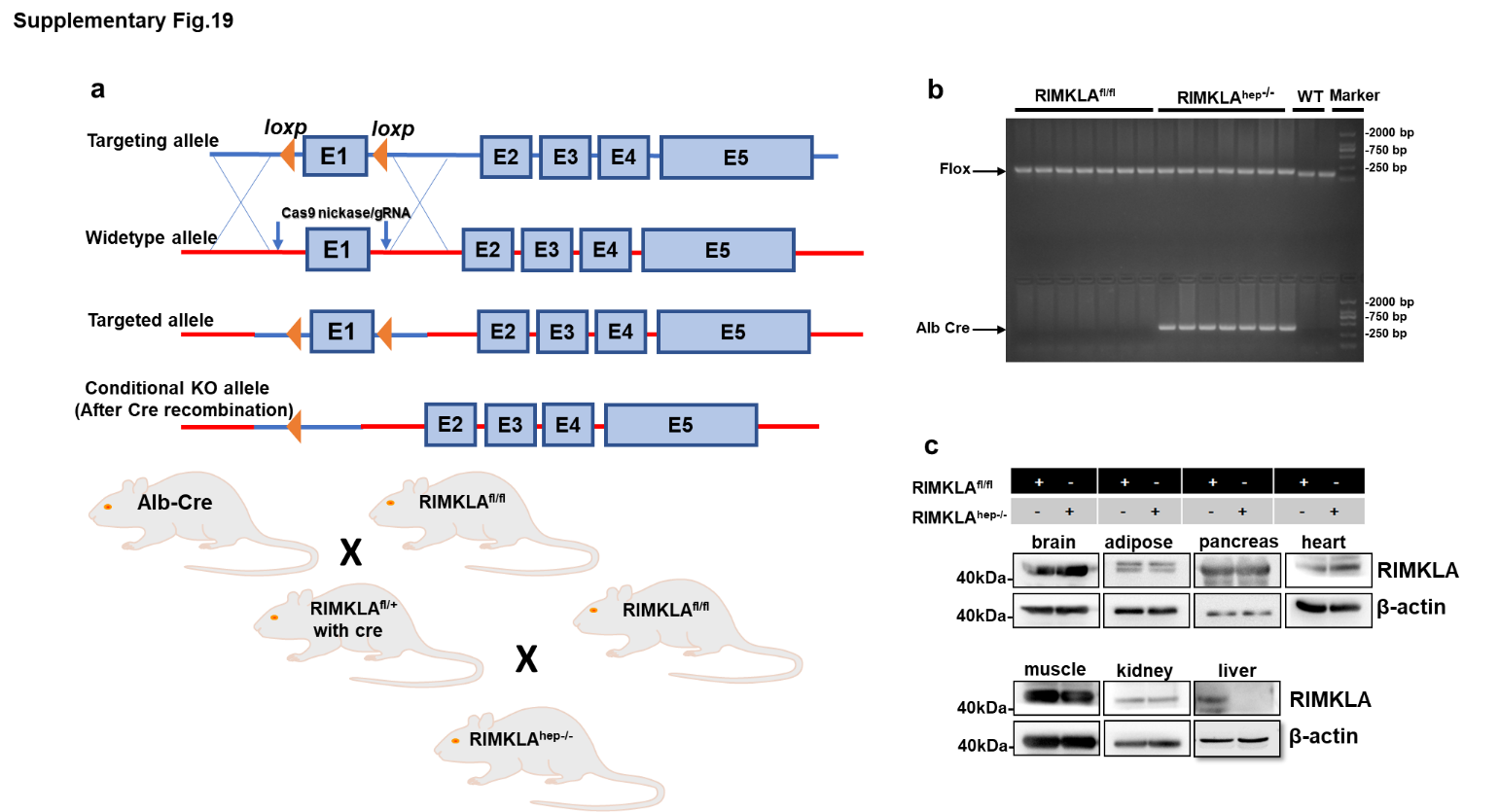


Figure. S19.

**Generation and characterization of hepatocyte-specific RIMKLA knockout mice.**

**a** RIMKL^fl/fl^ mice were generated after two Loxp sites inserted into the two ends of exon 1. Hepatocyte-specific RIMLA deletion mice (RIMKLA^hep-/-^) were generated by crossing RIMKLA^fl/fl^ mice with Alb-Cre mice. **b** Genomic DNA analysis for RIMKLA^fl/fl^ and RIMKLA^hep-/-^ mice. **c** Representative blotting images for RIMKLA expression in various tissues of RIMKLA^fl/fl^ and RIMKLA^hep-/-^ mice.


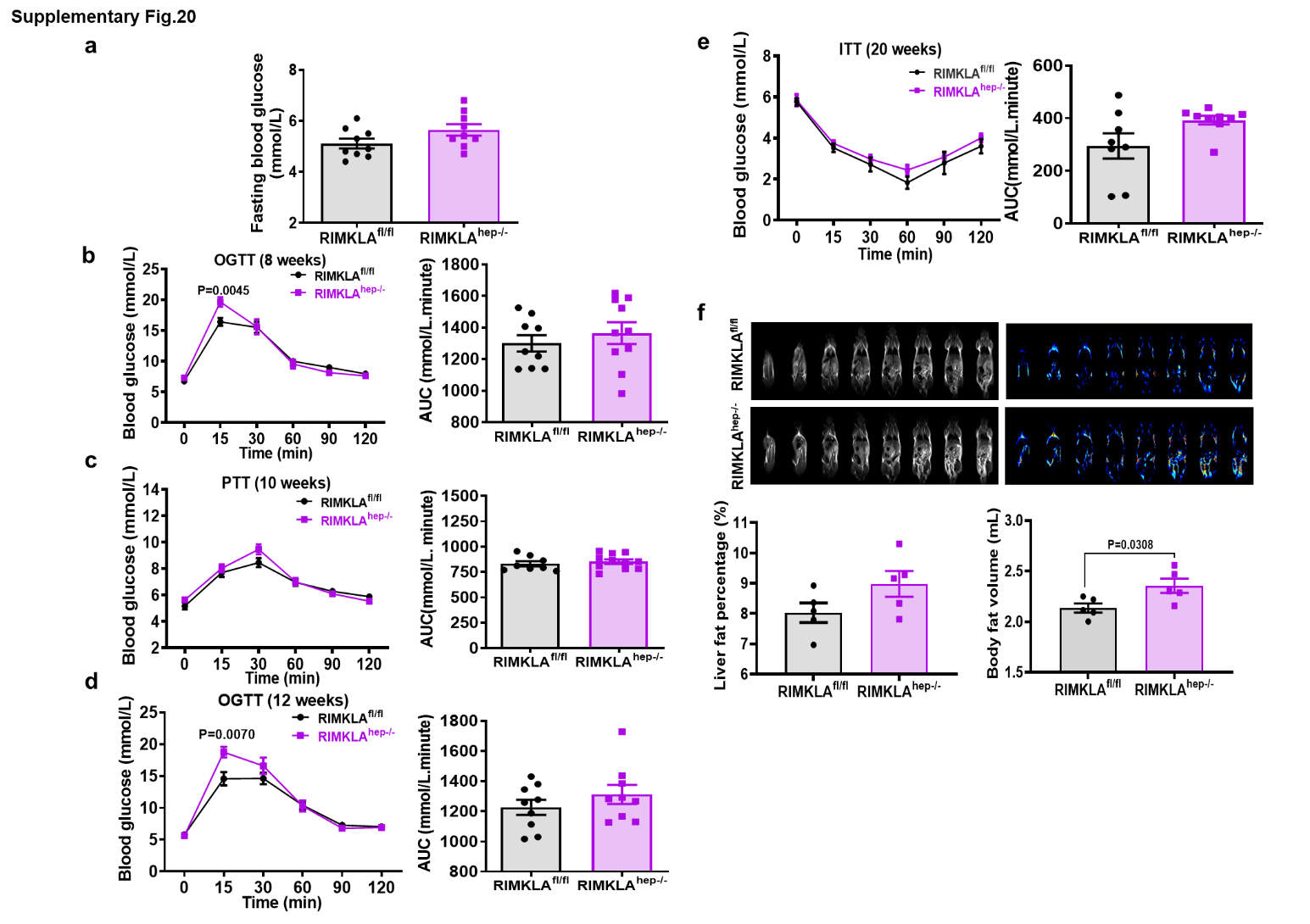


Figure. S20.

**Metabolic phenotypes of RIMKLA^hep-/-^ mice fed on normal diet.**

**a** Fasting blood glucose of 8-week-old male RIMKLA^fl/fl^ and RIMKLA^hep-/-^ mice. *n*=9. **b-e** Dynamic analyses of glucose metabolic phenotypes by OGTT, PTT and ITT at different ages. OGTT were performed on these mice at the age of 8 weeks and 12 weeks, PTT and ITT assays were performed when the mice at 10 weeks and 20 weeks old respectively. *n*=8-11. Areas under curve (AUC) were shown on right panels for (**b-e**). **f** MRI scanning for the two groups of mice at the age of 10 weeks, hepatic lipid and body lipid signals were imaged and analyzed. Representative images were shown on upper panel, and quantitative data on lower panel. *n*=5. Statistical P values were marked in each panel. Two-way ANOVA with Tukey’s tests were performed to analyze the statistical significance of left panels of (**b-e**), student’s t-tests were used to analyze the difference of (**a, f**) and AUC data for (**b-e**).


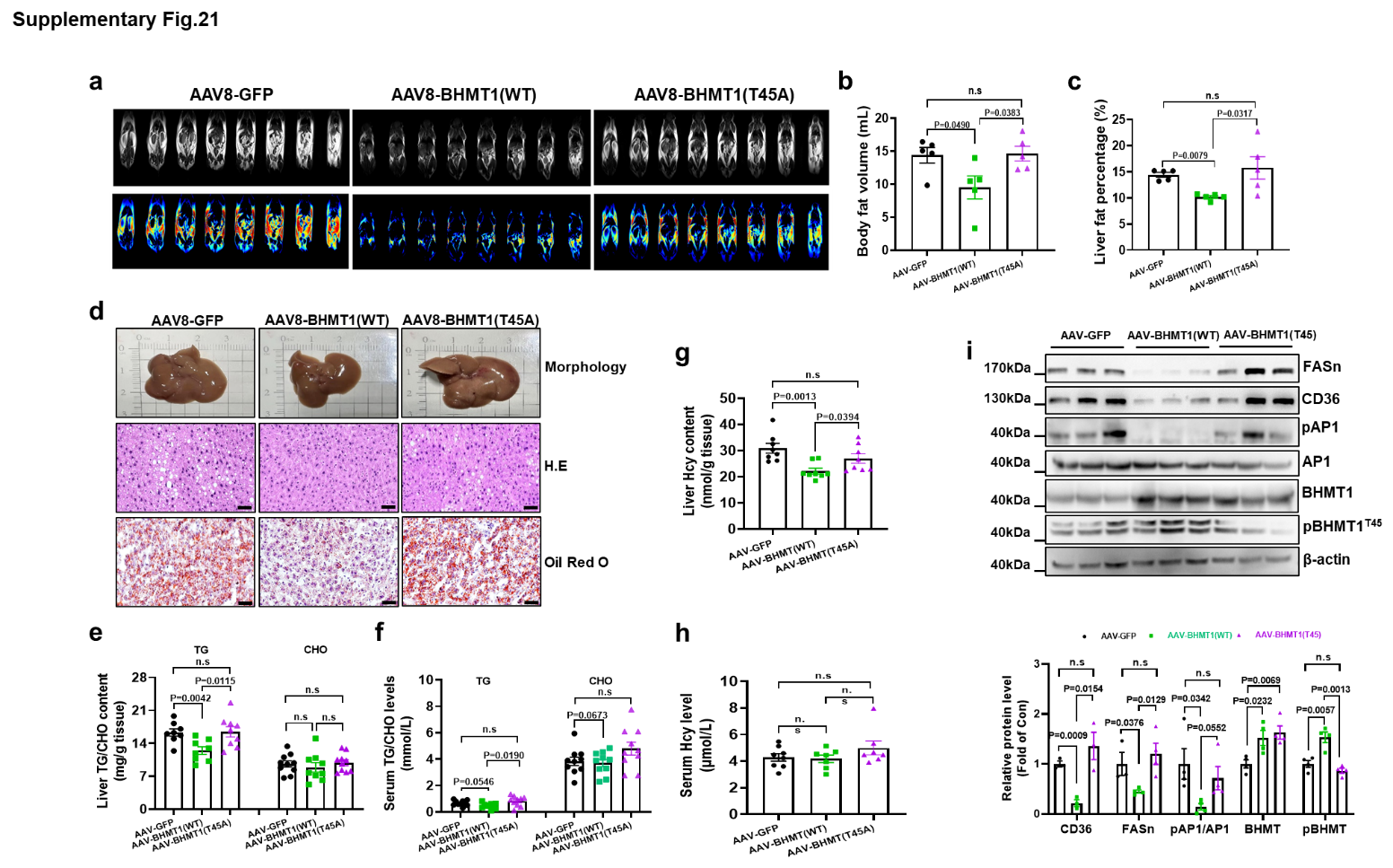


Figure. S21.

**Hepatic overexpression of mutant BHMT1 fails to reduce Hcy and lipid content in mouse livers.**

Three groups of 8-week-old male C57BL/6 mice were transduced with AAV8-GFP, AAV8-BHMT1 and AAV-BHMT1-T45A, respectively, followed by feeding on HFD for 12 weeks. **a-c** MRI analysis of lipid deposition in mice. Representative MRI images were shown in panel **a**, and quantitative data shown in panels **b** and **c**. *n*=5. **d** Morphological observation, HE and Oil Red O staining of mouse livers. Scale bar: 50 μm. **e-f** Determination of hepatic (**e**) and serum (**f**) TG and CHO levels of mice. *n*=8-10. **g-h** Determination of hepatic (**g**) and serum (**h**) Hcy content of mice. *n*=7-8. **i** Determination of metabolic gene expression in mouse livers. Representative gel images were shown in upper panel, and quantitative data shown in lower panel. *n*=3-4. Statistical P values were marked in each panel. P values for (**b-c,e-i**) were determined using one-way ANOVA followed by Bonferroni’s post hoc test.


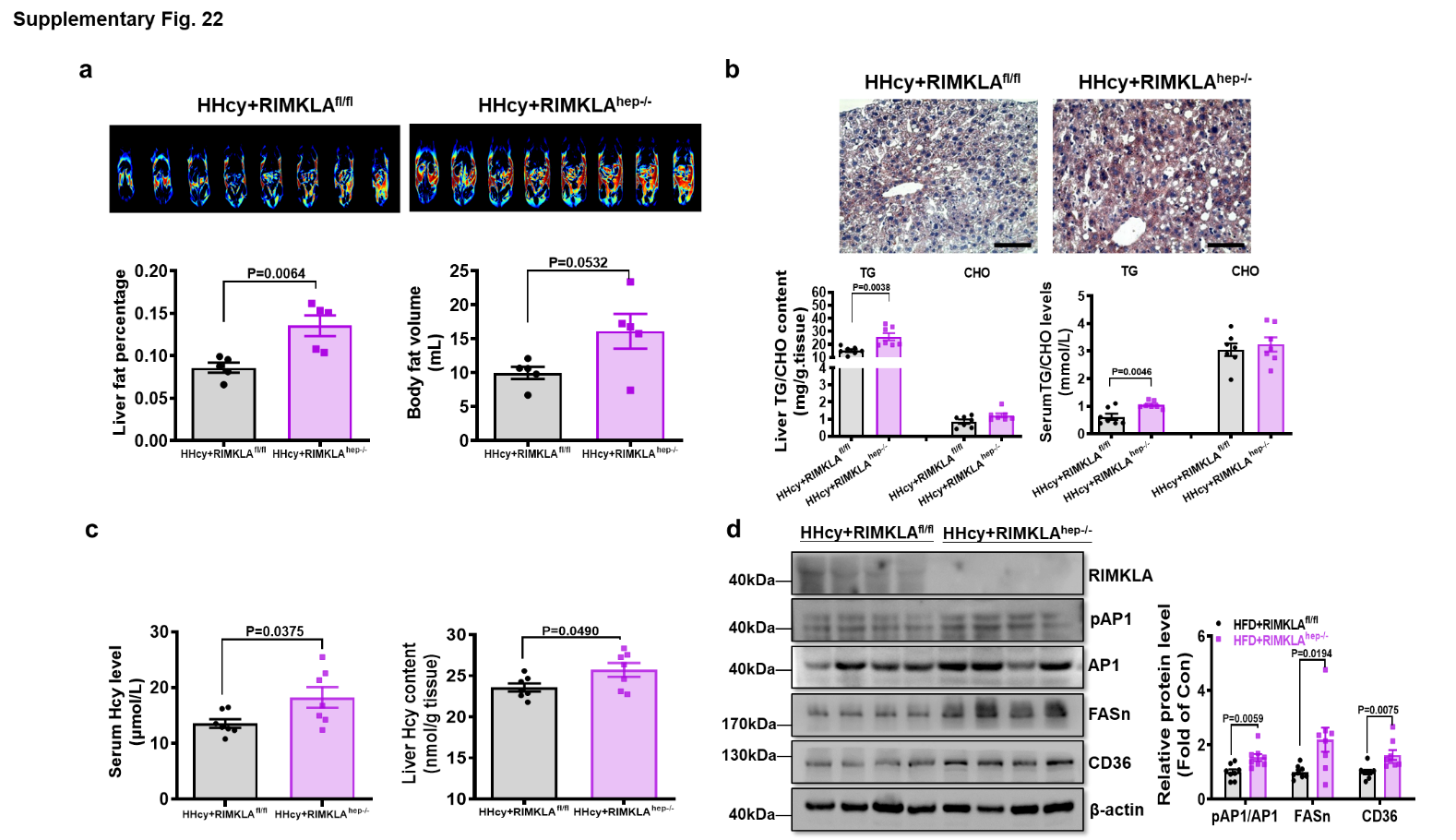


Figure. S22.

**Hepatic RIMKLA deletion exaggerates Hcy-promoted lipid accumulation and HHcy in mice.**

**a** MRI scanning and quantification of hepatic and body fat for HHcy+RIMKLA^fl/fl^ and HHcy+RIMKLA^hep-/-^ mice. The upper images were the representatives of 10 animals. *n*=5 in each group. **b** RIMKLA deletion aggravated Hcy-induced hepatic lipid deposition. Representative Oil Red O staining images were shown on upper panel, and quantification of liver and serum TG/CHO levels shown in lower panels. *n*=7. Scale bar: 20 μm. **c** Serum and hepatic Hcy levels in the indicated groups. *n*=7. **d** Hepatic pAP1, AP1, CD36 and FASn expressions in RIMKLA^fl/fl^ and RIMKLA^hep-/-^ mice fed on Hcy. *n*=8*.* HHcy mouse model was developed in RIMKLA^fl/fl^ and RIMKLA^hep-/-^ mice fed with 1.8 g/L DL-Hcy in drinking water for 8 weeks. P values were marked in each panel. P values for (**a-d**) were determined by student’s t-tests.

Table S1.

**Genes predicted by GIC**

| Genes | GIC score | Publications in Zeng et al (2018) | Publications in PubMed |
| --- | --- | --- | --- |
| SOGA3 | 0.911353655 | 7 | 0 |
| ANKRD33B | 0.808982278 | 3 | 1 |
| ZNF316 | 0.837552052 | 7 | 2 |
| RIMKLA | 0.802436188 | 8 | 3 |
| ALG10B | 0.800478431 | 8 | 3 |
| CLVS2 | 0.845798113 | 5 | 4 |
| NKX1-1 | 0.837484631 | 6 | 4 |
| ZC3H6 | 0.828457161 | 6 | 4 |
| DNAH3 | 0.902935178 | 10 | 4 |
| KCNG2 | 0.819232451 | 4 | 5 |
| PTAR1 | 0.817920398 | 7 | 6 |
| NBPF20 | 0.957558862 | 1 | 7 |
| PLEKHG4B | 0.858079295 | 6 | 7 |
| TMEM132B | 0.811154019 | 8 | 7 |
| SP9 | 0.838885357 | 1 | 8 |
| CSRNP3 | 0.839605907 | 9 | 8 |
| LONRF2 | 0.921144045 | 8 | 9 |
| BAHCC1 | 0.883470483 | 10 | 9 |
| ZNF704 | 0.921698551 | 5 | 10 |
| C1QTNF4 | 0.871399435 | 5 | 11 |
| ISLR2 | 0.822499942 | 6 | 11 |
| IGFN1 | 0.816519893 | 5 | 12 |
| USP31 | 0.849209897 | 8 | 12 |
| DNAH2 | 0.923955039 | 7 | 14 |
| HMCN2 | 0.959188326 | 8 | 15 |
| DNAH7 | 0.906731725 | 9 | 17 |
| PKHD1L1 | 0.892419545 | 6 | 20 |
| MUC12 | 0.941215161 | 10 | 33 |
| DNAH17 | 0.945610129 | 9 | 42 |
| FAT2 | 0.89729088 | 10 | 47 |

Table S2.

**Mass spectrometry data for potential RIMKLA interacting molecules.**

| Accession | Description | Score | Coverage | # Proteins | # Unique Peptides | # Peptides | # PSMs | # AAs | MW [kDa] | calc. pI |
| --- | --- | --- | --- | --- | --- | --- | --- | --- | --- | --- |
| A0A0A6YXN4 | Protein Ighv1-18 (Fragment) OS=Mus musculus GN=Ighv1-18 PE=4 SV=1 - [A0A0A6YXN4_MOUSE] | 283.49 | 43.59 | 3 | 4 | 5 | 109 | 117 | 12.9 | 7.94 |
| P01863 | Ig gamma-2A chain C region, A allele OS=Mus musculus GN=Ighg PE=1 SV=1 - [GCAA_MOUSE] | 157.65 | 48.79 | 3 | 11 | 13 | 56 | 330 | 36.4 | 7.40 |
| O35490 | Betaine--homocysteine S-methyltransferase 1 OS=Mus musculus GN=Bhmt PE=1 SV=1 - [BHMT1_MOUSE] | 96.40 | 50.37 | 2 | 19 | 19 | 35 | 407 | 45.0 | 7.90 |
| P50446 | Keratin, type II cytoskeletal 6A OS=Mus musculus GN=Krt6a PE=1 SV=3 - [K2C6A_MOUSE] | 93.52 | 27.12 | 7 | 8 | 23 | 38 | 553 | 59.3 | 7.94 |
| Q8QZT1 | Acetyl-CoA acetyltransferase, mitochondrial OS=Mus musculus GN=Acat1 PE=1 SV=1 - [THIL_MOUSE] | 88.95 | 41.04 | 1 | 11 | 11 | 26 | 424 | 44.8 | 8.51 |
| A0A075B5P3 | Protein Ighg2b (Fragment) OS=Mus musculus GN=Ighg2b PE=4 SV=1 - [A0A075B5P3_MOUSE] | 78.53 | 30.36 | 4 | 9 | 10 | 28 | 336 | 36.7 | 7.36 |
| Q61781 | Keratin, type I cytoskeletal 14 OS=Mus musculus GN=Krt14 PE=1 SV=2 - [K1C14_MOUSE] | 78.23 | 26.65 | 13 | 1 | 13 | 31 | 484 | 52.8 | 5.17 |
| Q922U2 | Keratin, type II cytoskeletal 5 OS=Mus musculus GN=Krt5 PE=1 SV=1 - [K2C5_MOUSE] | 77.09 | 23.79 | 3 | 5 | 22 | 33 | 580 | 61.7 | 7.75 |
| P22315 | Ferrochelatase, mitochondrial OS=Mus musculus GN=Fech PE=1 SV=2 - [HEMH_MOUSE] | 76.35 | 50.48 | 2 | 19 | 19 | 30 | 420 | 47.1 | 8.91 |
| A0A0A6YVW3 | Protein Ighv1-23 (Fragment) OS=Mus musculus GN=Ighv1-23 PE=4 SV=1 - [A0A0A6YVW3_MOUSE] | 69.73 | 20.51 | 3 | 1 | 2 | 36 | 117 | 12.9 | 6.51 |
| Q9Z2K1 | Keratin, type I cytoskeletal 16 OS=Mus musculus GN=Krt16 PE=1 SV=3 - [K1C16_MOUSE] | 68.46 | 20.04 | 13 | 5 | 10 | 30 | 469 | 51.6 | 5.20 |
| P04104 | Keratin, type II cytoskeletal 1 OS=Mus musculus GN=Krt1 PE=1 SV=4 - [K2C1_MOUSE] | 68.08 | 6.44 | 1 | 2 | 7 | 25 | 637 | 65.6 | 8.15 |
| Q8BWT1 | 3-ketoacyl-CoA thiolase, mitochondrial OS=Mus musculus GN=Acaa2 PE=1 SV=3 - [THIM_MOUSE] | 62.40 | 48.36 | 1 | 15 | 15 | 21 | 397 | 41.8 | 8.09 |
| Q02257 | Junction plakoglobin OS=Mus musculus GN=Jup PE=1 SV=3 - [PLAK_MOUSE] | 59.43 | 23.36 | 1 | 13 | 13 | 21 | 745 | 81.7 | 6.14 |
| Q6IFX2 | Keratin, type I cytoskeletal 42 OS=Mus musculus GN=Krt42 PE=1 SV=1 - [K1C42_MOUSE] | 56.57 | 19.25 | 11 | 1 | 9 | 22 | 452 | 50.1 | 5.16 |
| P16460 | Argininosuccinate synthase OS=Mus musculus GN=Ass1 PE=1 SV=1 - [ASSY_MOUSE] | 56.08 | 27.91 | 2 | 13 | 13 | 21 | 412 | 46.6 | 8.22 |
| Q9JHJ0 | Tropomodulin-3 OS=Mus musculus GN=Tmod3 PE=2 SV=1 - [TMOD3_MOUSE] | 54.85 | 42.05 | 3 | 16 | 16 | 24 | 352 | 39.5 | 5.14 |
| Q3TTY5 | Keratin, type II cytoskeletal 2 epidermal OS=Mus musculus GN=Krt2 PE=1 SV=1 - [K22E_MOUSE] | 51.42 | 10.89 | 3 | 7 | 14 | 23 | 707 | 70.9 | 8.06 |

| Treatment (24 hours) | Protein Group Accessions | Sequence | # PSMs | Modifications |
| --- | --- | --- | --- | --- |
| Ad-GFP | O35490 | LKAYLmSQPLAYHTPDcGK | 2 | M6(Oxidation); C17(Carbamidomethyl) |
|  | O35490 | AGSNVmQTFTFYASEDKLENR | 4 | M6(Oxidation) |
|  | O35490 | AYLmSQPLAYHTPDcGK | 1 | M4(Oxidation); C15(Carbamidomethyl) |
|  | O35490 | LmKEGLEAAR | 2 | M2(Oxidation) |
|  | O35490 | IASGRPYNPSmSRPDAWGVTK | 1 | M11(Oxidation) |
|  | O35490 | ASGKPVAATmcIGPEGDLHGVPPGEcAVR | 2 | M10(Oxidation); C11(Carbamidomethyl); C26(Carbamidomethyl) |
|  | O35490 | HGSWGSGLDmHTKPWIR | 1 | M10(Oxidation) |
|  | O35490 | IFRQQLEVFmKK | 1 | M10(Oxidation) |
|  | O35490 | VNEAAcDIAR | 1 | C6(Carbamidomethyl) |
|  | O35490 | YIGGccGFEPYHIR | 1 | C5(Carbamidomethyl); C6(Carbamidomethyl) |
|  | O35490 | QVADEGDALVAGGVSQTPSYLScK | 2 | C23(Carbamidomethyl) |
|  | O35490 | LKAYLMSQPLAYHTPDcGK | 1 | C17(Carbamidomethyl) |
|  | O35490 | AYLMSQPLAYHTPDcGK | 1 | C15(Carbamidomethyl) |
|  | O35490 | ISGQKVNEAAcDIAR | 2 | C11(Carbamidomethyl) |
|  | O35490 | AGASIVGVNcHFDPSVSLQTVK | 1 | C10(Carbamidomethyl) |
| Ad-RIMKLA | O35490 | AGPWtPEAAVEHPEAVR | 1 | T5(Phospho) |
|  | O35490 | QQLEVFmKK | 1 | M7(Oxidation) |
|  | O35490 | QQLEVFmK | 1 | M7(Oxidation) |
|  | O35490 | LKAYLmSQPLAYHTPDcGK | 5 | M6(Oxidation); C17(Carbamidomethyl) |
|  | O35490 | GAAELmQQK | 2 | M6(Oxidation) |
|  | O35490 | GAAELmQQKEATTEQQLR | 4 | M6(Oxidation) |
|  | O35490 | AGSNVmQTFTFYASEDKLENR | 6 | M6(Oxidation) |
|  | O35490 | AYLmSQPLAYHTPDcGK | 3 | M4(Oxidation); C15(Carbamidomethyl) |
|  | O35490 | LmKEGLEAAR | 4 | M2(Oxidation) |
|  | O35490 | ASGKPVAATmcIGPEGDLHGVPPGEcAVR | 3 | M10(Oxidation); C11(Carbamidomethyl); C26(Carbamidomethyl) |
|  | O35490 | HGSWGSGLDmHTKPWIR | 3 | M10(Oxidation) |
|  | O35490 | IFRQQLEVFmKK | 1 | M10(Oxidation) |
|  | O35490 | YIGGccGFEPYHIR | 2 | C5(Carbamidomethyl); C6(Carbamidomethyl) |
|  | O35490 | QVADEGDALVAGGVSQTPSYLScK | 3 | C23(Carbamidomethyl) |
|  | O35490 | QVADEGDALVAGGVSQTPSYLScKSEVEVK | 1 | C23(Carbamidomethyl) |
|  | O35490 | LKAYLMSQPLAYHTPDcGK | 2 | C17(Carbamidomethyl) |
|  | O35490 | AYLMSQPLAYHTPDcGK | 2 | C15(Carbamidomethyl) |
|  | O35490 | ASGKPVAATMcIGPEGDLHGVPPGEcAVR | 3 | C11(Carbamidomethyl); C26(Carbamidomethyl) |
|  | O35490 | ISGQKVNEAAcDIAR | 6 | C11(Carbamidomethyl) |
|  | O35490 | AGASIVGVNcHFDPSVSLQTVK | 2 | C10(Carbamidomethyl) |

Table S3.

**Mass spectrometry data for BHMT1 modifications.**

Table S4.

**List of oligonucleotide primer pairs used in RT-PCR analysis.**

| Target Gene | Sense Primer (5'-3') | Antisense Primer (5'-3') | Annealing temperature |
| --- | --- | --- | --- |
| β-actin(M) | AGCCATGTACGTAGCCATCC | GCTGTGGTGGTGAAGCTGTA | 59℃ |
| RIMKLA(M) | AATGATCGATGAAGCTGAGCC | CCACACCACCTAGAAAGCAG | 59℃ |
| G6Pase(M) | AGGAAGGATGGAGGAAGGAA | TGGAACCAGATGGGAAAGAG | 59℃ |
| PEPCK(M) | ATCTTTGGTGGCCGTAGACCT | CCGAAGTTGTAGCCGAAGAA | 59℃ |
| SREBP1(M) | ACTTCTGGAGACATCGCAAAC | GGTAGACAACAGCCGCATC | 59℃ |
| FASn(M) | CTGCCACAACTCTGAGGACA | CGGATCACCTTCTTGAGAGC | 59℃ |
| ACC(M) | TGGTCGTGACTGCTCTGTGC | GTAGCC GAGGGTTCAGTTCC | 59℃ |
| SCD1(M) | TTCTTGCGATACACTCTGGTGC | CGGGATTGAATGTTCTTGTCGT | 59℃ |
| PPAR$\gamma$(M) | ACCACTCGCATTCCTTT | CACAGACTCGGCACTCA | 59℃ |
| ChREBP(M) | TTACTGGAAGCGGCGCATCG | CCAAGCAGCACAGGCACCAC | 59℃ |
| LXR(M) | TGCCATCAGCATCTTCTCTG | GGCTCACCAGCTTCATTAGC | 59℃ |
| Acox1(M) | CCGTCGAGAAATCGAGAACT | ATTGAGGCCAACAGGTTCCA | 59℃ |
| Cpt1$\alpha$(M) | ACGTTGGACGAATCGGAACA | GGTGGCCATGACATACTCCC | 59℃ |
| PPAR$\alpha$(M) | GTGGGTGGTTGAATCGTGAG | GCAGTGGAGTTTGGGTTGG | 59℃ |
| Scad(M) | ATGTGCCAGAGGAGCTGAGT | TGATCCACTGTTGCTTCTGC | 59℃ |
| Mcad(M) | AACTAAACATGGGCCAGCGA | CAGCTGCGACTGTAGGTCTG | 59℃ |
| Lcad(M) | GCATCAACATCGCAGAGAAA | ACGCTTGCTCTTCCCAAGTA | 59℃ |
| apoB(M) | TCACCATTTGCCCTCAACCTAA | GAAGGCTCTTTGGAAGTGTAAAC | 59℃ |
| Mtp(M) | ATCATCATTGGAGCCCTGGT | CATTCTTCAGGGCCAGCA | 59℃ |
| CD36(M) | TGGTCAAGCCAGCTAGAAA | CCCAGTCTCATTTAGCCAC | 59℃ |
| Fatp1(M) | CCGTATCCTCACGCATGTGT | CTCCATCGTGTCCTCATTGAC | 59℃ |
| Fatp2(M) | GATGCCGTGTCCGTCTTTTAC | GACTTCAGACCTCCACGACTC | 59℃ |
| Fatp5(M) | TCGGATCTGGGAATTCTACG | TTGGTTCTTTCGAACCTTGG | 59℃ |
| Fabp1(M) | GCCAGGAGAACTTTGAGC | TTGACGACTGCCTTGACT | 59℃ |
| AHR(M) | ACCAGAACTGTGAGGGTTGG | TCTGAGGTGCCTGAACTCCT | 59℃ |
| FFAR1(M) | CATCACTCTGCCCCTGAAG | AAGGCAAAGACTGGGCAGA | 59℃ |
| BHMT1(M) | GAGGCAGTTCGTCAGCTTCA | GCTTCGTTGACTTTCTGCCC | 59℃ |
| AP1(M) | GAGTCTCAGGAGCGGATCAA | CTGTTCCCTGAGCATGTTGG | 59℃ |
| β-actin(H) | GGTGGGCATGGGTCAGAAGG | GTACATGGCTGGGGTGTTGA | 59℃ |
| RIMKLA (H) | CCTCGGCCTCCAGCTAAAC | TCCAGCCAGTTCTTGGAACG | 59℃ |
| CD36(H) | TTGATGTGCAAAATCCACAGG | TGTGTTGTCCTCAGCGTCCT | 59℃ |
| BHMT1(H) | GCTTTTTGCCACCAGCTTCA | CTCGGCTGTTCCTTTGGTCA | 59℃ |

M: mouse; H: Human

Table S5.

**siRNA sequence against mouse BHMT1.**

| Dulex Name | SenseSeq | AntiSeq |
| --- | --- | --- |
| BHMT1 (M) | 5′-GUGAAGACAAGCUGGAAAAd (TT)-3′ | 3′-d(TT)CACUUCUGUUCGACCUUUU-5′ |

Table S6.

**Characteristics of patients with non-NAFLD and NAFLD.**

|  | non-NAFLD liver (Paracancerous) | NAFLD liver | P value |
| --- | --- | --- | --- |
| Sex (Male/Female) | 3/2 | 2/3 |  |
| Age | 50.40±2.65 | 51.00±4.15 | P=0.8136 |
| ALT(U/L) | 26.74±9.90 | 29.20±11.62 | P=0.7555 |
| AST(U/L) | 33.48±14.36 | 20.00±5.76 | P=0.1197 |
| Fasting Glucose (mM) | 4.98±0.62 | 6.71±0.92 | P=0.0142* |
| TG (mM) | 0.65±0.15 | 2.03±0.96 | P=0.0215* |
| TC (mM) | 2.74±0.42 | 4.53±1.20 | P=0.0223* |
| HDL (mM) | 0.81±0.17 | 0.99±0.25 | P=0.2664 |
| LDL (mM) | 1.62±0.31 | 2.67±1.04 | P=0.0898 |

Data were presented as mean±SD. *P<0.05 was considered as statistically significant.

Table S7.

**Characteristics of healthy subjects and patients with diabetes.**

|  | Healthy | Diabetic | P value |
| --- | --- | --- | --- |
| Sex (Male/Female) | 8/12 | 8/12 |  |
| Age | 48.25±5.97 | 52.40±12.07 | P=0.1871 |
| Fasting Glucose(mM) | 5.10±0.45 | 15.77±4.85 | P<0.0001* |
| HbA1c(%) | 5.57±0.27 | 10.36±1.92 | P<0.0001* |
| BMI (kg/m^2^) | 21.90±1.51 | 26.30±3.16 | P<0.0001* |
| TG (mM) | 1.38±0.83 | 1.95±1.01 | P=0.0686 |
| TC (mM) | 4.97±0.90 | 4.89±1.27 | P=0.8216 |
| HDL (mM) | 1.42±0.37 | 1.15±0.83 | P=0.0355* |
| LDL (mM) | 2.99±0.68 | 3.18±0.91 | P=0.4694 |

Data were presented as mean±SD. *P<0.05 was considered as statistically significant.
