# Supplementary material for: Correction: Ribosomal modification protein rimK-like family member A activates betaine-homocysteine S-methyltransferase 1 to ameliorate hepatic steatosis
Source: Signal Transduct Target Ther. 2024 Dec 11;9:360. doi: 10.1038/s41392-024-02054-1 (PMC11634956; doi:10.1038/s41392-024-02054-1)

Original WB images

Ribosomal modification protein rimK-like family member A activates betaine-homocysteine S-methyltransferase 1 to ameliorate hepatic steatosis

Han Yan, Wenjun Liu, Rui Xiang, Xin Li, Song Hou, Luzheng Xu, Lin Wang, Dong Zhao, Xingkai Liu, Guoqing Wang, Yujing Chi, Jichun Yang

The bands for the representative images used in the manuscript had been marked in red box.

Figure 1c: RIMKLA

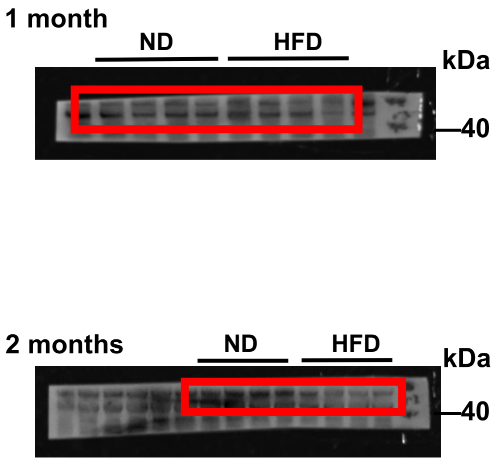

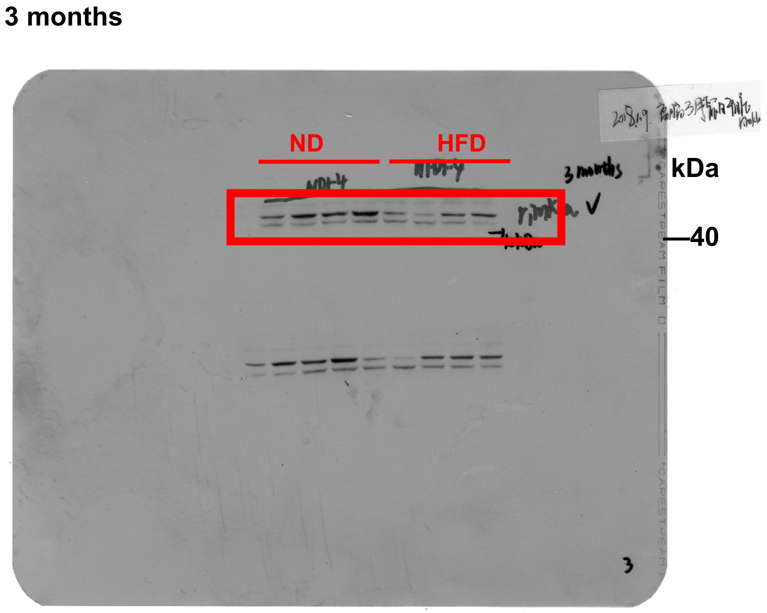

Figure 1c: β-actin

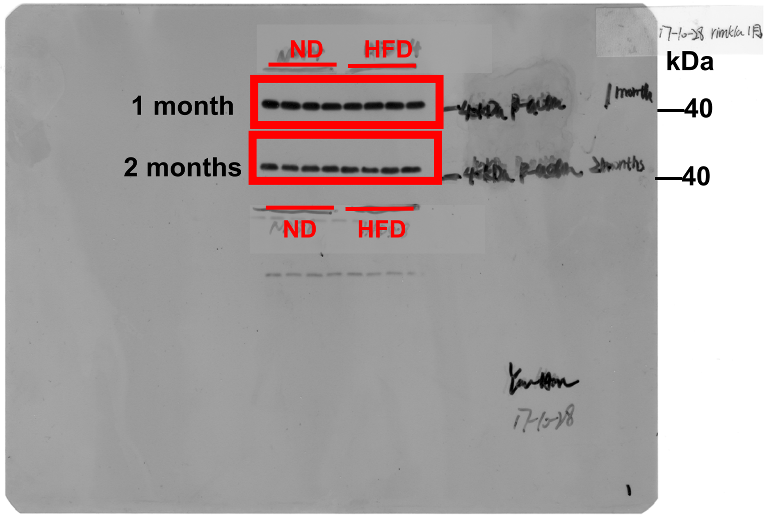

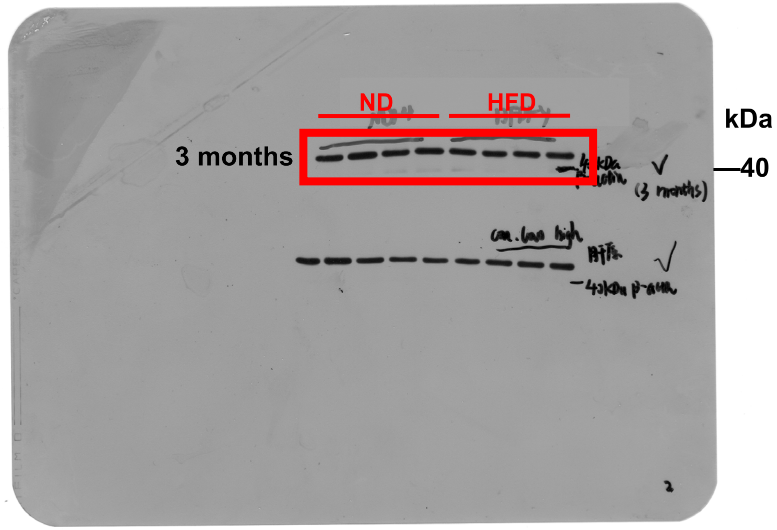

Figure 1f: RIMKLA

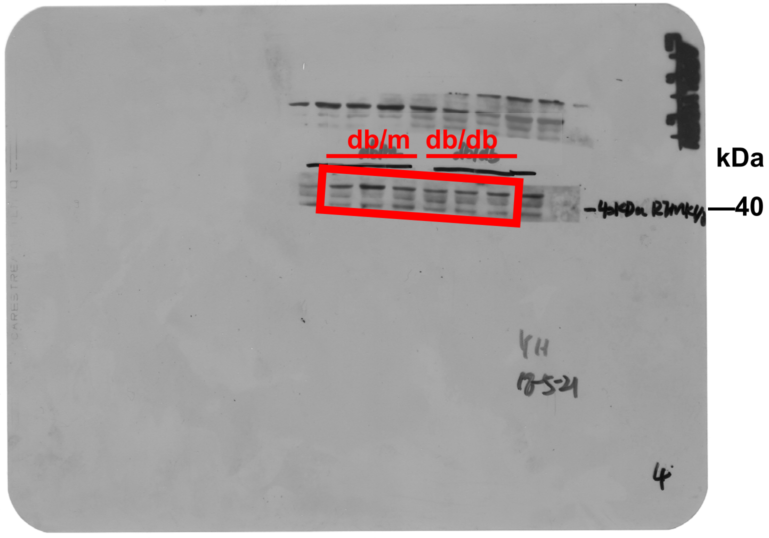

Figure 1f: β-actin

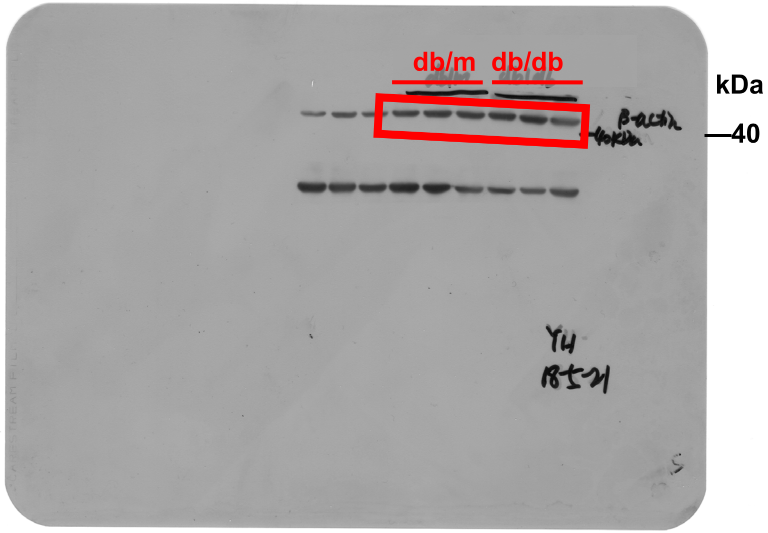

Figure 2b: RIMKLA

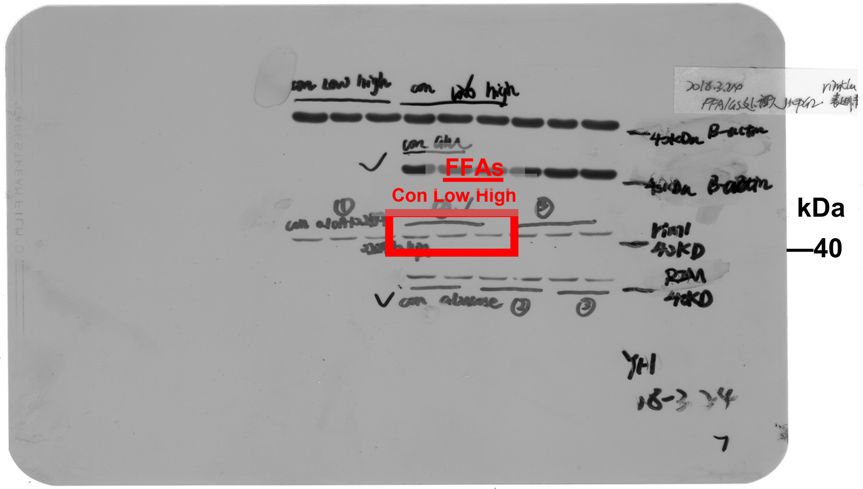

Figure 2d: RIMKLA

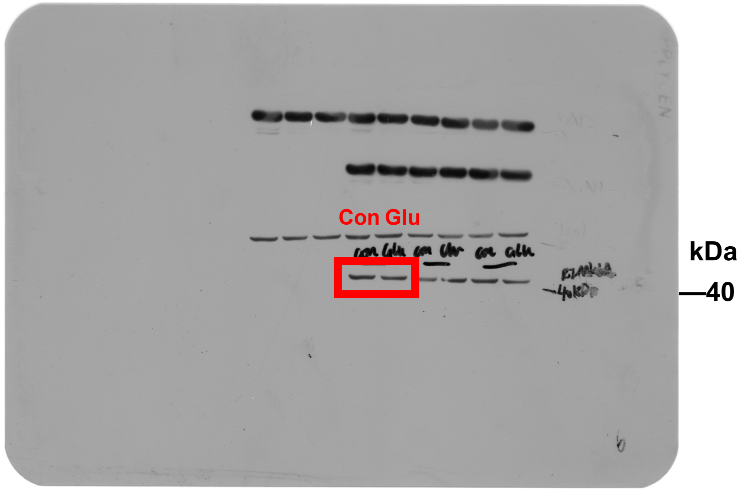

Figure 2f: RIMKLA

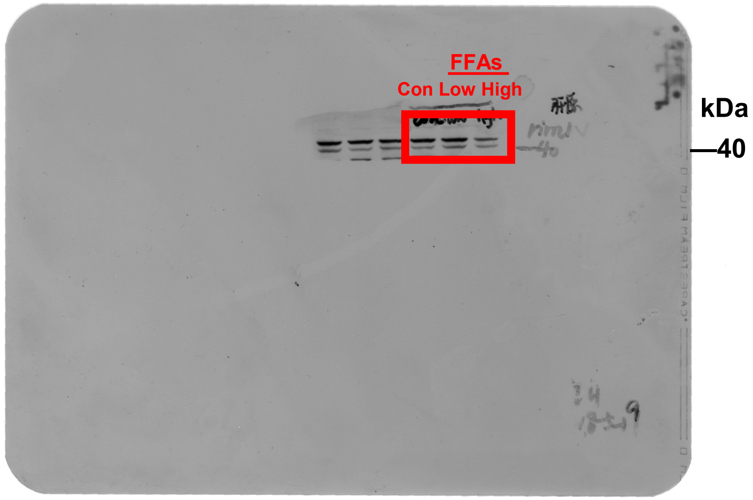

Figure 2b: β-actin

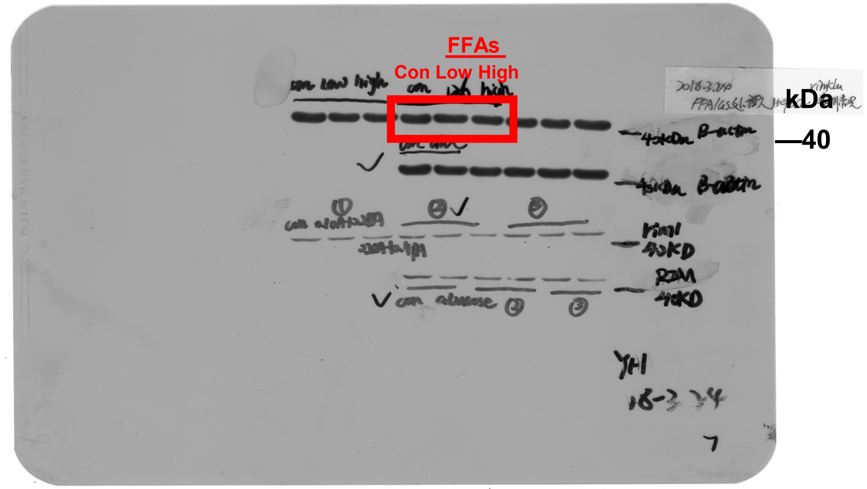

Figure 2d: β-actin

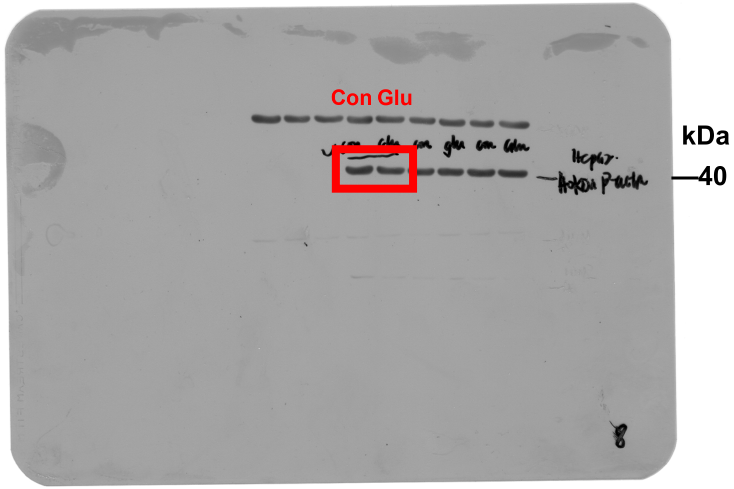

Figure 2f: β-actin

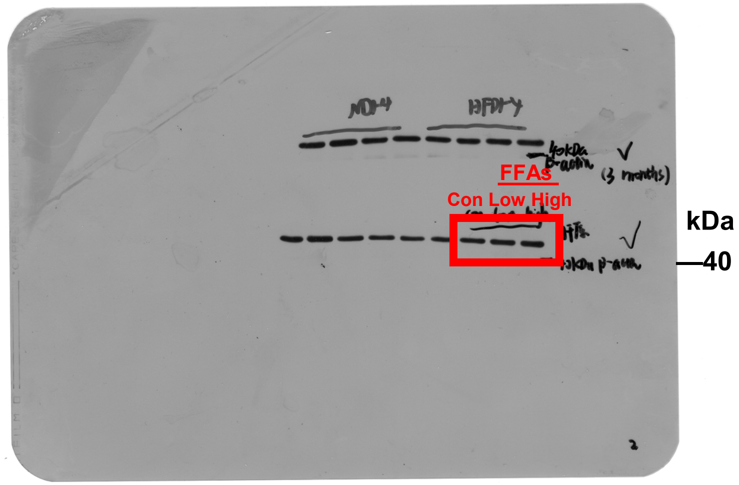

Figure 2h: RIMKLA

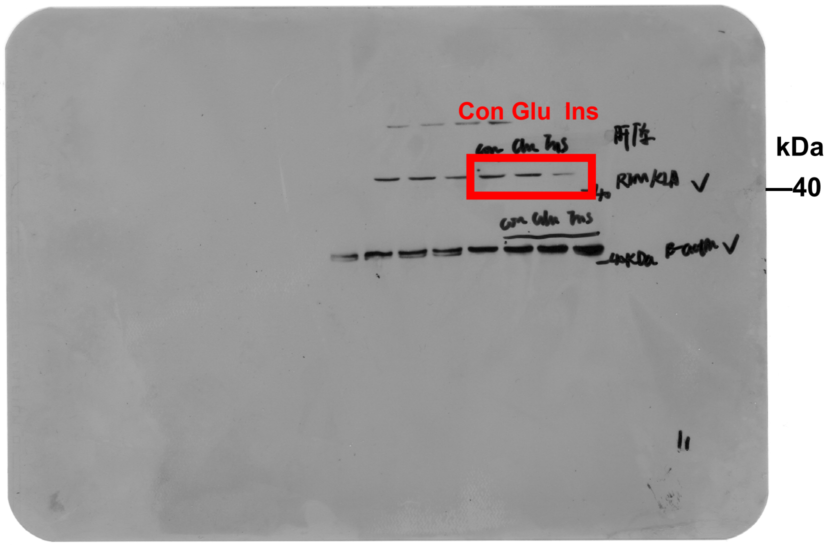

Figure 2h: β-actin

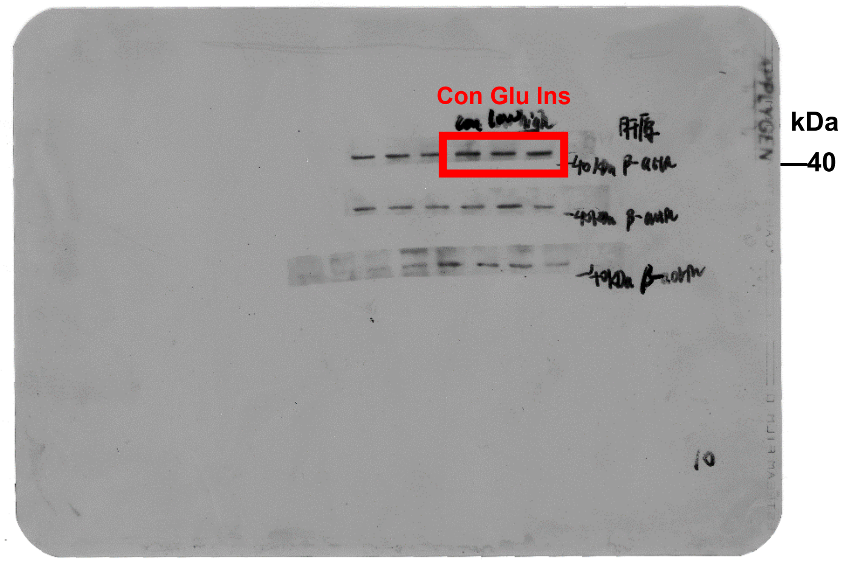

Figure 4a: RIMKLA

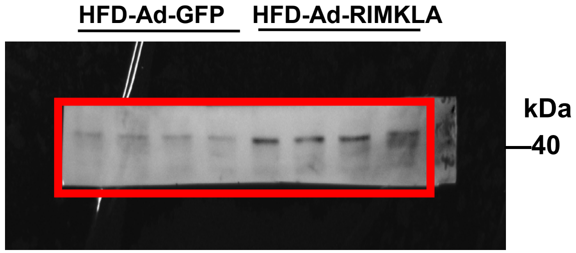

Figure 4a: PEPCK

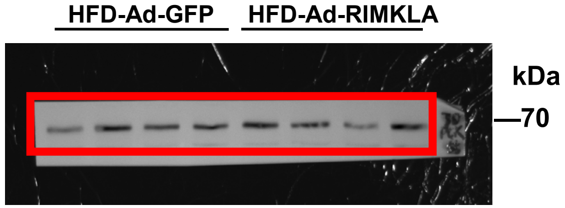

Figure 4a: pFOXO1

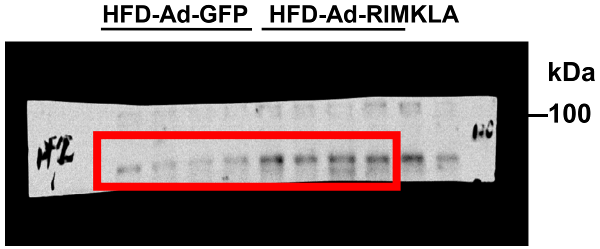

Figure 4a: pAkt

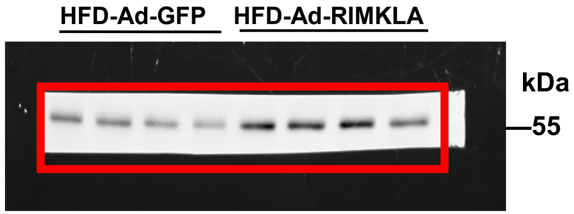

Figure 4a: G6Pase

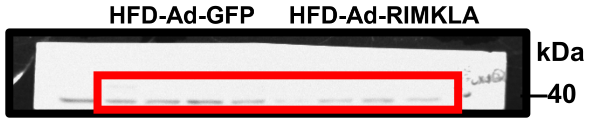

Figure 4a: FOXO1

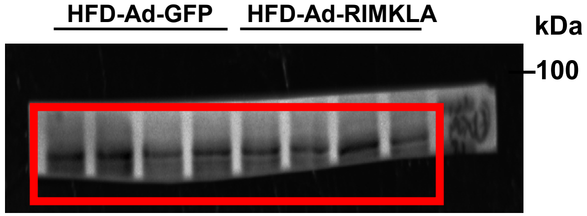

Figure 4a: Akt

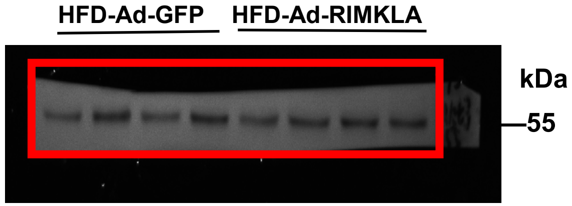

Figure 4a: β-actin

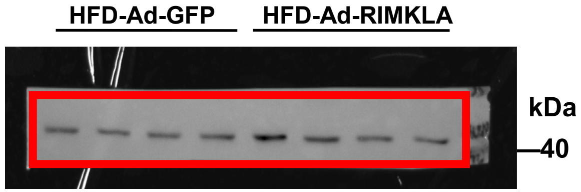

Figure 4c: RIMKLA

Mouse hepatocytes

Ad-GFP Ad-RIMKLA

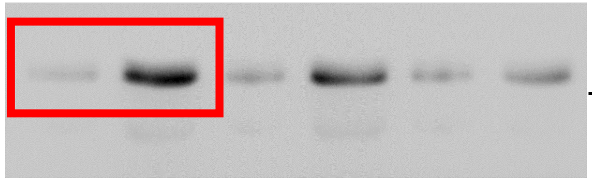

kDa

40

Figure 4c: pFOXO1

Mouse hepatocytes

Ad-GFP Ad-RIMKLA

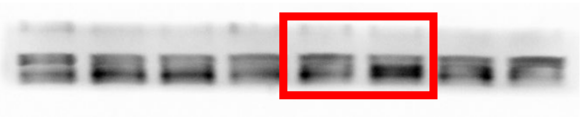

kDa

100

Figure 4g: FASn

HFD-Ad-GFP HFD-Ad-RIMKLA

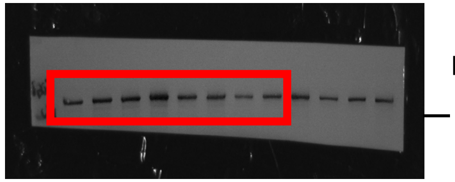

kDa

170

Figure 4c: PEPCK

Mouse hepatocytes

Ad-GFP Ad-RIMKLA

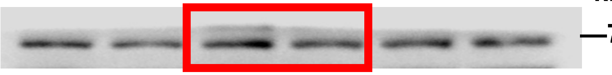

kDa

70

Figure 4c: FOXO1

Mouse hepatocytes

Ad-GFP Ad-RIMKLA

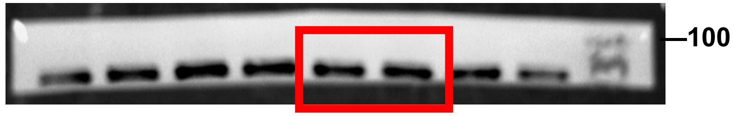

kDa

100

Figure 4g: CD36

HFD-Ad-GFP HFD-Ad-RIMKLA

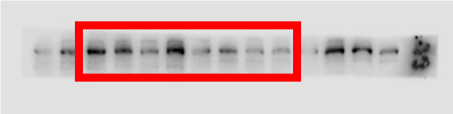

kDa

130

Figure 4c: G6Pase

Mouse hepatocytes

Ad-GFP Ad-RIMKLA

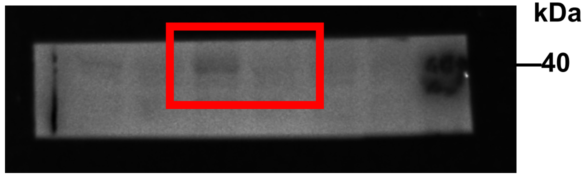

kDa

40

Figure 4c: β-actin

Mouse hepatocytes

Ad-GFP Ad-RIMKLA

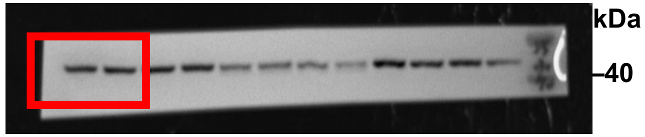

kDa

40

Figure 4g: β-actin

HFD-Ad-GFP HFD-Ad-RIMKLA

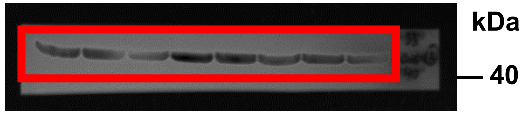

kDa

40

Figure 4h: FASn

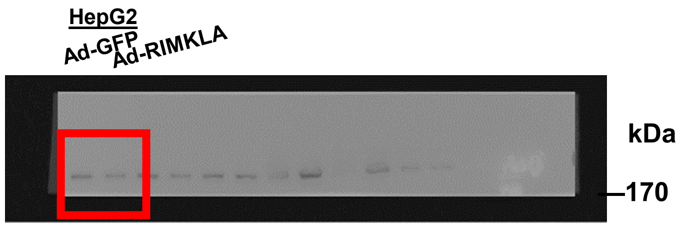

Figure 4i: FASn

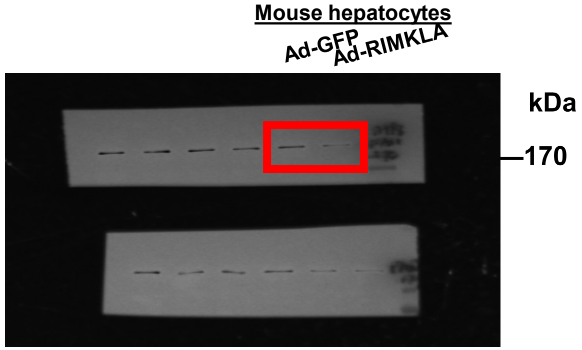

Figure 4j: FASn

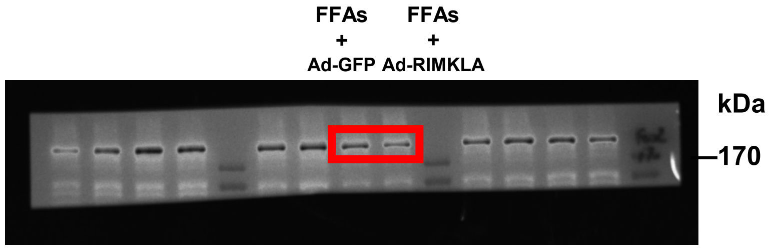

Figure 4h: CD36

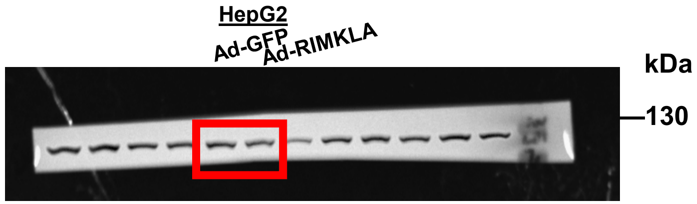

Figure 4i: CD36

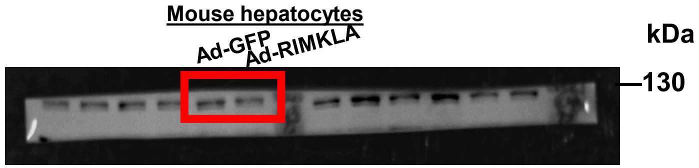

Figure 4j: CD36

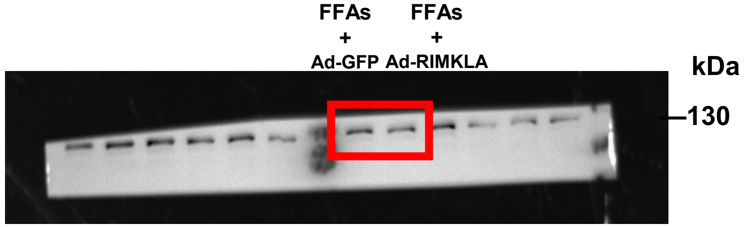

Figure 4h: β-actin

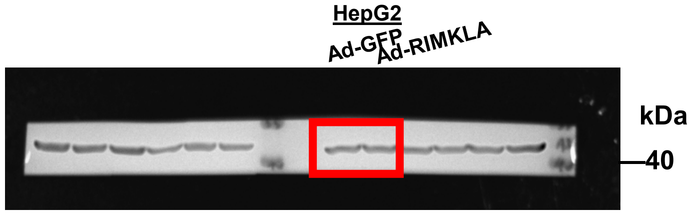

Figure 4i: β-actin

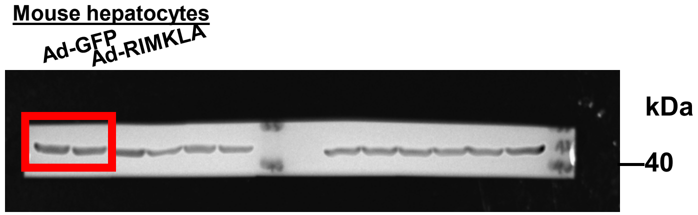

Figure 4j: β-actin

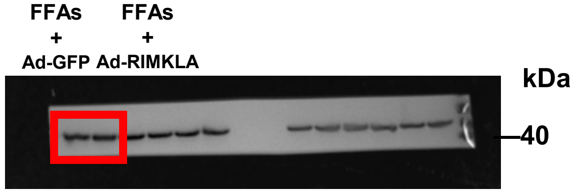

Figure 5a

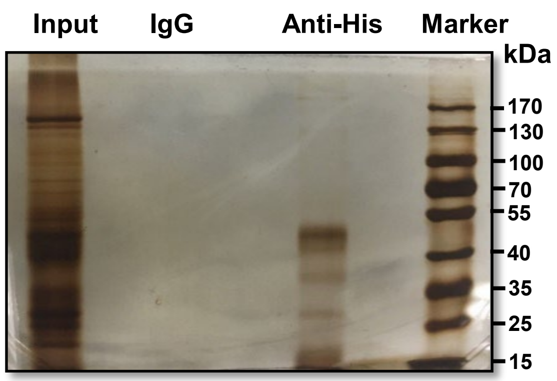

Figure 5b: RIMKLA(upper)

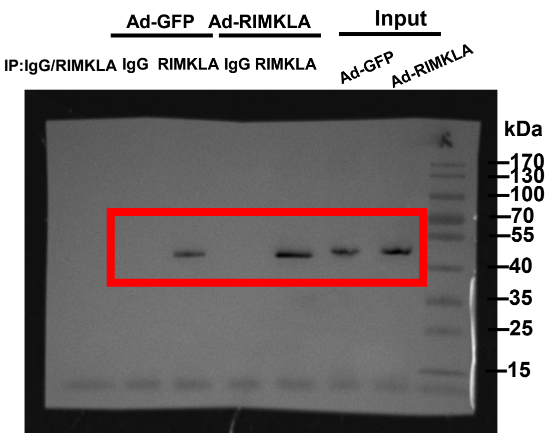

Figure 5b: BHMT1(upper)

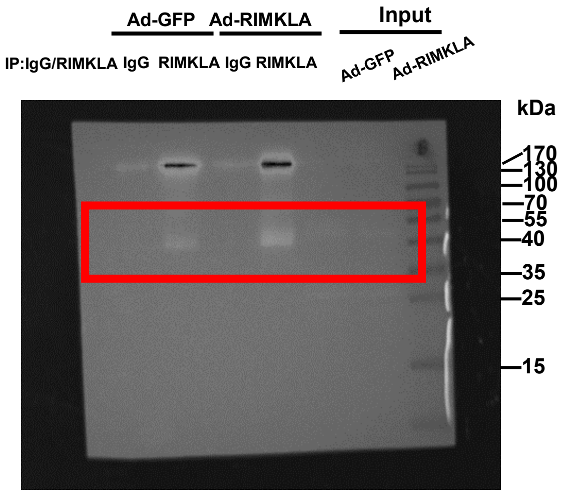

Figure 5b: BHMT1(lower)

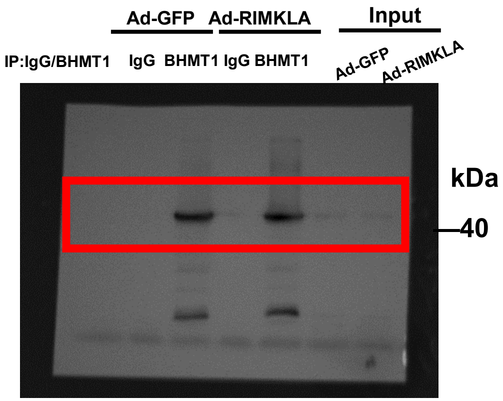

Figure 5b: RIMKLA(lower)

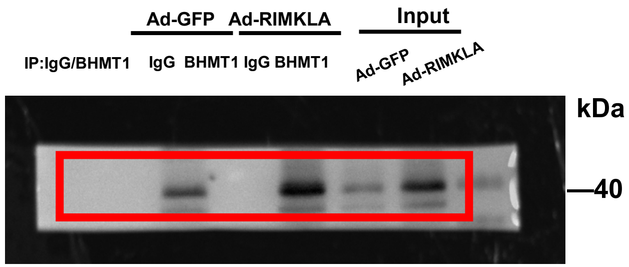

Figure 5c: RIMKLA(left)

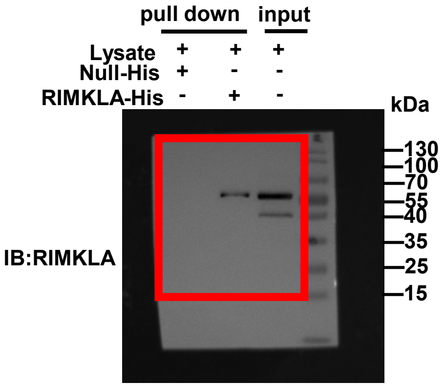

Figure 5f: BHMT1

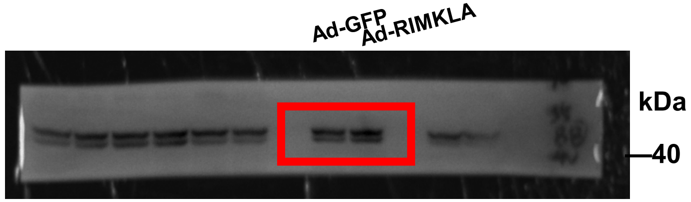

Figure 5h: pSer

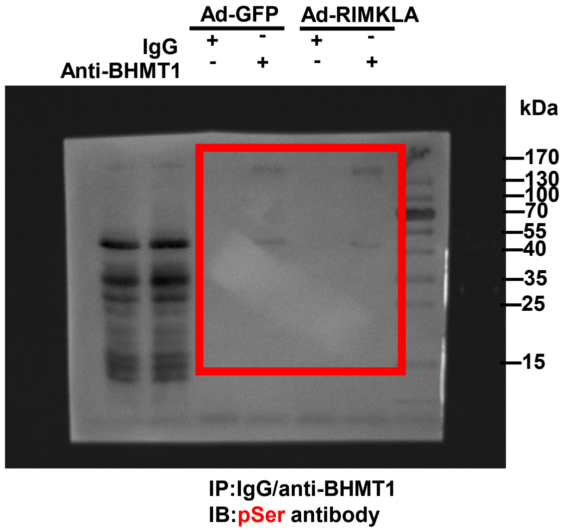

Figure 5c: BHMT1(right)

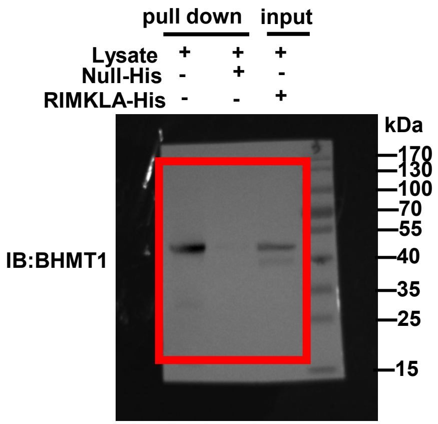

Figure 5f:β-actin

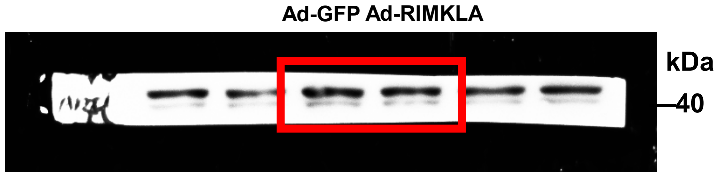

Figure 5h: pThr

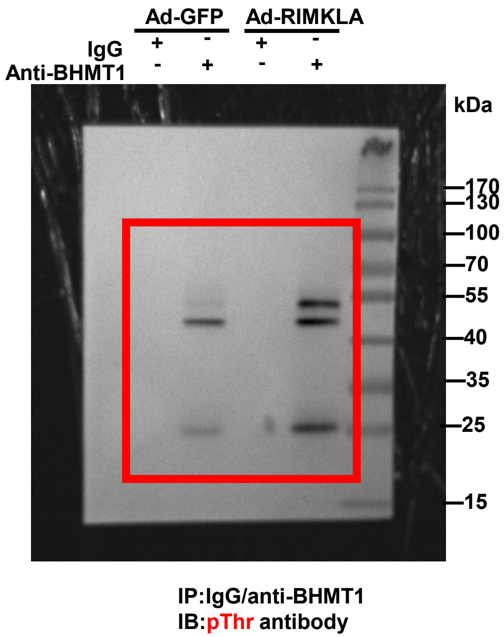

Figure 5I: RIMKLA

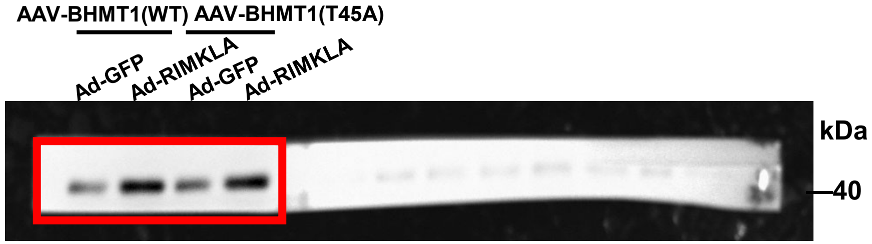

Figure 5I: FASn

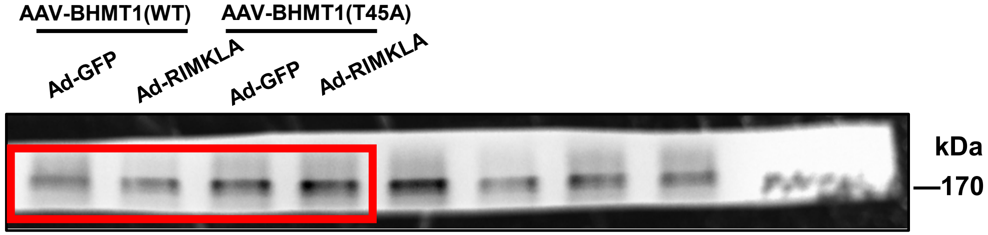

Figure 5I: pBHMT1^{T45}

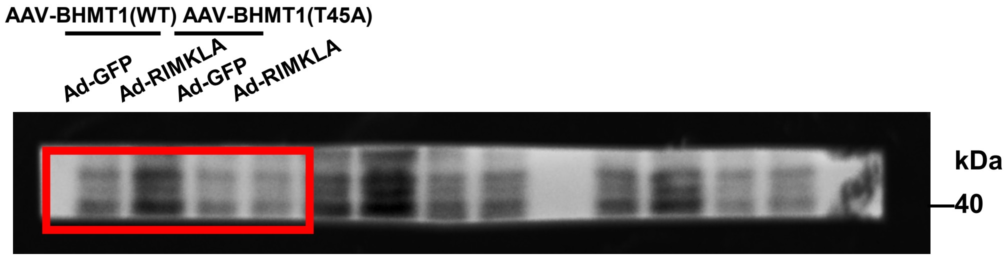

Figure 5I: CD36

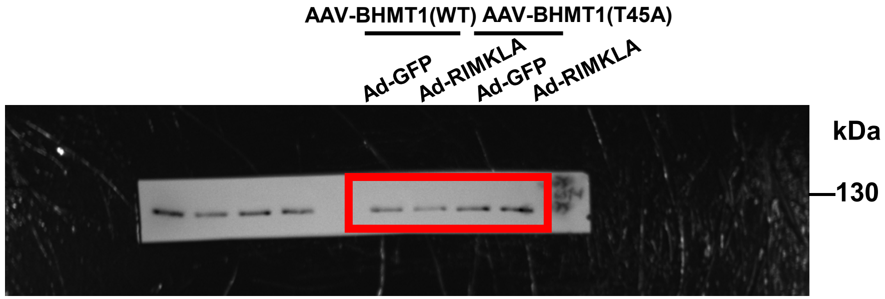

Figure 5I: BHMT1

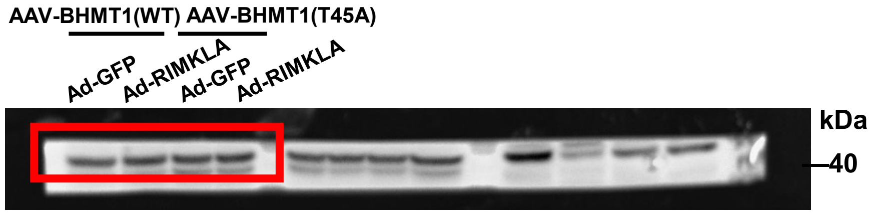

Figure 5I: β-actin

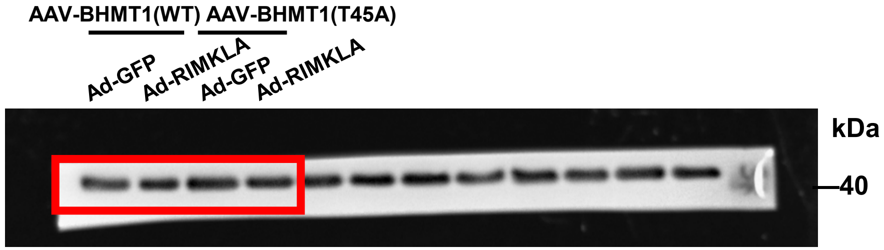

Figure 5n: BHMT1

RIMKLA^fl/fl RIMKLA^hep-/-
FFAs - + - +

kDa

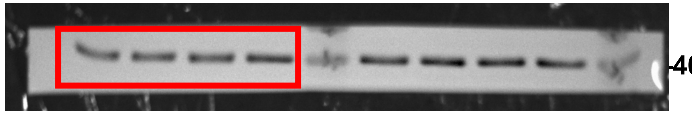

40

Figure 5n: pBHMT1^T45

RIMKLA^fl/fl RIMKLA^hep-/-
FFAs - + - +

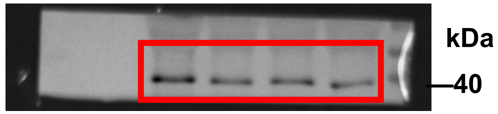

kDa
40

Figure 5n: RIMKLA

RIMKLA^fl/fl RIMKLA^hep-/-
FFAs - + - +

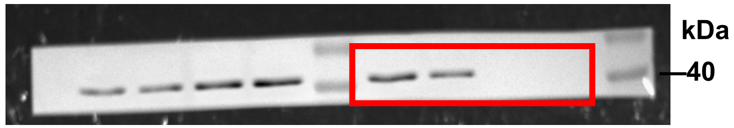

kDa
40

Figure 5n: β-actin

RIMKLA^fl/fl RIMKLA^hep-/-
FFAs - + - +

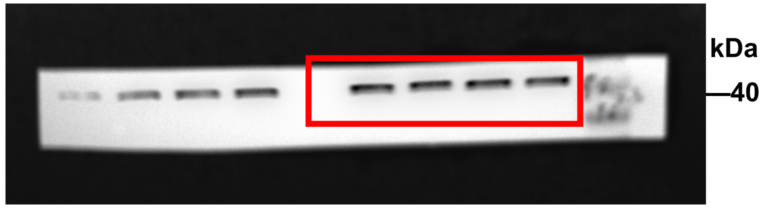

kDa
40

Figure 5o: pBHMT1^T45

RIMKLA^hep-/- hepatocyte
rRIMKLA - +

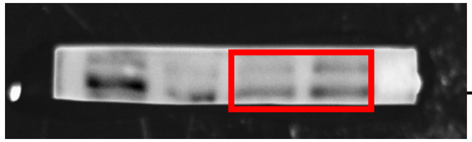

kDa
40

Figure 5o: BHMT1

RIMKLA^hep-/- hepatocyte
rRIMKLA - +

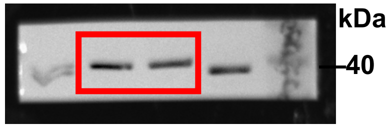

kDa
40

Figure 5o: RIMKLA

RIMKLA^hep-/- hepatocyte
rRIMKLA - +

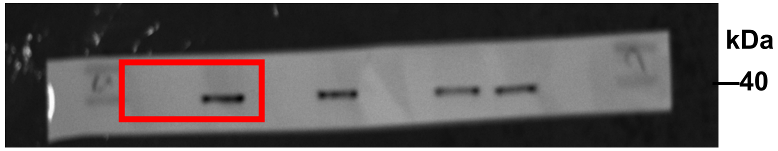

kDa
40

Figure 5o: β-actin

RIMKLA^hep-/- hepatocyte
rRIMKLA - +

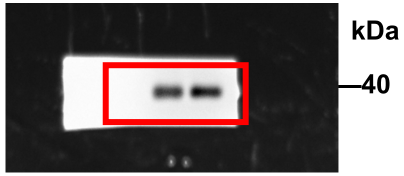

kDa
40

Figure 5p:BHMT1

BHMT1 + + +
rRIMKLA - + -
Null-RIMKLA - - +

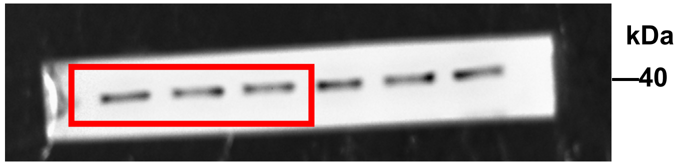

kDa
40

Figure 5p: pBHMT1^T45

BHMT1 + + +
rRIMKLA - + -
Null-RIMKLA - - +

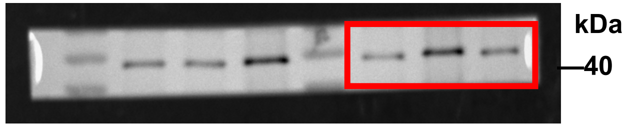

kDa
40

Figure 5p: RIMKLA

BHMT1 + + +
rRIMKLA - + -
Null-RIMKLA - - +

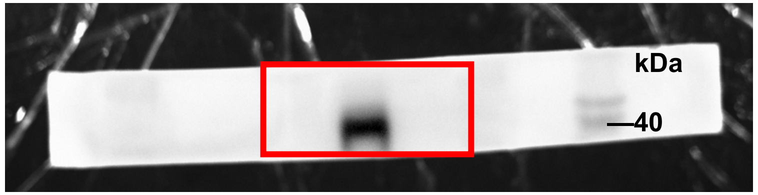

Figure 6d: pBHMT1^{T45}

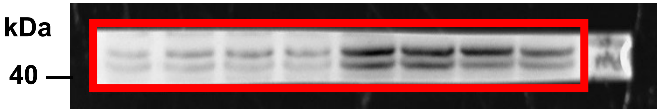

Figure 6d: BHMT1

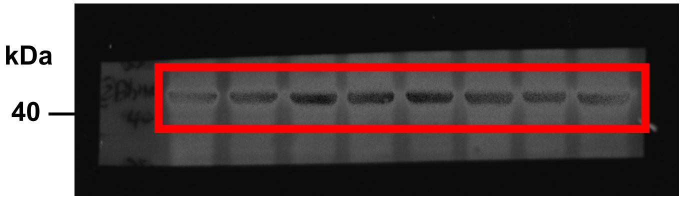

Figure6d: β-actin

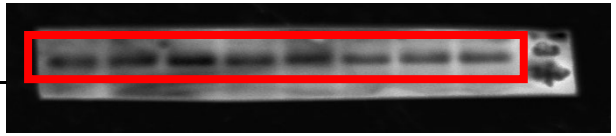

Figure 6i: FASn

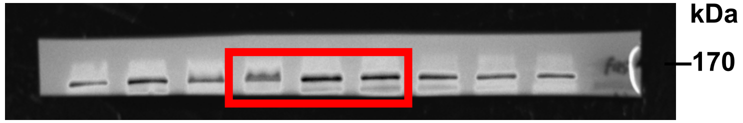

Figure 6i: CD36

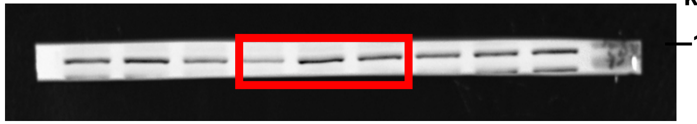

Figure6i: β-actin

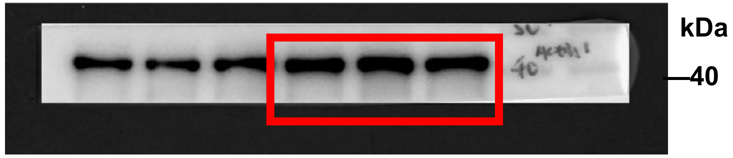

Figure 6j: FASn

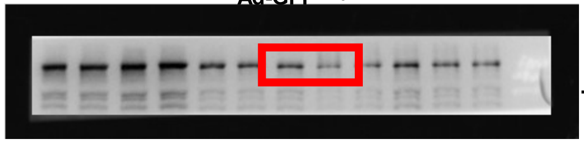

Figure 6j: CD36

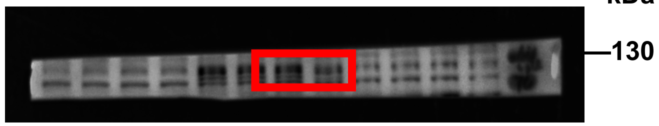

Figure6j: β-actin

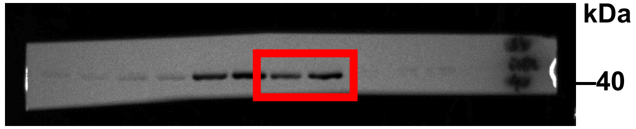

Figure 7a: pAP1

pcDNA3.1 AP1 Plasmid

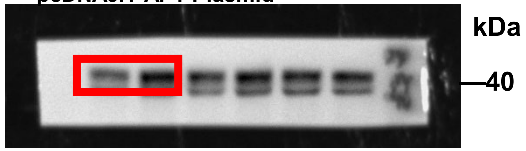

kDa
—40

Figure 7a: AP1

pcDNA3.1 AP1 Plasmid

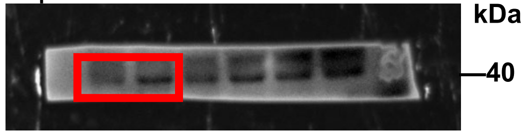

kDa
—40

Figure 7a: FASn

pcDNA3.1 AP1 Plasmid

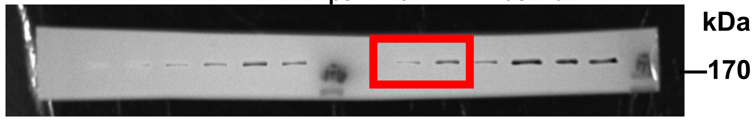

kDa
—170

Figure 7a: CD36

pcDNA3.1 AP1 Plasmid

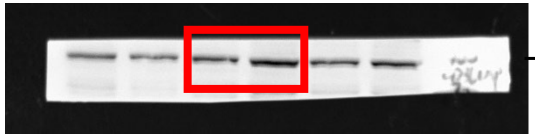

kDa
—130

Figure 7a: β-actin

pcDNA3.1 AP1 Plasmid

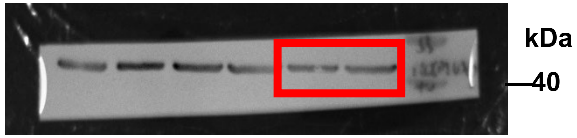

kDa
—40

Figure 7b: pAP1

Con 100μM 500μM (Hcy)

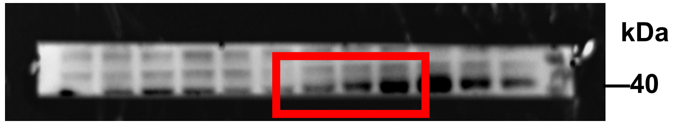

kDa
—40

Figure 7b: AP1

Con 100μM 500μM (Hcy)

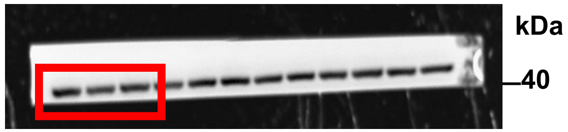

kDa
—40

Figure 7b: β-actin

Con 100μM 500μM (Hcy)

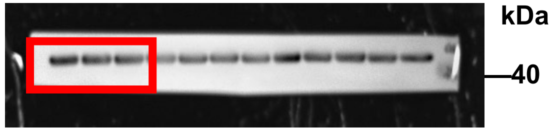

kDa
—40

Figure 7c: FASn

Con Hcy Hcy+SR11302

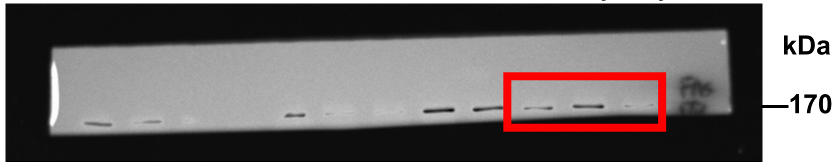

kDa
—170

Figure 7c: CD36

Con Hcy Hcy+SR11302

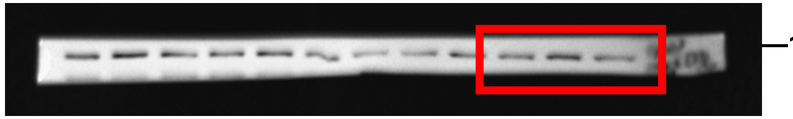

kDa
—130

Figure 7c: β-actin

Con Hcy Hcy+SR11302

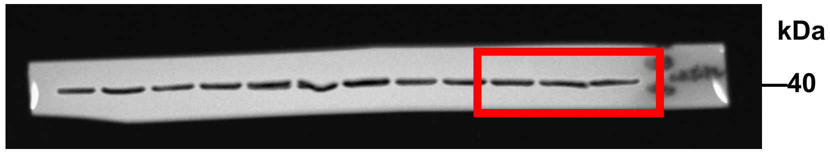

kDa
—40

Figure 7d: pAP1

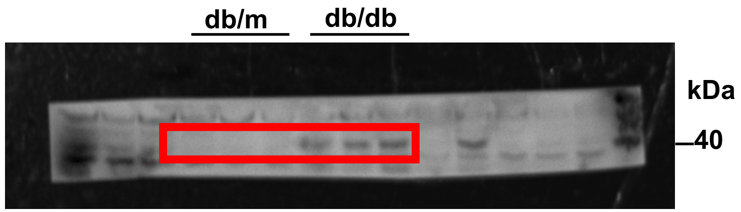

Figure 7e: pAP1

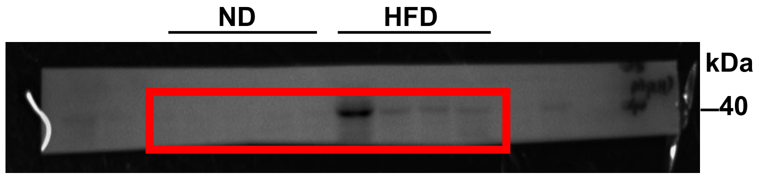

Figure 7f: pAP1

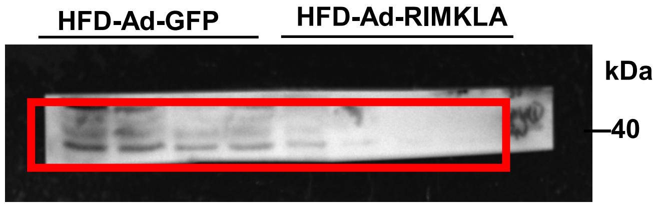

Figure 7d: AP1

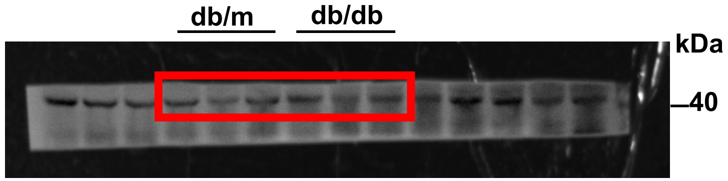

Figure 7e: AP1

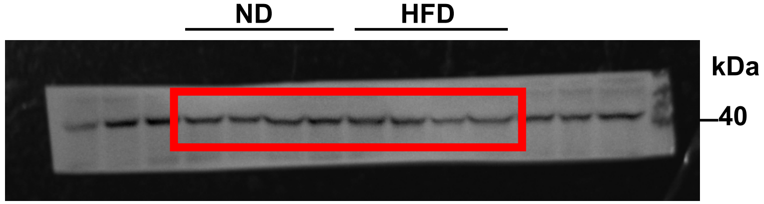

Figure 7f: AP1

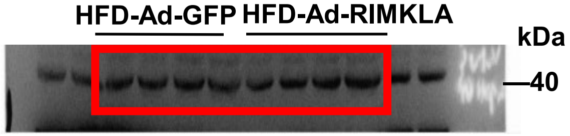

Figure 7d: β-actin

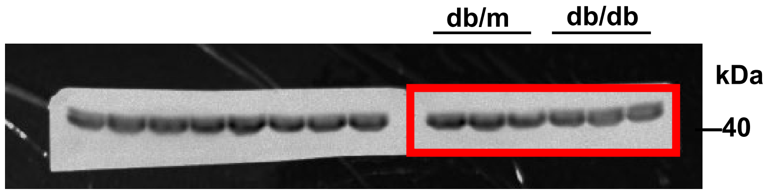

Figure 7e: β-actin

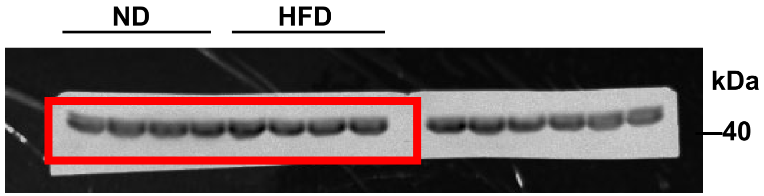

Figure 7f: β-actin

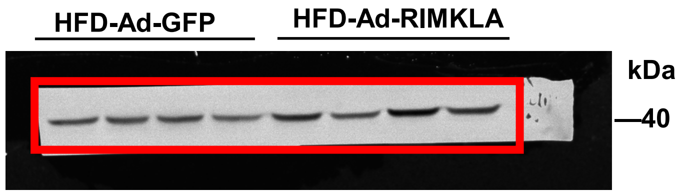

Figure 7g: pAP1

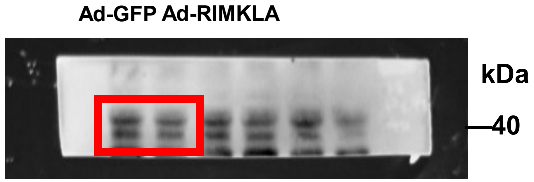

Figure 7g: AP1

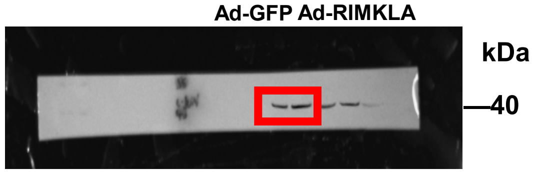

Figure 7g: β-actin

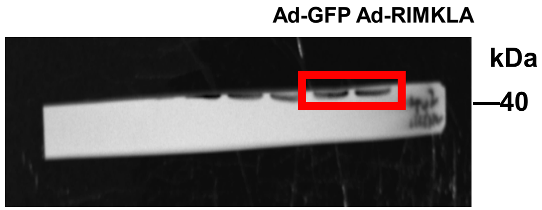

Figure 7j: pAP1

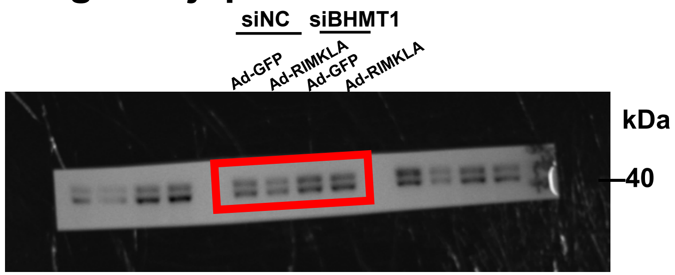

Figure 7j: AP1

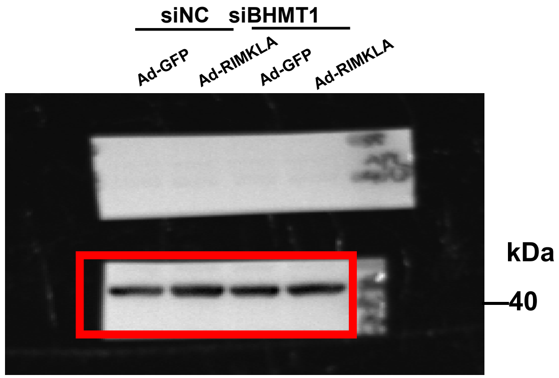

Figure 7j: FASn

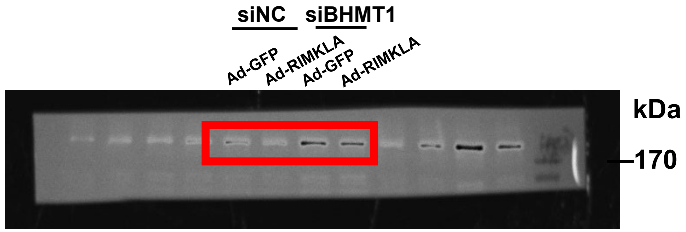

Figure 7j: CD36

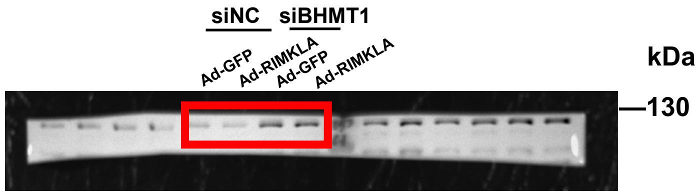

Figure 7j: β-actin

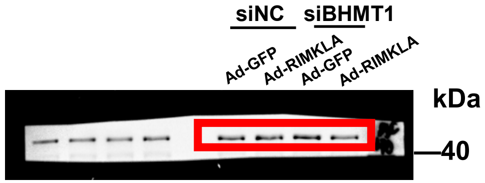

Figure 8i: RIMKLA

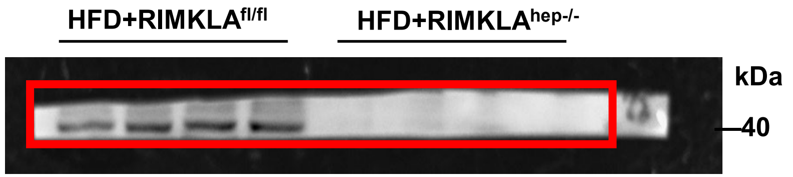

Figure 8i: pAP1

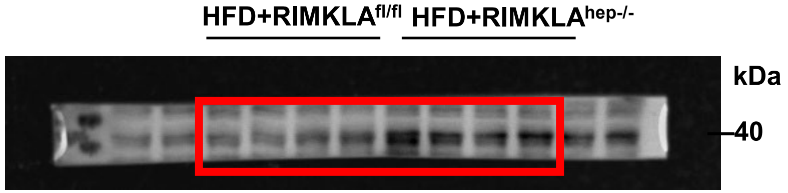

Figure 8i: FASn

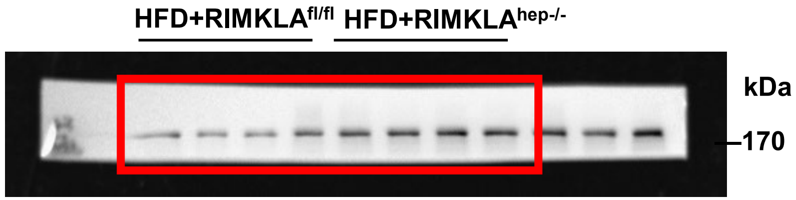

Figure 8i: pBHMT1^{T45}

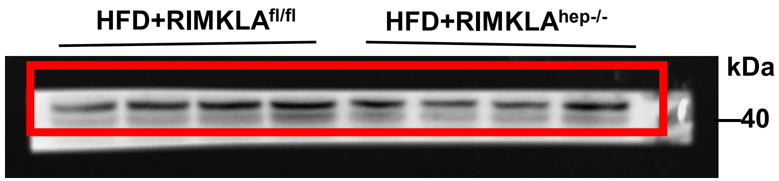

Figure 8i: AP1

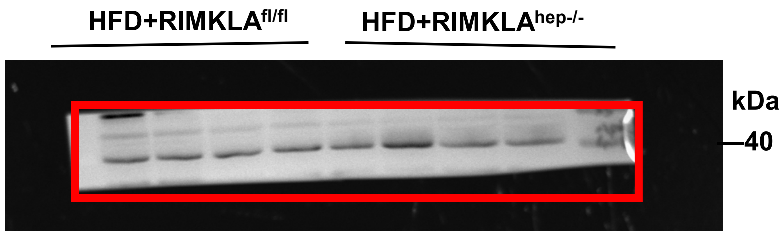

Figure 8i: CD36

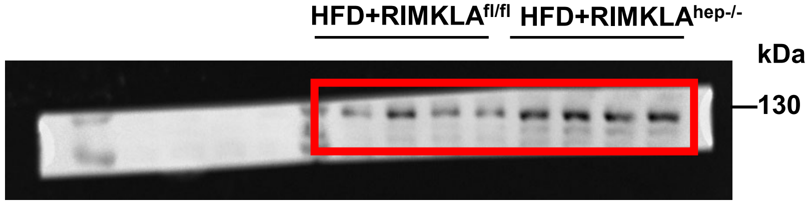

Figure 8i: BHMT1

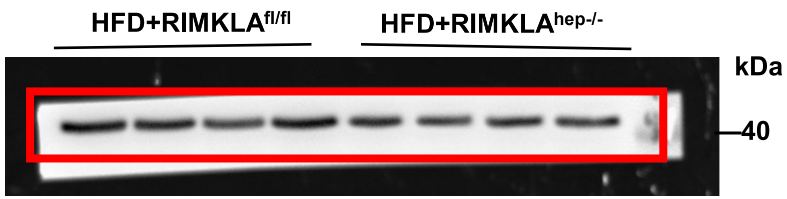

Figure 8i: β-actin

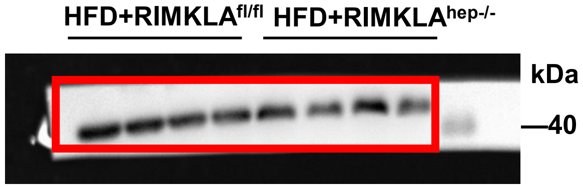

Figure 8I: RIMKLA

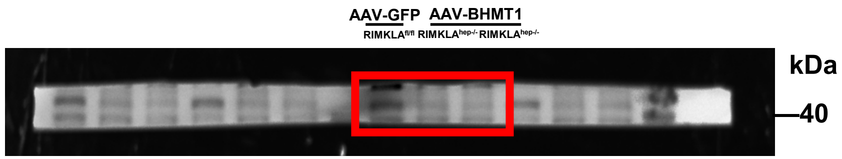

Figure 8I: pAP1

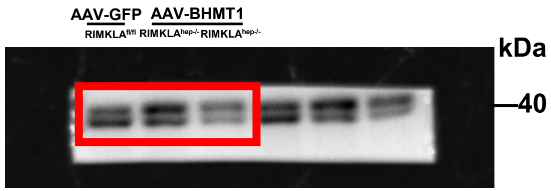

Figure 8I: FASn

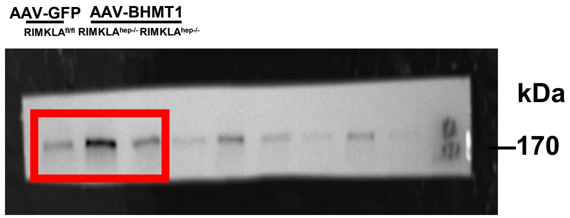

Figure 8I: pBHMT1^{T45}

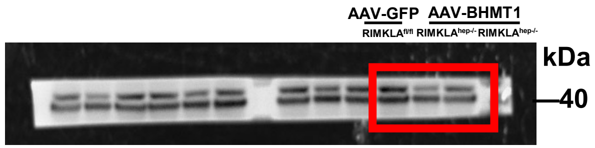

Figure 8I: AP1

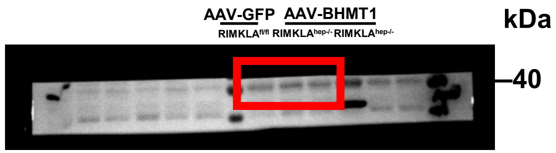

Figure 8I: CD36

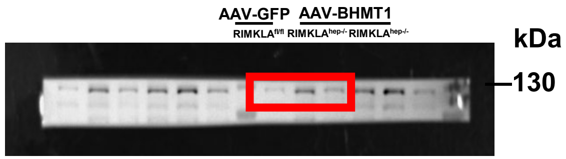

Figure 8I: BHMT1

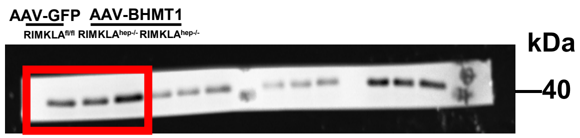

Figure 8I:β-actin

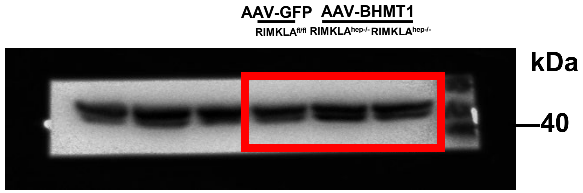

Suppl figure 1e: RIMKLA

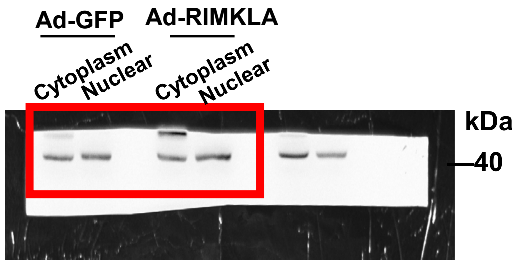

Suppl figure 1e: LaminB1

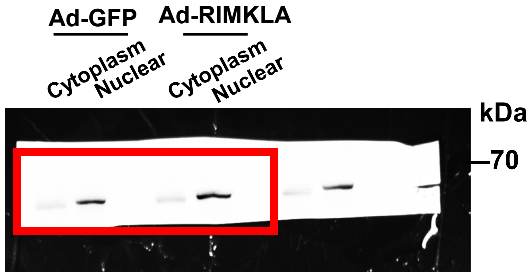

Suppl figure 1e: β-actin

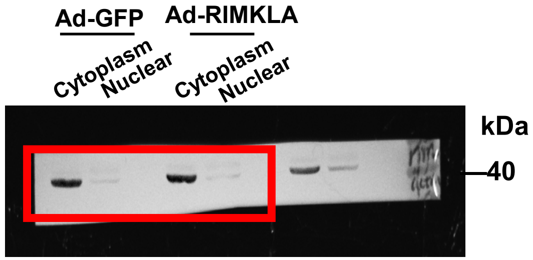

Suppl figure 1f: RIMKLA

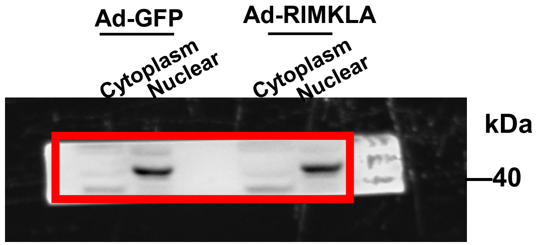

Suppl figure 1f: LaminB1

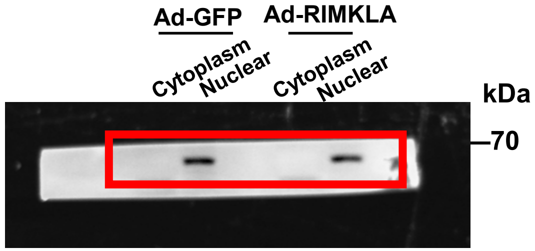

Suppl figure 1f: β-actin

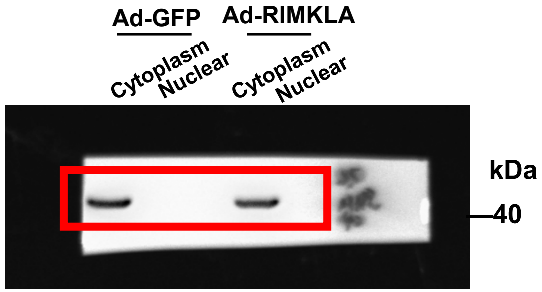

Suppl figure 1g: RIMKLA

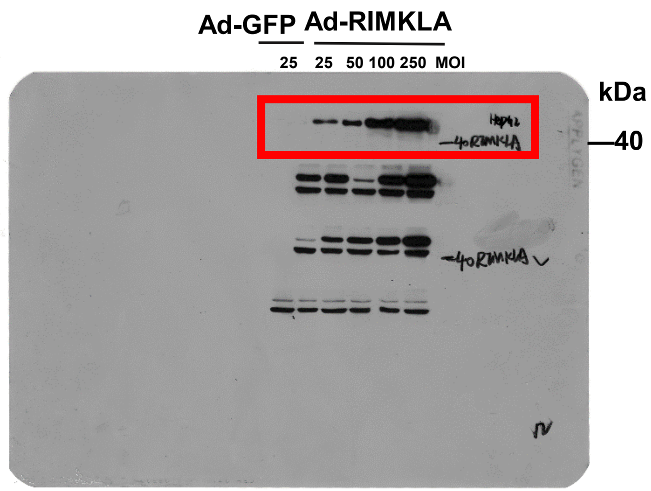

Suppl figure 1h: RIMKLA

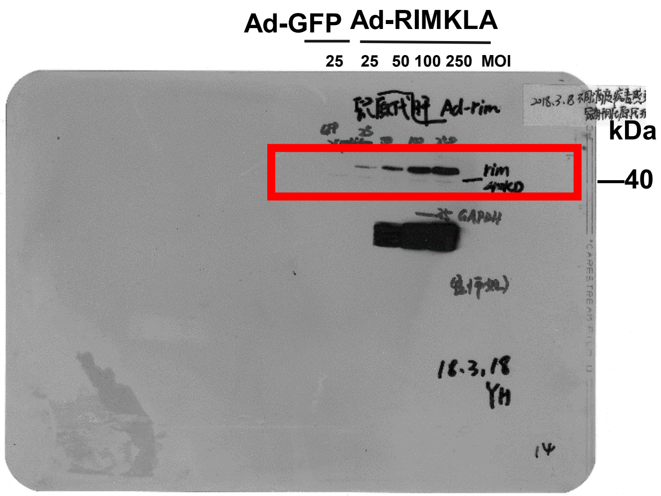

Suppl figure 1i: RIMKLA

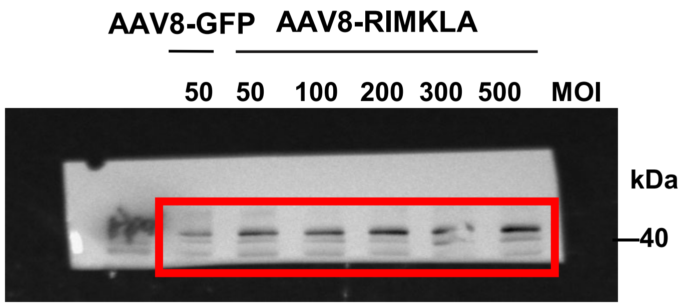

Suppl figure 1g: β-actin

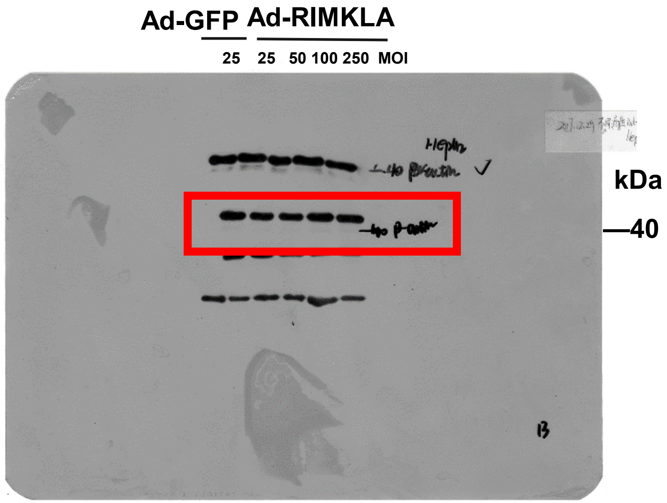

Suppl figure 1h: β-actin

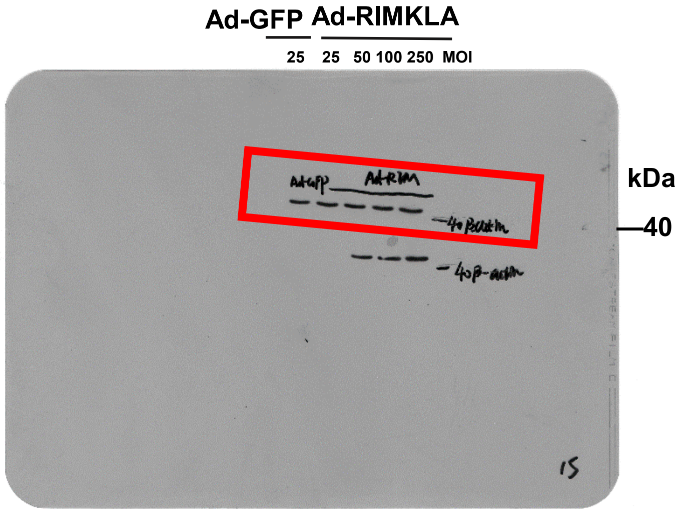

Suppl figure 1i: β-actin

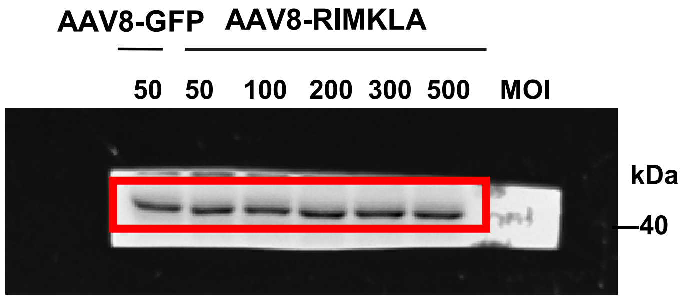

Suppl figure 1j: RIMKLA

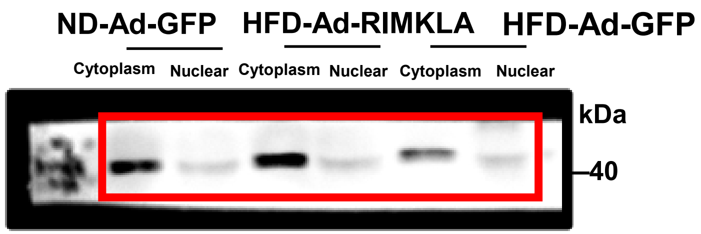

Suppl figure 1j: LaminB1

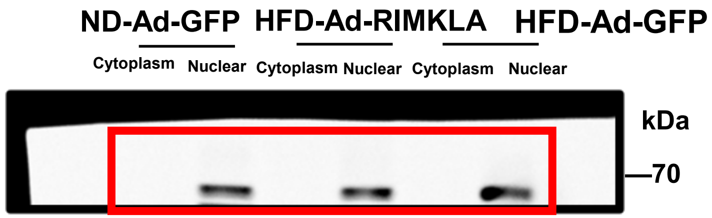

Suppl figure 1j: β-actin

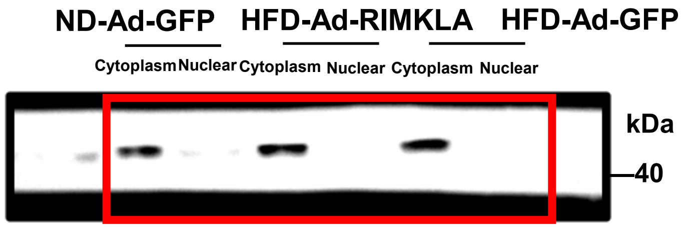

Suppl figure 4a: RIMKLA

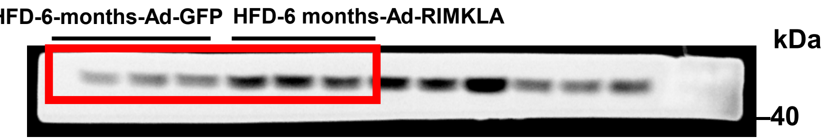

Suppl figure 4a: pAkt

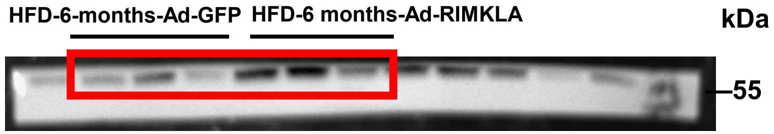

Suppl figure 4a: Akt

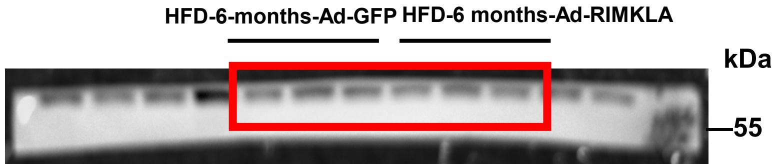

Suppl figure 4a: PEPCK

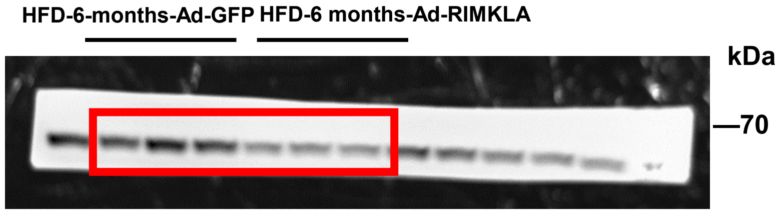

Suppl figure 4a: G6Pase

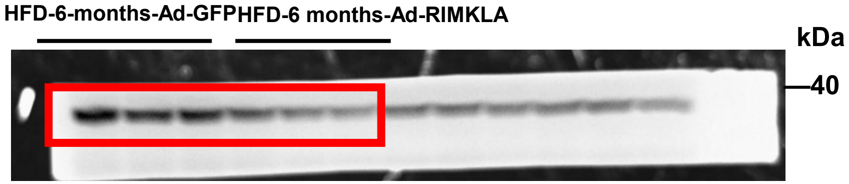

Suppl figure 4a: pFOXO1

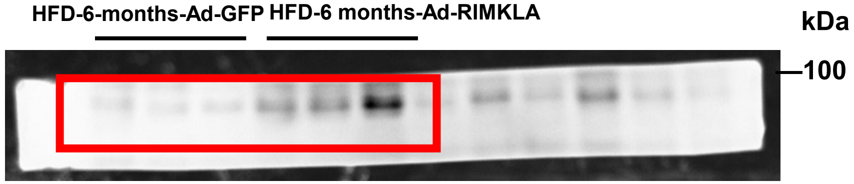

Suppl figure 4a: FOXO1

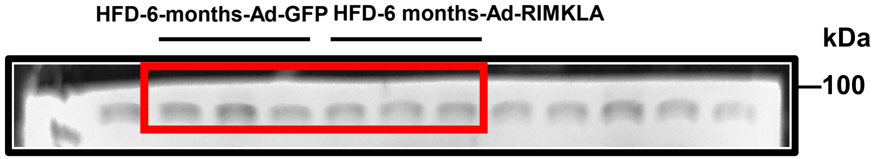

Suppl figure 4a: FASn

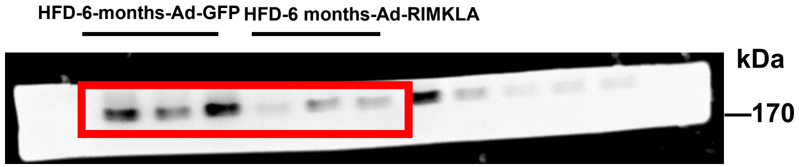

Suppl figure 4a: CD36

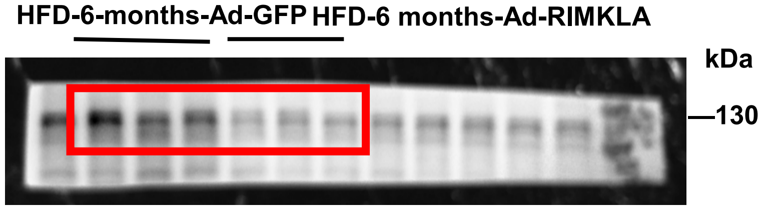

Suppl figure 4a: β-actin

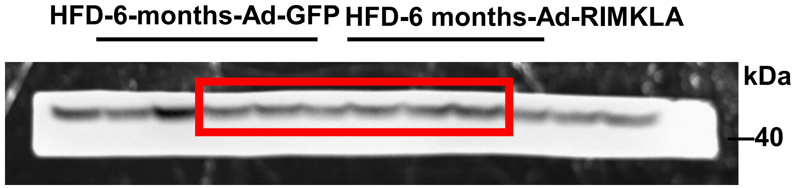

Suppl figure 4b: RIMKLA

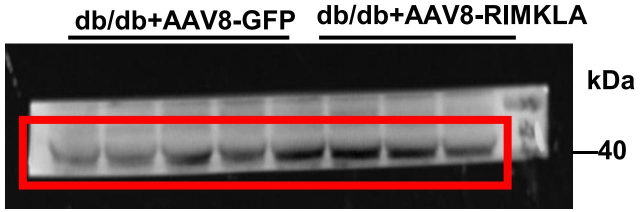

Suppl figure 4b: pAkt

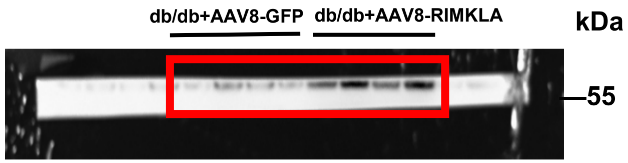

Suppl figure 4b: Akt

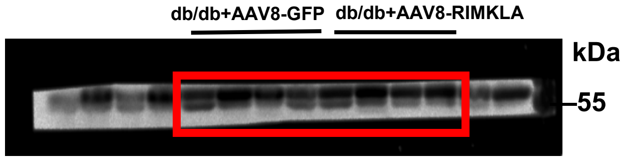

Suppl figure 4b: PEPCK

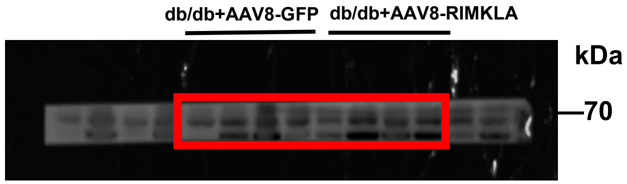

Suppl figure 4b: G6Pase

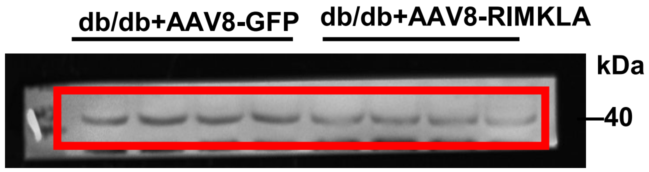

Suppl figure 4b: pFOXO1

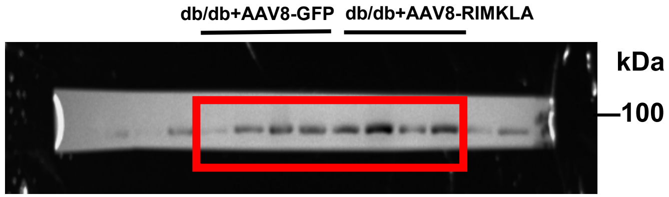

Suppl figure 4b: FOXO1

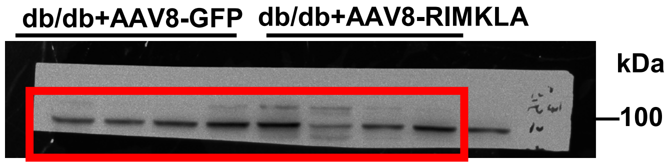

Suppl figure 4b: FASn

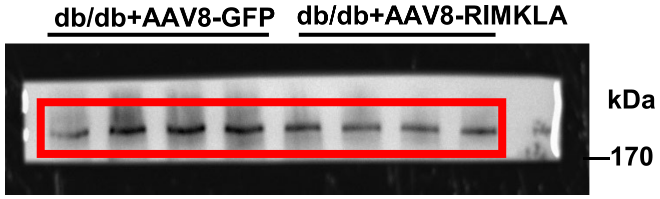

Suppl figure 4b: CD36

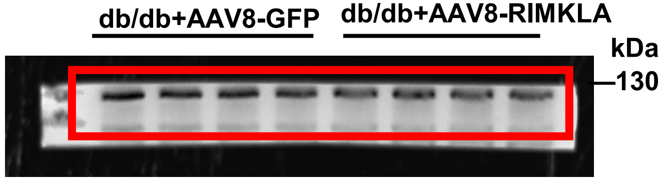

Suppl figure 4b: β-actin

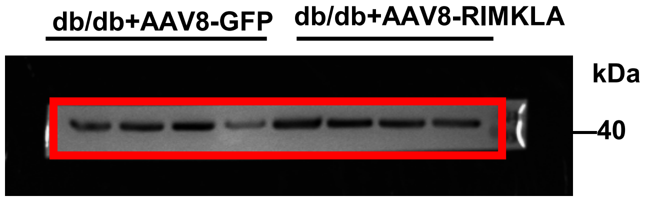

Suppl figure 5a: RIMKLA

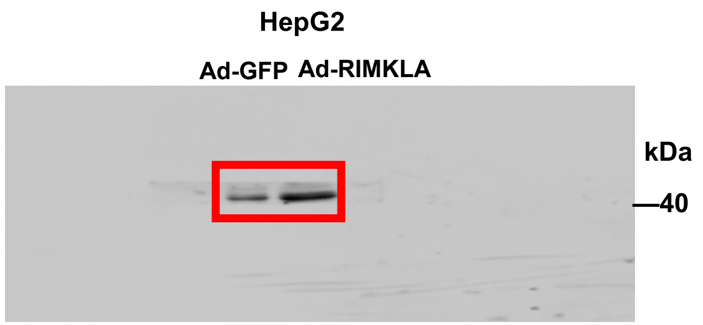

Suppl figure 5a: pFOXO1

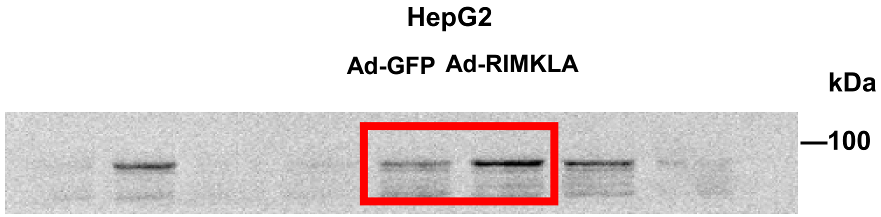

Suppl figure 5a: PEPCK

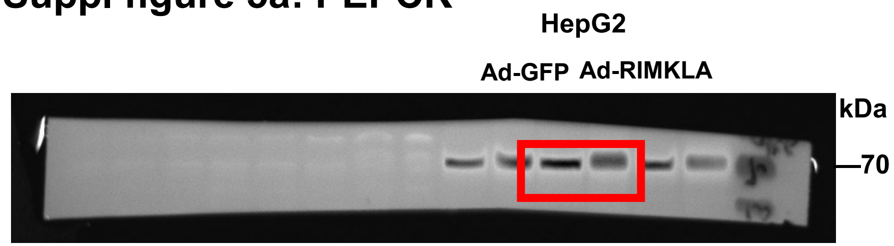

Suppl figure 5a: FOXO1

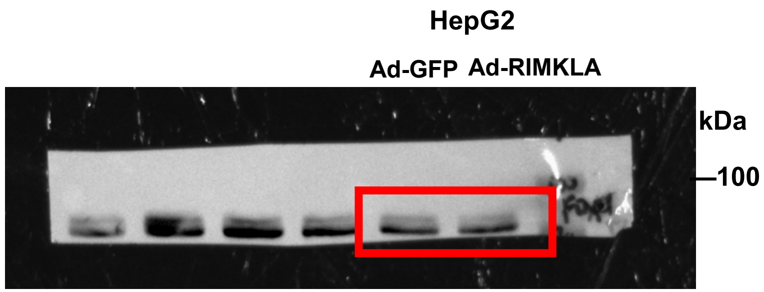

Suppl figure 5a: G6Pase

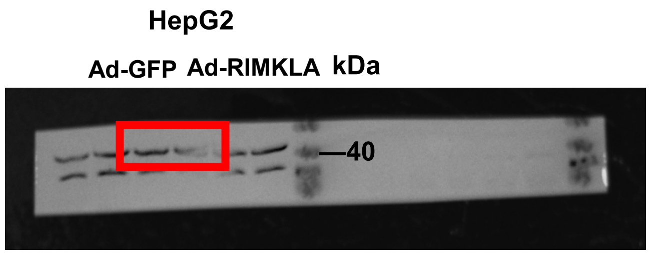

Suppl figure 5a: β-actin

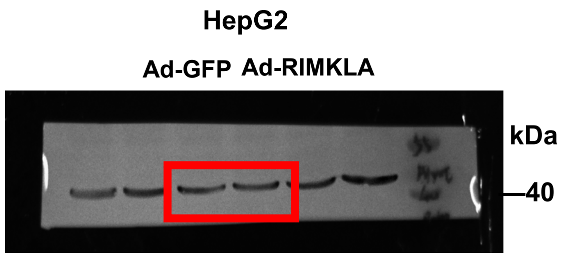

Suppl figure 6d: RIMKLA

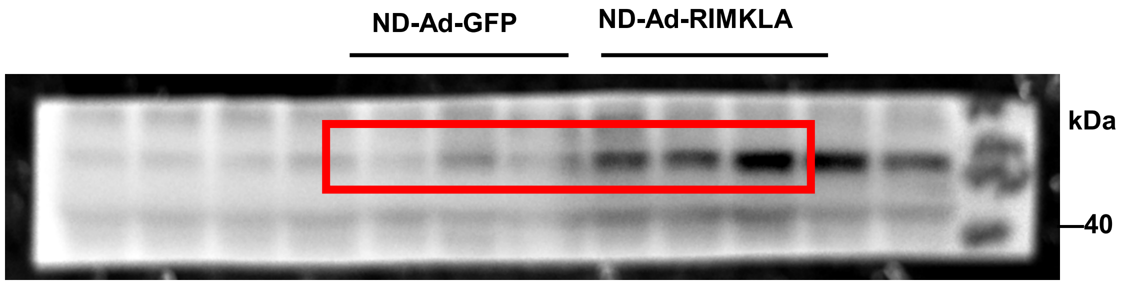

Suppl figure 6d: FASn

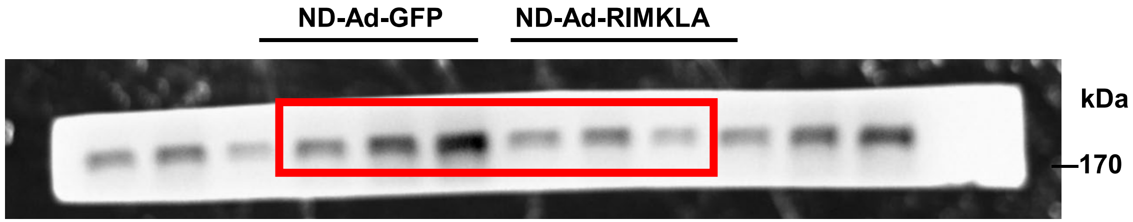

Suppl figure 6d: CD36

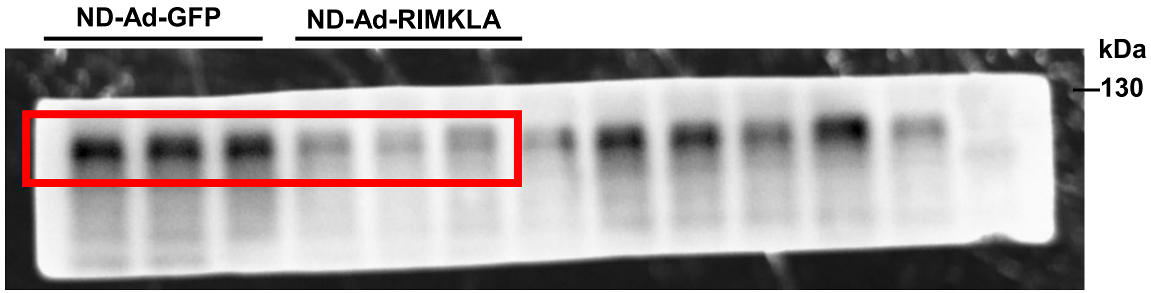

Suppl figure 6d: β-actin

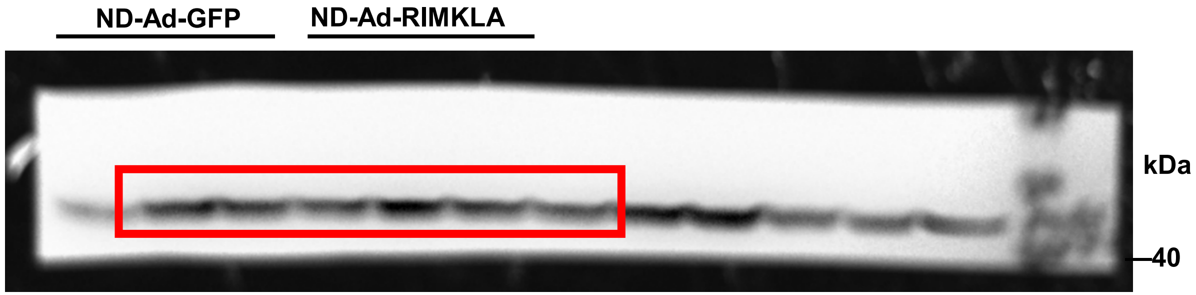

Suppl figure 9d: pBHMT1^{T45}

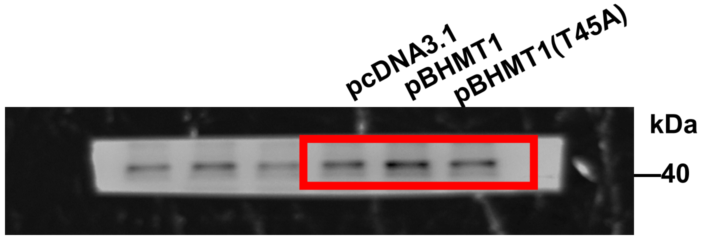

Suppl figure 9d: CD36

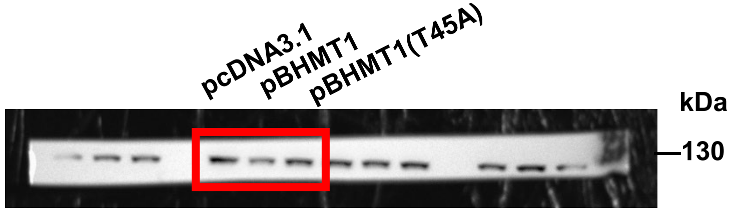

Suppl figure 9d: BHMT1

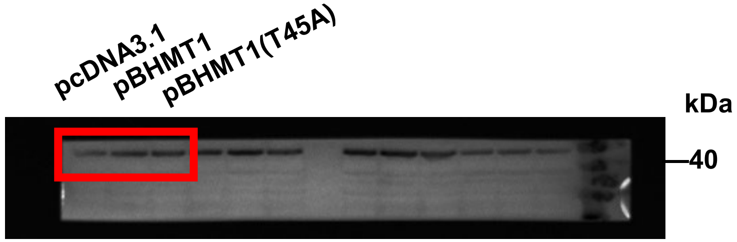

Suppl figure 9d: β-actin

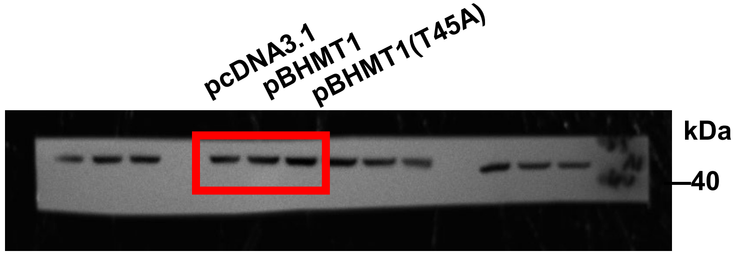

Suppl figure 9d: FASn

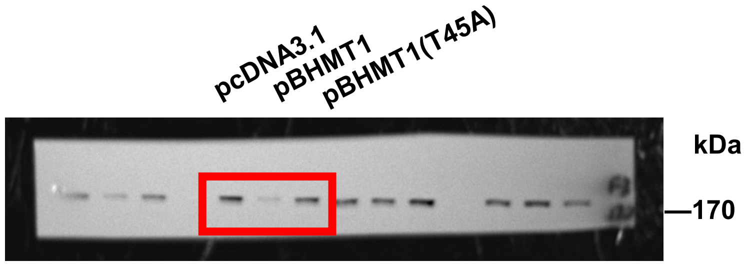

Suppl figure 9e: pBHMT1^{T45}

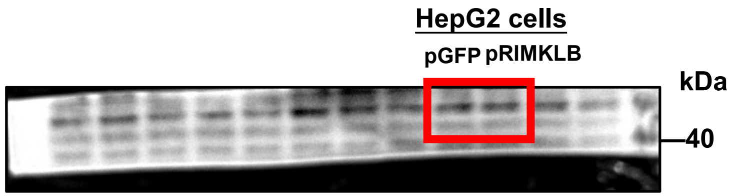

Suppl figure 9e: BHMT1

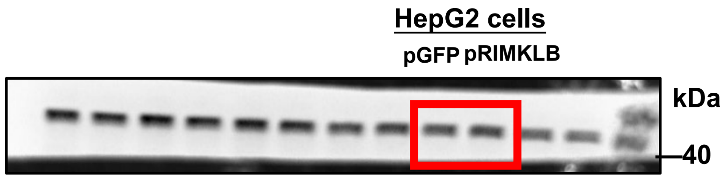

Suppl figure 9e: β-actin

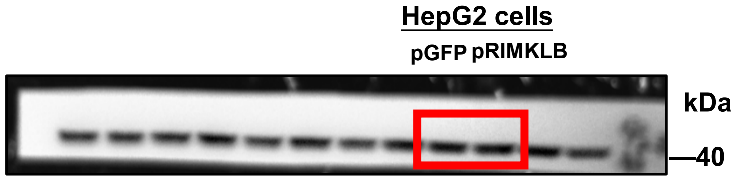

Suppl figure 9f: BHMT1(upper)

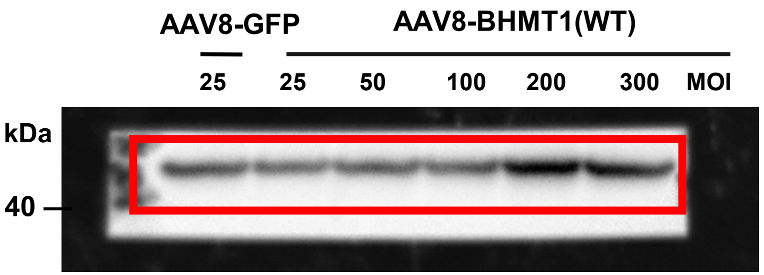

Suppl figure 9f: BHMT1(lower)

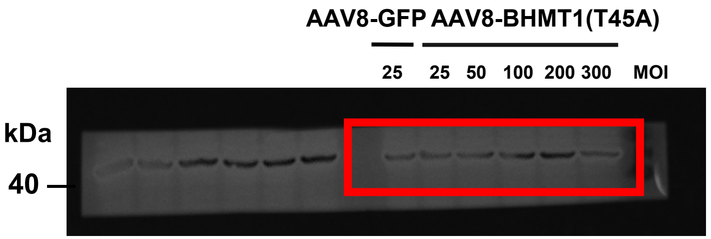

Suppl figure 9f: β-actin(upper)

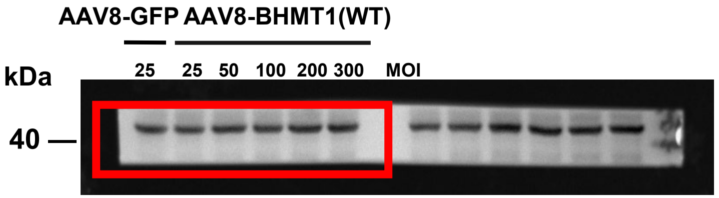

Suppl figure 9f: β-actin(lower)

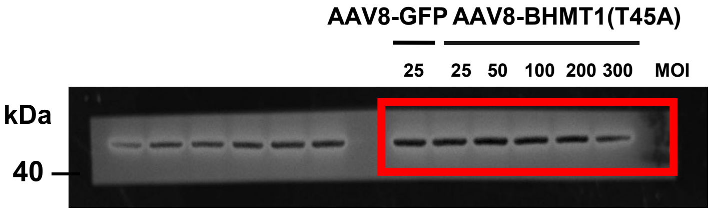

Suppl figure 12a: pBHMT1^{T45}

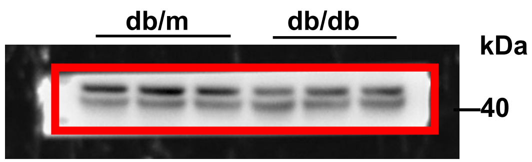

Suppl figure 12a: BHMT1

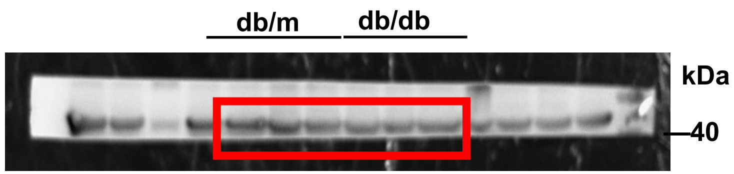

Suppl figure 12a: β-actin

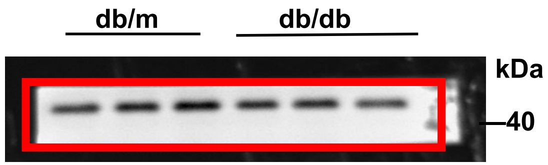

Suppl figure 14a: MTR

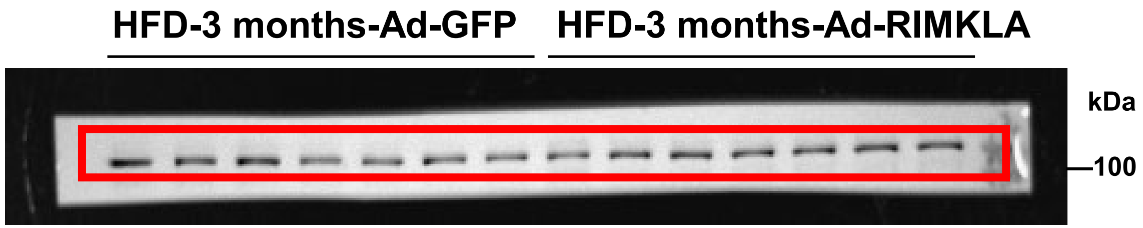

Suppl figure 14a: CBS

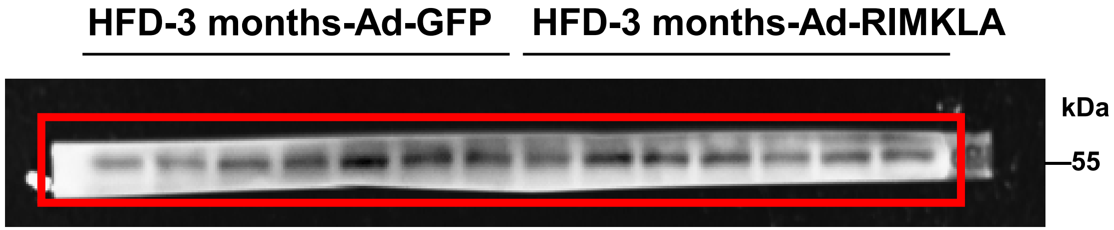

Suppl figure 14a: β-actin

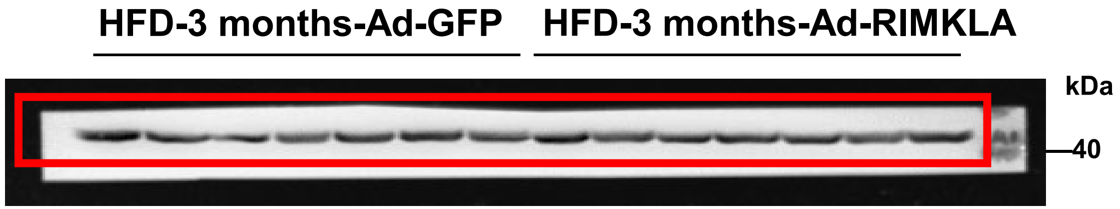

Suppl figure 14b: MTR

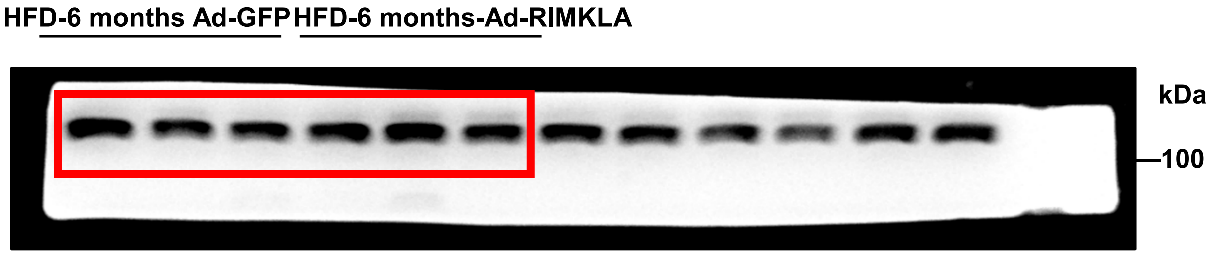

Suppl figure 14b: CBS

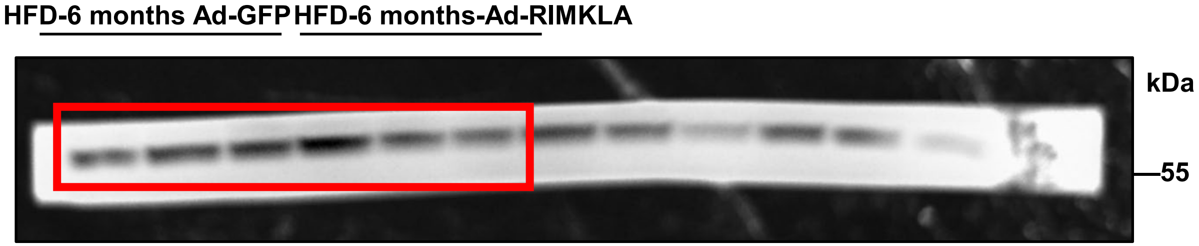

Suppl figure 14b: β-actin

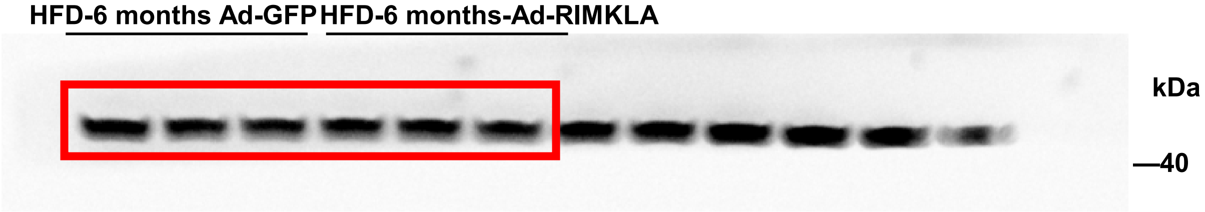

Suppl figure 14c: MTR

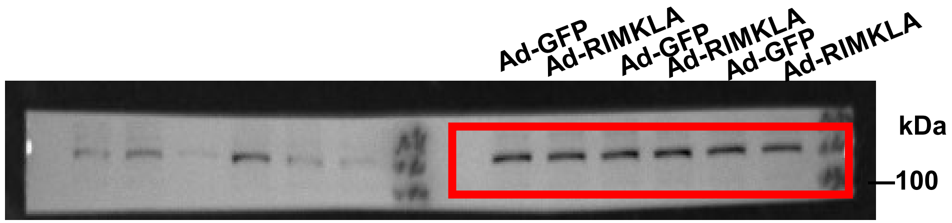

Suppl figure 14c: CBS

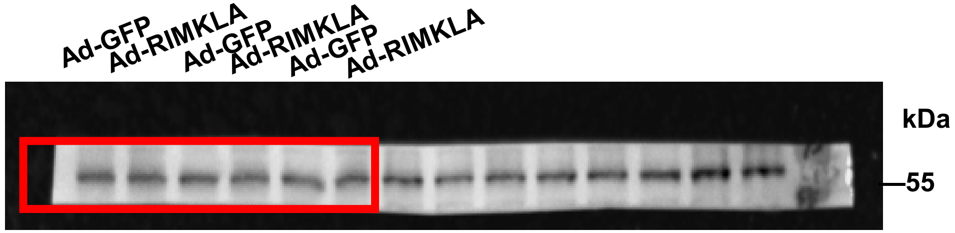

Suppl figure 14c: β-actin

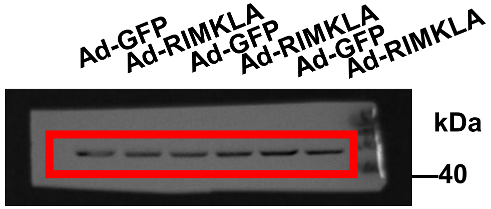

Suppl figure 18b: pAP1

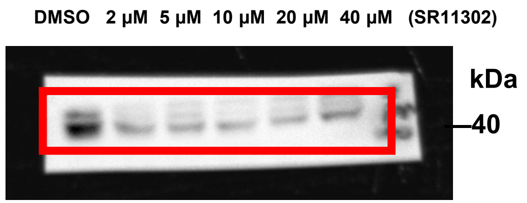

Suppl figure 18b: AP1

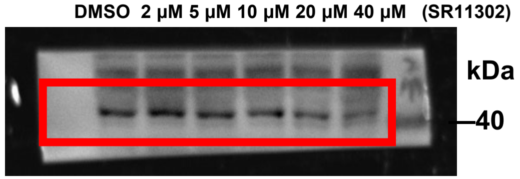

Suppl figure 18b: FASn

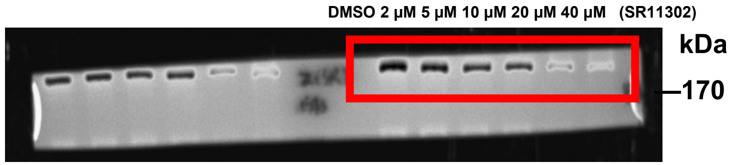

Suppl figure 18b: CD36

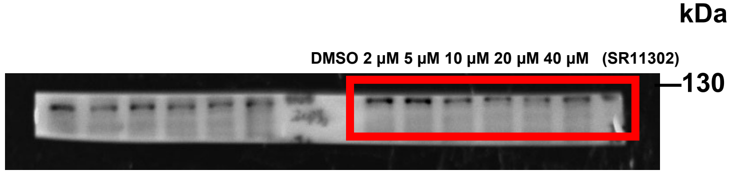

Suppl figure 18b: β-actin

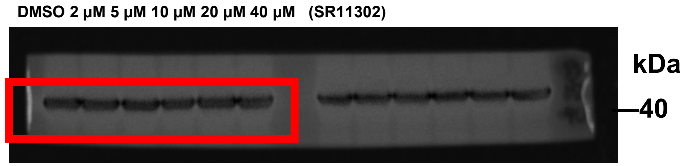

Suppl figure 18c: BHMT1

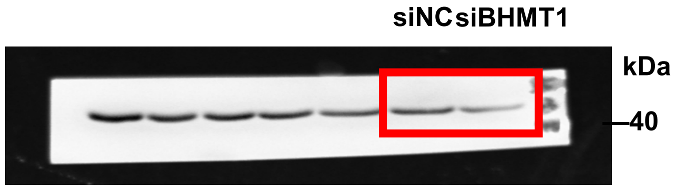

Suppl figure 18c: pAP1

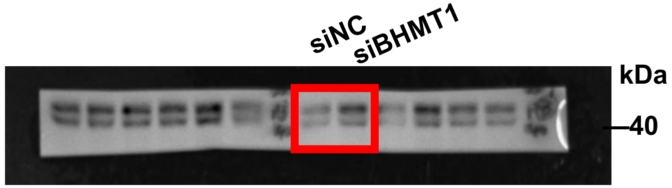

Suppl figure 18c: AP1

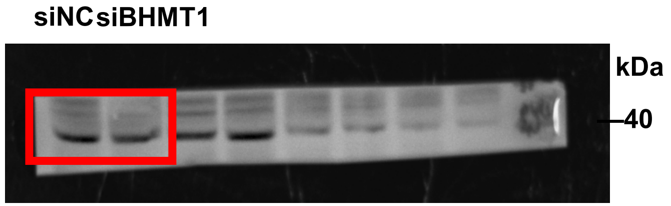

Suppl figure 18c: FASn

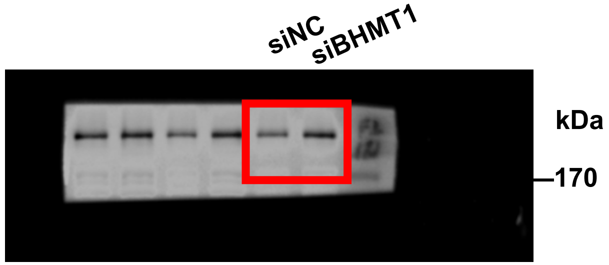

Suppl figure 18c: CD36

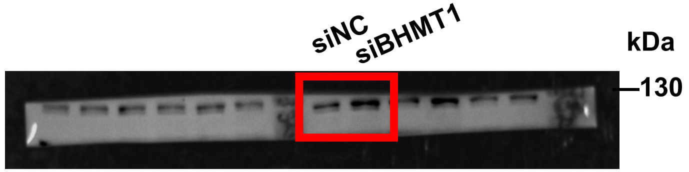

Suppl figure 18c: β-actin

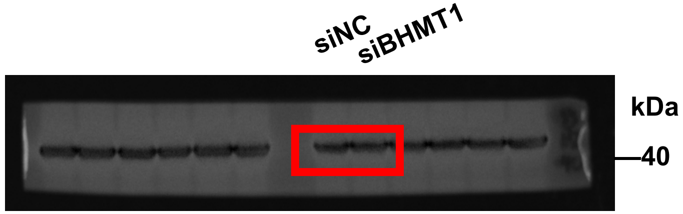

Suppl figure 19c: RIMKLA

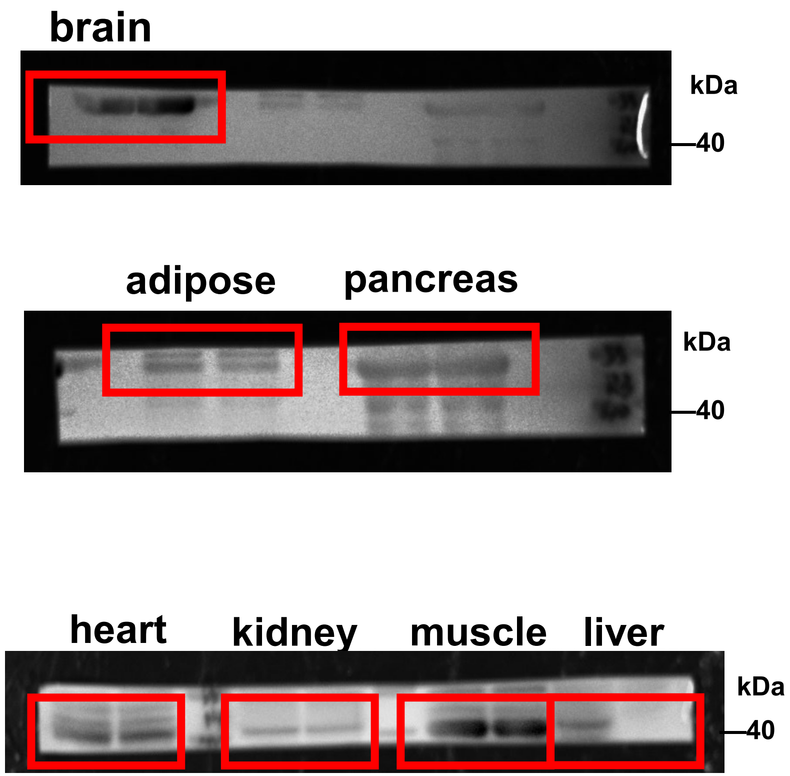

Suppl figure 19c: β-actin

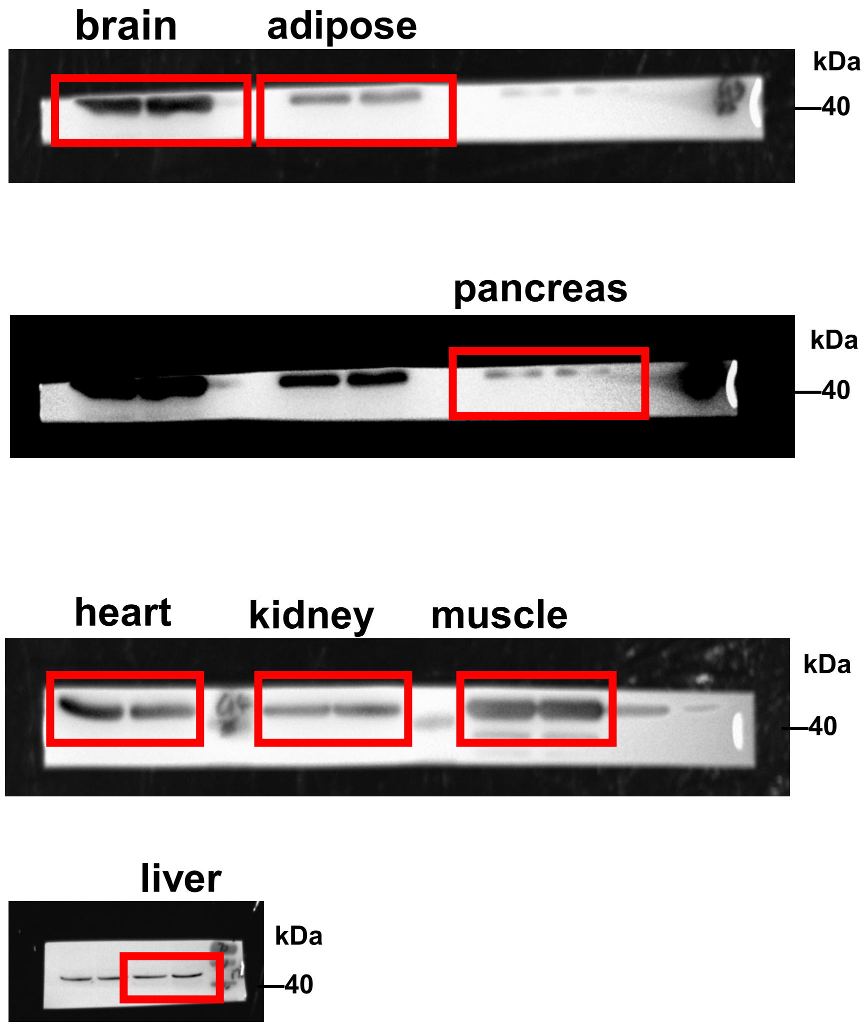

Figure 21i: FASn

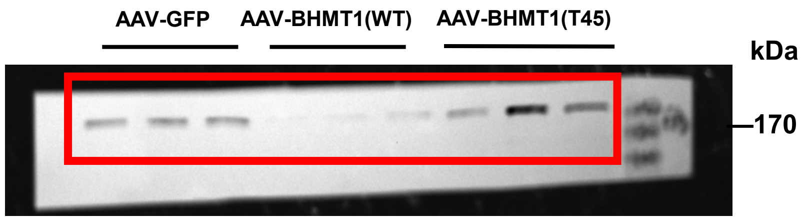

Figure 21i: CD36

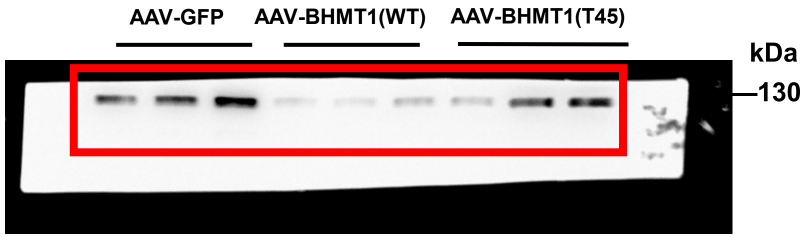

Figure 21i: pAP1

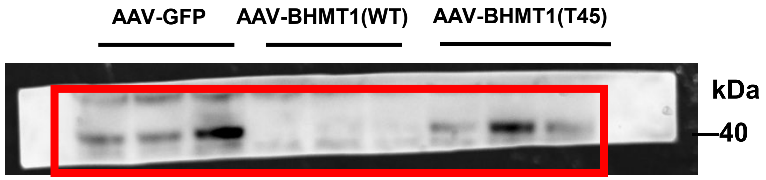

Figure 21i: AP1

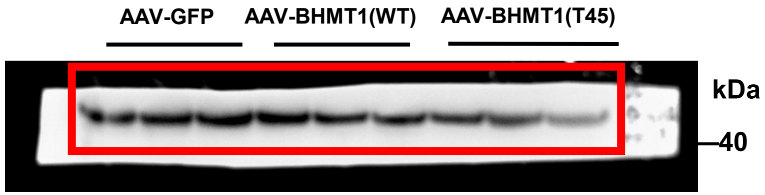

Figure 21i: BHMT1

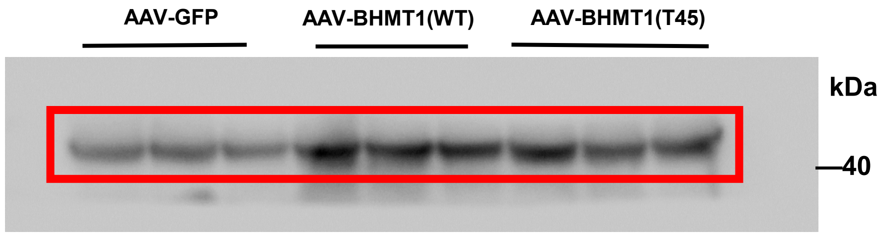

Figure 21i: pBHMT1^{T45}

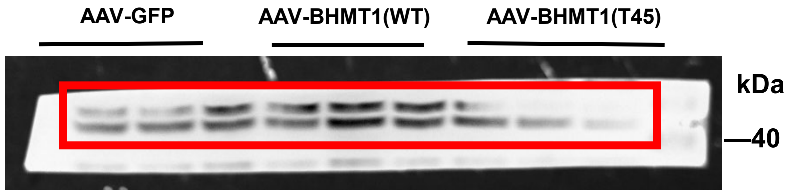

Figure 21i: β-actin

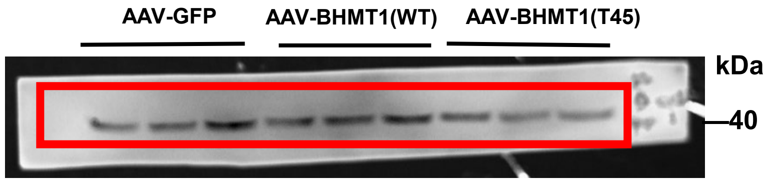

Suppl figure 22d: RIMKLA

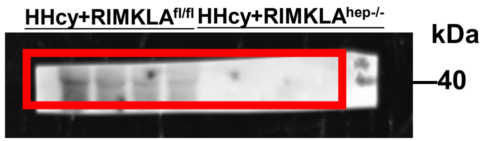

Suppl figure 22d: FASn

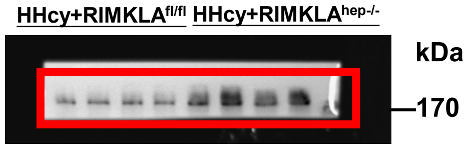

Suppl figure 22d: pAP1

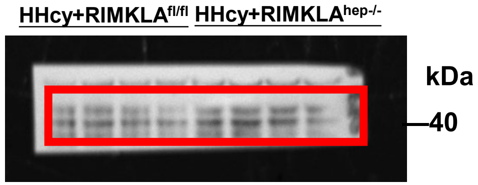

Suppl figure 22d: CD36

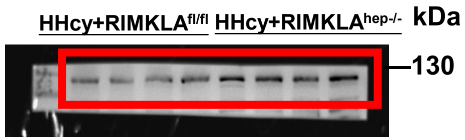

Suppl figure 22d: AP1

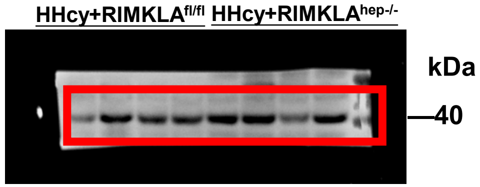

Suppl figure 22d: β-actin

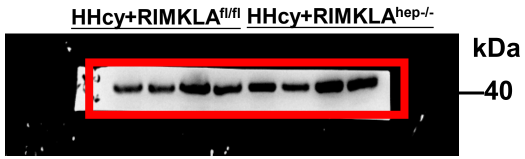

Supplement: Supplementary file 1 — Supplementary Materials [file 41392_2024_2054_MOESM1_ESM.pdf]
